# A network of small RNAs regulates sporulation initiation in *Clostridioides difficile*

**DOI:** 10.15252/embj.2022112858

**Published:** 2023-05-04

**Authors:** Manuela Fuchs, Vanessa Lamm‐Schmidt, Tina Lenče, Johannes Sulzer, Arne Bublitz, Janet Wackenreuter, Milan Gerovac, Till Strowig, Franziska Faber

**Affiliations:** ^1^ Helmholtz Centre for Infection Research (HZI) Helmholtz Institute for RNA‐based Infection Research (HIRI) Würzburg Germany; ^2^ Faculty of Medicine, Institute for Molecular Infection Biology (IMIB) Julius‐Maximilians‐University of Würzburg (JMU) Würzburg Germany; ^3^ Helmholtz Centre for Infection Research (HZI) Braunschweig Germany; ^4^ German Center for Infection Research (DZIF) Hannover–Braunschweig Germany

**Keywords:** *Clostridioides difficile*, Hfq, RIL‐seq, small RNA, Spo0A, Microbiology, Virology & Host Pathogen Interaction, RNA Biology

## Abstract

The obligate anaerobic, enteric pathogen *Clostridioides difficile* persists in the intestinal tract by forming antibiotic‐resistant endospores that contribute to relapsing and recurrent infections. Despite the importance of sporulation for *C. difficile* pathogenesis, environmental cues and molecular mechanisms that regulate sporulation initiation remain ill‐defined. Here, by using RIL‐seq to globally capture the Hfq‐dependent RNA–RNA interactome, we discovered a network of small RNAs that bind to mRNAs encoding sporulation‐related genes. We show that two of these small RNAs, SpoX and SpoY, regulate translation of the master regulator of sporulation, Spo0A, in an opposing manner, which ultimately leads to altered sporulation rates. Infection of antibiotic‐treated mice with SpoX and SpoY deletion mutants revealed a global effect on gut colonization and intestinal sporulation. Our work uncovers an elaborate RNA–RNA interactome controlling the physiology and virulence of *C. difficile* and identifies a complex post‐transcriptional layer in the regulation of spore formation in this important human pathogen.

## Graphical abstract

Small RNA/target interaction mapping in the enteric pathogen *C. difficile* reveals the first RNA chaperone Hfq‐dependent RNA interactome in a gram‐positive bacterium.

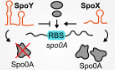

## Introduction

Since its discovery as a causative agent of antibiotic‐associated pseudomembranous colitis, *Clostridioides difficile* (*C. difficile*) has emerged as the leading cause of nosocomial antibiotic‐associated disease in the developed world (Bartlett *et al*, 1978; ECDC, 2018; CDC, 2019). Several virulence traits contribute to disease severity of *C. difficile* infections (CDI), including exotoxin production and spore formation (Smits *et al*, 2016). In particular, spores are a key element in host transmission and disease recurrence, due to their resistance to conventional antibiotics, disinfectants, and other environmental stressors (Peng *et al*, 2017; Zhu *et al*, 2018; O'Grady *et al*, 2021). Hence, understanding the environmental signals and molecular mechanisms that control spore formation in this important human pathogen is essential for the development of alternative treatment options.

Spore formation has been studied extensively in a variety of sporulating bacteria and represents an energetically costly, morphogenic process that is irreversible beyond a certain point in spore development (Edwards & McBride, 2014; Shen *et al*, 2019). In particular, sporulation initiation is tightly controlled through the integration of environmental and nutritional signals that mediate the post‐translational activation of the master regulator of sporulation, Spo0A (Deakin *et al*, 2012; Rosenbusch *et al*, 2012). However, *C. difficile* lacks many of the known conserved regulatory mechanisms that activate Spo0A, rendering sporulation initiation a poorly understood process in this gram‐positive pathogen (Lee *et al*, 2022). Once activated, phosphorylated Spo0A‐P acts as a transcriptional regulator that induces the expression of a set of early sporulation genes. This ultimately leads to the hierarchical activation of four compartment‐specific sigma factors—σ^E^/σ^K^ in the mother cell and σ^F^/σ^G^ in the forespore—and culminates in the formation of a metabolically dormant spore (Shen *et al*, 2019).

Most recently, post‐transcriptional regulation mediated by the RNA binding protein (RBP) Hfq has been implicated in modulating sporulation in *C. difficile* (Boudry *et al*, 2014; Maikova *et al*, 2019). Boudry *et al* demonstrated that depletion of Hfq leads to the upregulation of several sporulation‐related genes and an increased sporulation rate (Boudry *et al*, 2014; Maikova *et al*, 2019). Hfq is known for its ability to facilitate base‐pairing between small regulatory RNAs (sRNAs) and their target mRNAs, leading to altered translational efficiency and mRNA stability (Holmqvist & Vogel, 2018). Similar to its extensively studied gram‐negative counterparts, Hfq immunoprecipitation followed by sequencing of bound RNA species (RIP‐seq) in *C. difficile* uncovered a vast number of sRNAs and mRNAs bound by Hfq (Boudry *et al*, 2021; Fuchs *et al*, 2021). Furthermore, several sRNAs, not only in *C. difficile* but also in other spore‐forming Firmicutes, have been associated with the sporulation process, mostly through RNA‐seq and microarray‐based expression profiles (Silvaggi *et al*, 2006; Schmalisch *et al*, 2010; Marchais *et al*, 2011; Boudry *et al*, 2014). However, only a few of these sRNAs have been functionally described. In *C. difficile*, sRNA RCd1, which inhibits the production of the late mother cell‐specific sigma factor σ^K^, remains the only sporulation‐associated sRNA characterized to this date (Boudry *et al*, 2021), revealing a paucity of knowledge that clearly warrants further investigation.

Global approaches such as RIP‐seq are powerful tools in discovering RBP‐bound sRNAs or mRNAs (Fuchs *et al*, 2021; Lamm‐Schmidt *et al*, 2021). However, they rely on additional experimental and computational assays to identify directly interacting sRNA‐target pairs (Hör *et al*, 2018). Melamed and colleagues circumvented this difficulty by introducing RIL‐seq (RNA interaction by ligation and sequencing) to the field of bacterial RNA‐biology (Melamed *et al*, 2016, 2018). Similar to CLASH and hiCLIP, RIL‐seq relies on ligation of RBP‐bound RNA pairs and thereby directly captures and identifies interaction partners (Helwak *et al*, 2013; Kwok, 2016).

In this study, we applied Hfq RIL‐seq to *C. difficile*, which led to the discovery of an extensive Hfq‐mediated sRNA‐target network. Among the identified sRNA–mRNA interactions were several sRNAs bound to the *spo0A* mRNA, encoding the master regulator of sporulation. We show that two of these sRNAs, SpoY and SpoX, regulate *spo0A* translation in an opposite manner *in vivo*, resulting in altered sporulation rates. Furthermore, SpoY and SpoX deletion significantly impacts *C. difficile* gut colonization and spore burden in a mouse model of *C. difficile* infection. Overall, we provide the first example of sRNAs regulating sporulation initiation by finetuning *spo0A* translation, which adds a new layer of post‐transcriptional regulation to the complex process of sporulation initiation in this important human pathogen.

## Results

### Hfq is a global RNA binding protein that mediates sRNA‐mRNA interactions in *C. difficile*

**Figure 1:**
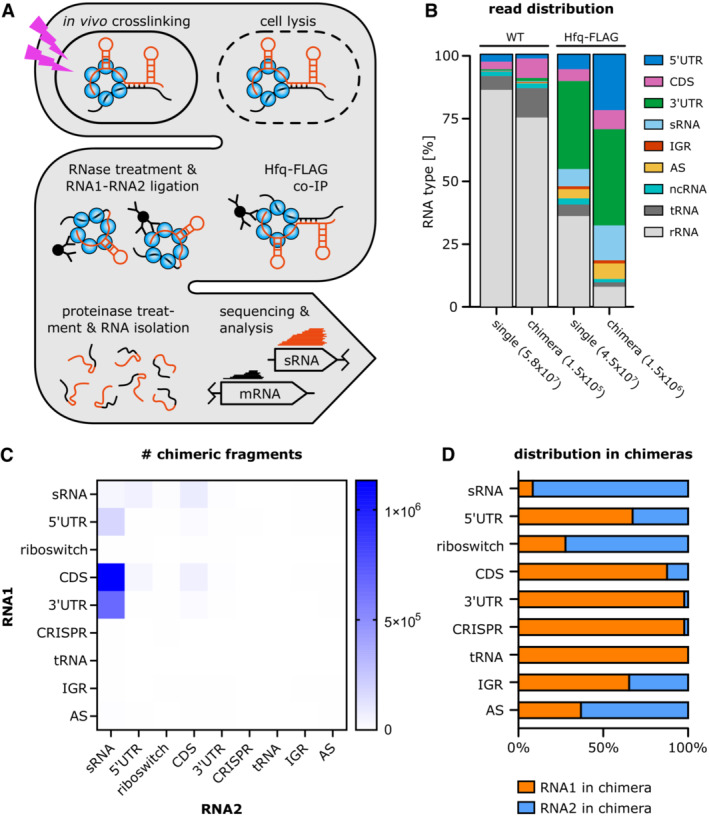
RIL‐seq establishes Hfq as a platform for RNA‐RNA interactions in *C. difficile* ASchematic representation of the RIL‐seq workflow.BDistribution of all reads, single and chimeric, across all RNA classes, comparing Hfq‐FLAG and control (WT) strain (*n* = 4 each). ncRNAs include riboswitches, tmRNA, SRP RNA, RNase P RNA and, 6S RNA. All chimeras were included, without filtering for statistical significance or manual curation.C, DDistribution of RNA classes in chimeric fragments based on Dataset EV3, where RNA1 constitutes the 5′ end and RNA2 the 3′ end of a chimera. Only statistically relevant interactions (Fisher′s exact test ≤ 0.05) that are represented by at least 25 chimeric fragments are included.

To better understand the impact of post‐transcriptional regulation on sporulation, we performed Hfq RIL‐seq in *C. difficile* in sporulating conditions (Melamed *et al*, 2018). *C. difficile* 630 cells expressing a chromosomally FLAG‐tagged Hfq variant (Hfq‐FLAG, *n* = 4) were harvested during the transition phase (early stationary phase), when *C. difficile* shifts to a nongrowing state, accompanied by sporulation to ensure survival in nutrient limiting conditions (Saujet *et al*, 2011; Hofmann *et al*, 2018). Harvested cells were UV‐crosslinked to stabilize *in vivo* protein–RNA interactions, followed by cell lysis and Hfq co‐immunoprecipitation. Identification of Hfq‐associated RNA–RNA interaction partners was achieved by ligation of Hfq‐bound RNA pairs (“chimeras”), followed by RNA purification, sequencing, and computational analysis using a previously published primary transcriptome annotation of *C. difficile* 630 (Fig 1A; Fuchs *et al*, 2021). *C. difficile* 630 expressing native Hfq (WT) served as a control and was treated similarly (*n* = 4). Analysis of the RIL‐seq data revealed a high number of Hfq‐bound single and chimeric fragments with a considerable enrichment of chimeric reads in the Hfq‐FLAG strain, when compared to the WT (Fig 1B). The list of chimeras was manually curated and further reduced to statistically relevant interactions (Odds ratio ≥ 1 and *P*‐value < 0.05) that are represented by at least 25 chimeric fragments (Melamed *et al*, 2018). All remaining interactions are listed in Datasets EV2 and EV3. The resulting RIL‐seq network is publicly available and explorable in an RNA–RNA interactome browser (https://resources.helmholtz‐hiri.de/rilseqcd/, Appendix Fig S2). In accordance with existing *E. coli* and *S. enterica* RIL‐seq data, most chimeras (67%) consisted of mRNA–sRNA interactions, with mRNAs (5′UTR, CDS or 3′UTR) at position 1 (RNA1/5′ end) and sRNAs at position 2 (RNA2/3′ end), as shown in Fig 1C and D and Appendix Fig S1B. Although most sRNAs were predominantly found at position 2, such as nc159, a few sRNAs showed a clear preference for position 1, including nc083 (Appendix Fig S1B). However, of all chimeric fragments mapping to sRNAs, more than 90% mapped to RNA2 (Fig 1D). This position bias reflects the mechanism by which most sRNAs bind Hfq in gram‐negative species. Interactions generally occur between the proximal face of Hfq and the distinct intrinsic terminator and poly‐U tail that characterizes most sRNAs, ultimately rendering the sRNA 3′ end inaccessible to proximity ligation (Fig 1A; Park *et al*, 2021). Accordingly, our data imply that *C. difficile* Hfq might employ binding mechanisms similar to those described for *S. enterica* and *E. coli* in facilitating sRNA target interactions (Park *et al*, 2021). While the majority of sRNAs were found ligated to CDSs (56%), a surprisingly high number (33%) interacted with mRNA 3′UTRs (Fig 1C). Recently published *C. difficile* Hfq RIP‐seq data revealed similar distributions of Hfq‐bound RNA species; however, RIP‐seq does not allow identification of direct interaction partners (Fuchs *et al*, 2021). In contrast, sRNA–mRNA chimeras in *E. coli* and *S. enterica* were clearly dominated by sRNAs interacting with CDSs or 5′UTRs, while sRNA‐3′UTR ligations were barely found (Melamed *et al*, 2016; Matera *et al*, 2022). Although bacterial 5′UTRs have long been described as the prototypical target of sRNA‐mediated post‐transcriptional regulation, there are examples of sRNAs targeting mRNA 3′UTRs, including sRNA Spot42 targeting the 310‐nt long *hilD* 3′UTR, a transcriptional regulator of virulence in *S. enterica* (El Mouali *et al*, 2018; Bronesky *et al*, 2019; Menendez‐Gil & Toledo‐Arana, 2021). Indeed, research on *S. aureus* suggests that long 3′UTRs in particular might be an underrated source of regulatory elements that impact transcript stability and translation (Ruiz de los Mozos *et al*, 2013; Menendez‐Gil & Toledo‐Arana, 2021). Considering that in *C. difficile*, 42% of all annotated 3′UTRs are longer than 100 nt, they might constitute a source of regulatory elements targeted by sRNAs (Fuchs *et al*, 2021).

### Hfq RIL‐seq identifies novel sRNA candidates

**Figure 2:**
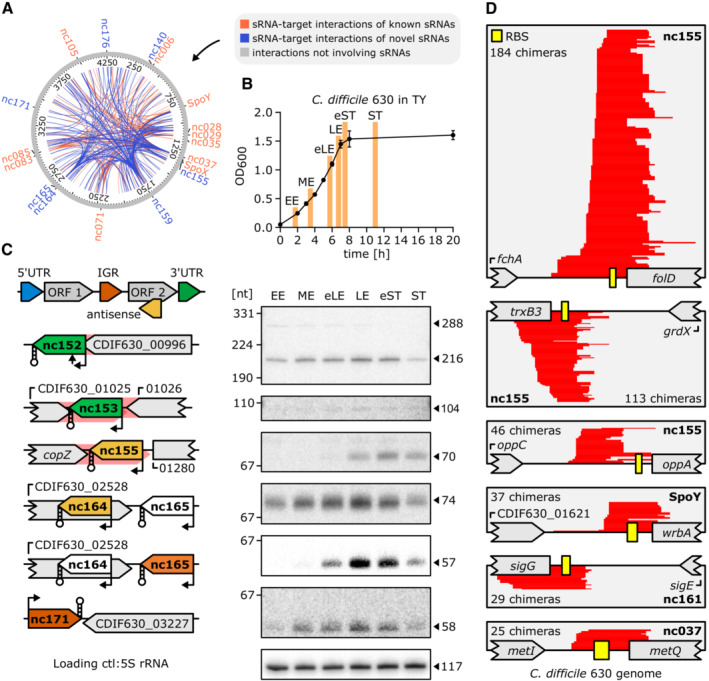
RIL‐seq analysis facilitates annotation of novel sRNAs and reveals sRNA‐mediated regulation of polycistronic transcripts ACircos plot of all RIL‐seq interactions that are represented by ≥ 200 chimeric fragments, mapped to the *C. difficile* 630 chromosome. Interaction hubs characterized by sRNAs with ≥ 20 unique interactions are labeled. Interactions involving known sRNAs are marked in orange, while interactions involving new sRNAs are highlighted in blue. All other interactions are gray.B, CNorthern blot validation of new sRNAs. (B) Samples were taken in early exponential (EE), mid‐exponential (ME), early late exponential (eLE), late exponential (LE), early stationary (eST), and stationary (ST) phase of growth in TY medium. The error bars represent the mean ± SD of four biological replicates (C) sRNAs are color‐coded according to their genomic location. Stem‐loop structures indicate intrinsic terminators, arrows indicate TSSs or processing sites, and red shadings untranslated regions (Fuchs *et al,* 2021). A representative image of three independent experiments is shown.DCoverage plots of several sRNA‐IGR chimeras, where the IGR is located within poly‐cistronic operons. Chimeric reads are highlighted in red; the RBS is depicted as a yellow box. The number of chimeras covering each interaction is provided and the respective interacting sRNA is highlighted in bold. Source data are available online for this figure.

Recent publications suggest that RIL‐seq network data can be exploited to identify new sRNAs by taking into account unique features of sRNAs in general and sRNA RIL‐seq chimeras in particular (Bar *et al*, 2021; Matera *et al*, 2022). Accordingly, a high number of chimeric fragments mapping to a single RNA has been identified as a promising indicator of potential new sRNAs (Bar *et al*, 2021). This is reflected in the formation of “interaction hubs” that consist of a dominating, single RNA interacting with a large number of unique RNAs (Matera *et al*, 2022). By mapping all chimeric fragments to the *C. difficile* genome, we could identify 24 interaction hubs formed by sRNA candidates that were previously unknown (*n* = 15) or non‐validated (*n* = 9) sRNAs (Fig 2A; Dataset EV4; Chen *et al*, 2011; Soutourina *et al*, 2013; Fuchs *et al*, 2021). Subsequent northern blot analysis of sRNA expression during different growth stages (Fig 2B) confirmed the expression of six of eight tested sRNA candidates (Fig 2C). Expression profiles of these sRNAs indicated expression mainly during late exponential/early stationary growth phase (Fig 2C), coinciding with the growth stage selected for our RIL‐seq experiment (early stationary). Accordingly, performing RIL‐seq in distinct growth conditions has the potential to uncover novel sRNA candidates that have evaded previous detection approaches such as RIP‐seq, due to its unique ability to reveal both Hfq‐association and RNA–RNA interaction (Melamed *et al*, 2016).

### RIL‐seq data suggest sRNA‐mediated discoordinate regulation of operons in *C. difficile*

In addition to the vast number of chimeras representing sRNA–mRNA pairs (*n* = 1,046), sRNA‐encompassing interactions also included chimeras consisting of sRNA–sRNA (*n* = 39) and sRNA–IGR (intergenic region) ligations (*n* = 24, Dataset EV2). While sRNA–sRNA pairs have been discussed as a pool of potential sRNA sponges, sRNA–IGR chimeras have not been investigated previously (Melamed *et al*, 2016; Małecka *et al*, 2021; Matera *et al*, 2022). A detailed analysis of those interactions revealed that in several cases (*n* = 8, 33%), “IGRs” represented noncoding regions in polycistronic mRNAs (Fig 2D). To further understand the impact of sRNA‐mediated regulation on operon expression in *C. difficile*, we compared our RIL‐seq data with previously published operon annotations (Fuchs *et al*, 2021). We found that 383 RIL‐seq chimeras mapped to 170 of 400 known operons. Of these 383 chimeras, 98 constituted sRNAs interacting with intra‐operon ribosome binding site (RBS) regions (25 nt up‐ and 20 nt downstream of the respective start codon), indicating potentially widespread coordinate and discoordinate regulation of polycistronic mRNAs in *C. difficile* (Dataset EV5). While sRNA‐mediated coordinate regulation of entire operons is more common, there are several reports of sRNAs targeting individual genes in polycistronic mRNAs, thereby only affecting a subset of genes within an operon (discoordinate regulation; Balasubramanian & Vanderpool, 2013; Rice *et al*, 2012). For instance, in *E. coli*, RyhB targets the *iscRSUA* operon, selectively inhibiting translation of *iscS* and resulting in the degradation of the *iscSUA* part (Møller *et al*, 2002; Desnoyers *et al*, 2009). Hence, discoordinate regulation of operons allows bacteria to selectively produce operon components, for example, when only a specific gene product is needed in a given condition (Balasubramanian & Vanderpool, 2013). In line, our RIL‐seq dataset comprised chimeras formed by the RBS region of the sporulation‐specific sigma factor *sigG* and the newly annotated sRNA CDIF630nc_161 (Fig 2D). *sigG* encodes the forespore‐specific late sporulation sigma factor σ^G^ and constitutes the last gene in an operon formed by two additional sporulation‐specific genes, including *sigE* directly upstream of *sigG* (Saujet *et al*, 2014). In contrast to σ^G^, the *sigE*‐ encoded sigma factor σ^E^ is active in the mother cell during early sporulation. Accordingly, both sigma factors not only operate during different stages of sporulation, but also in different compartments, and consequently require a tight regulation (Saujet *et al*, 2014). Hence, sRNA‐mediated post‐transcriptional regulation might fine‐tune the sequential expression of both sigma factors to ensure correct spore development. Nevertheless, a more detailed analysis is needed to fully understand the nature and extent of these regulatory events in *C. difficile,* not only on sporulation but on cellular processes in general.

### The master regulator of sporulation, Spo0A, is a central target of sRNA‐based regulation

**Figure 3:**
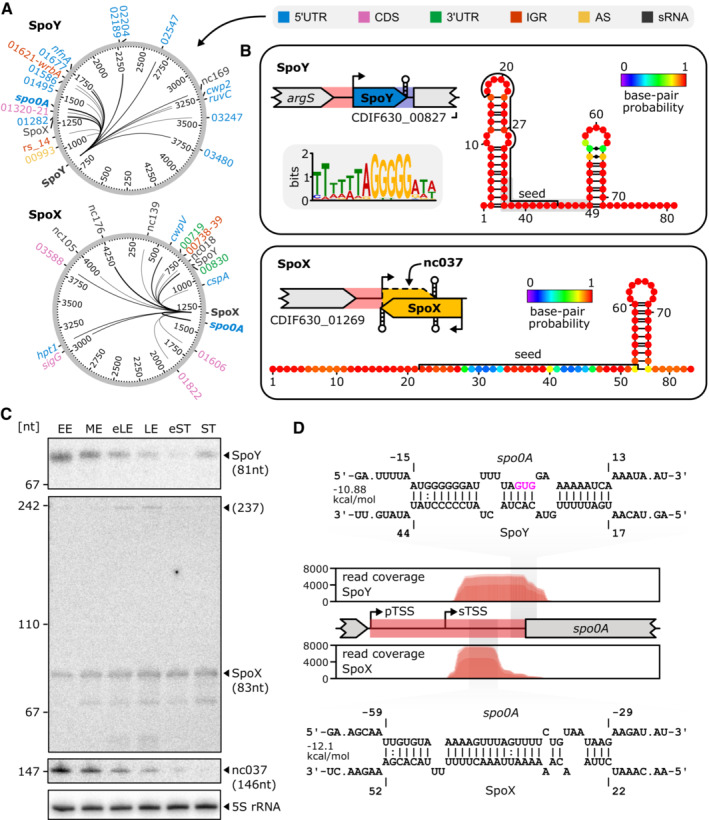
RIL‐seq reveals *spo0A* as a target of sRNA‐mediated post‐transcriptional regulation ACircos plots highlighting the SpoY and SpoX interactome, only interactions supported by ≥ 25 chimeras are included. Target types are discriminated by color. Edge strength correlates with the number of chimeras supporting an individual interaction.BGenomic location and predicted secondary structure (RNAfold; Lorenz *et al*, 2011) of SpoY and the short SpoX isoform are provided. Seed regions relevant for *spo0A* interaction were predicted *in silico* (IntaRNA; Mann *et al*, 2017) and are labeled in the secondary structure. For both sRNAs, target sequences were extracted from the RIL‐seq data and uploaded to MEME (Bailey & Elkan, 1994), resulting in the successful identification of a common SpoY target motif (present in 24/28 target sequences).CNorthern blot validation of SpoY, SpoX, and nc037 expression in early exponential (EE), mid‐exponential (ME), early late exponential (eLE), late exponential (LE), early stationary (eST), and stationary (ST) phase of growth in TY medium. 5S rRNA served as a loading ctl. A representative image of three independent experiments is shown.DRead coverage of *spo0A* by SpoY‐*spo0A* (top) and SpoX‐*spo0A* (bottom) chimeric reads. Base pairing information and location of predicted interaction sites (IntaRNA; Mann *et al*, 2017) are highlighted. The *spo0A* nucleotide position is calculated relative to the *spo0A* start codon (highlighted in pink). The sigA‐dependent primary transcription start site (pTSS) and sigH‐dependent secondary TSS are marked. Source data are available online for this figure.

**Figure EV1:**
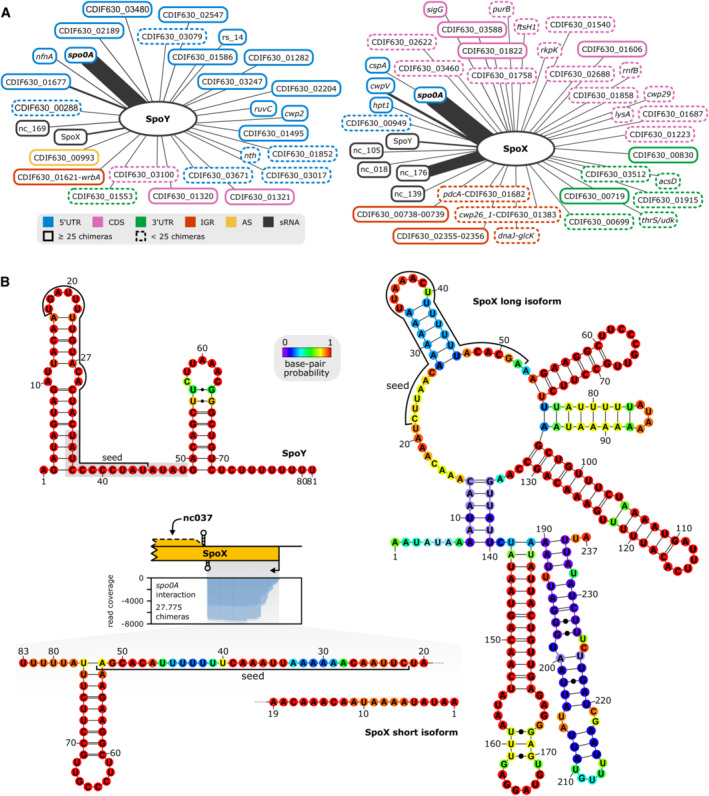
RIL‐seq reveals *spo0A* as a target of sRNA‐mediated post‐transcriptional regulation ATarget network of SpoY and SpoX, targets supported by ≥ 25 chimeras are marked by a solid line, while targets supported by < 25 chimeras are highlighted with a dashed line. Target types are discriminated by color. Edge strength correlates with the number of chimeras supporting an individual interaction.BPredicted secondary structure (RNAfold; Lorenz *et al*, 2011) for SpoY and both isoforms of SpoX are provided. Seed regions relevant for *spo0A* interaction were predicted *in silico* (IntaRNA; Mann *et al*, 2017) and emphasized in the secondary structure. Read coverage of SpoX by SpoX‐*spo0A* chimeric reads is highlighted in relation to the SpoX‐encoding region.

Interestingly, *sigG* was only one among several sporulation‐specific genes enriched in our RIL‐seq dataset. Additional sporulation‐related genes included *sigE*, *sleB*, *spoIIAB*, *spoIVA*, and *spo0A*, encoding the master regulator of sporulation (Dataset EV2; Deakin *et al*, 2012). The latter was of particular interest since chimeras comprising *spo0A* and the sRNA nc020 as well as *spo0A* and sRNA nc038 were among the top five most abundant RIL‐seq interactions in the entire dataset (> 20,000 chimeras each). We decided to investigate these interactions in more detail and renamed both sRNAs to SpoY (nc020) and SpoX (nc038), to reflect their involvement in sporulation. SpoY is a 5′UTR‐derived sRNA, sharing its transcription start site with CDIF630_00827, which encodes a protein of unknown function (Fig 3A and B). Northern blot analysis indicated a complex expression profile with the highest SpoY expression during the early‐ and mid‐exponential growth phases, decreasing levels toward early stationary growth and increasing expression following entry into stationary phase when grown in TY (Fig 3C, growth phases are indicated in Fig 2B). We also monitored expression in additional media that are known to impact sporulation frequency, including BHI, TYG, TYF, and 70:30 medium, which revealed expression of SpoY over a broad range of conditions (Appendix Fig S3). MEME analysis of SpoY RIL‐seq chimeras, including the *spo0A* interaction, suggested that SpoY preferably binds mRNA 5′UTRs at a G‐rich target motif that resembles the RBS (Figs 3A and B, EV1A and B; Bailey & Elkan, 1994). Indeed, *in silico* predictions of RNA–RNA interactions performed with IntaRNA indicated that SpoY binding blocks the *spo0A* RBS, which typically leads to translational inhibition (Fig 3D; Bernhart *et al*, 2006; Mann *et al*, 2017).

**Figure EV2:**
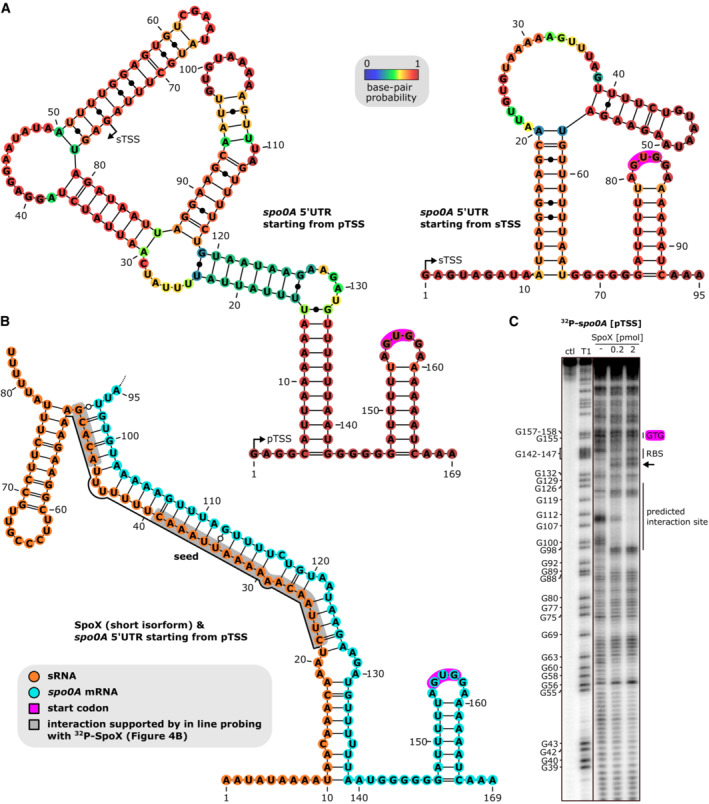
SpoX binding potentially renders the *spo0A* RBS region more accessible APredicted secondary structures of the *spo0A* 5′UTR and beginning of CDS, starting from the primary transcription start site (pTSS) and secondary TSS (RNAfold; Lorenz *et al*, 2011). The start codon is highlighted in pink.BPredicted secondary structures of the *spo0A* 5′UTR and beginning of CDS upon dimer formation with SpoX (shown is the short isoform). Resulting dimer for the depicted region looks identical, independent of which SpoX isoform or *spo0A* 5′UTR length was used (RNAcofold; Bernhart *et al*, 2006). sRNA and mRNA are highlighted in orange and blue, respectively. sRNA‐target base‐pairings supported by in‐line probing (Fig 4B) are shaded in gray.CIn‐line probing of 0.2 pmol of ^32^P‐labeled *spo0A* (starting from pTSS) in the absence (lane 3) or presence of increasing concentrations (lane 4&5) of SpoX (short isoform). RNase T1 digested *spo0A* serves as a ladder. Start codon and predicted seed region are highlighted. A representative image of three independent experiments is shown.

In contrast to SpoY, SpoX is encoded partially antisense to another putative sRNA, nc037 (Fig 3B). Interestingly, previously published RNA‐seq data revealed an intrinsic terminator within the SpoX sequence, resulting in a short (83 nt) and long isoform (237 nt, Figs 3B and EV1B; Fuchs *et al*, 2021). The long isoform is encoded antisense to nc037, while the short isoform terminates prior to the overlapping region. According to northern blot analysis, the short SpoX isoform is far more prevalent and expressed through all growth phases, while the long isoform appears barely expressed and is only detectable during late exponential growth phase (Fig 3C). Additional expression profiling in various media further indicated that SpoX expression tends to be upregulated in stationary phase (Appendix Fig S3). Interestingly, expression of the putative sRNA nc037 in TY is mostly anticorrelated (Fig 3C) to the expression of SpoX, potentially influencing expression of the long SpoX isoform. Further analyses will be necessary to assess the impact of nc037 on SpoX expression. The target spectrum of SpoX is diverse, including several CDSs and sRNAs in addition to the *spo0A* 5′UTR (Figs 3A and EV1A). Consequently, identification of a conserved target motif using MEME was not successful (Bailey & Elkan, 1994). According to IntaRNA analysis and the peak profile of SpoX‐*spo0A* chimeric reads, SpoX binds further upstream in the *spo0A* 5′UTR (Fig 3D; Mann *et al*, 2017). *In silico* predictions of the secondary structures of SpoX and the *spo0A* mRNA upon duplex formation, suggested that SpoX‐*spo0A* base pairing disrupts the *spo0A* 5′UTR secondary structure, potentially rendering the RBS more accessible to ribosome binding (Fig EV2A and B). In‐line probing using ^32^P‐*spo0A* in combination with SpoX further supported this hypothesis (Fig EV2C). Of note, the predicted SpoX interaction site (seed region) involved in *spo0A* base‐pairing is located at the 5′ end of the SpoX sRNA and therefore present in both SpoX isoforms (Figs 3B and D and EV1B; Mann *et al*, 2017). However, SpoX‐*spo0A* chimeric reads solely mapped to the short isoform, which suggested that the short rather than the long version of SpoX predominantly binds *spo0A* (Fig EV1B). In summary, while the RIL‐seq data revealed that both, SpoX and SpoY interact with *spo0A*, their distinct interaction sites and chimeric read profiles suggested that the regulatory mechanisms applied by SpoX and SpoY differ.

### SpoY and SpoX directly bind the *spo0A* mRNA *in vitro* and *in vivo*

**Figure 4:**
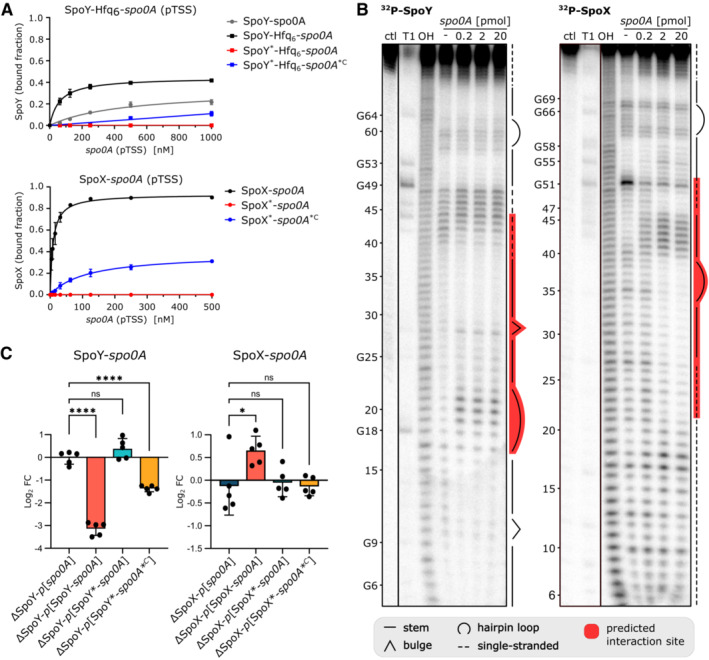
SpoY and SpoX directly interact with *spo0A in vitro* and *in vivo* AQuantification of EMSAs (error bars represent the mean ± SD of *n* = 3 biological replicates, Appendix Fig S4B and C) performed with either ^32^P‐labeled SpoY or SpoX (short isoform) with increasing concentrations of the *spo0A* target region, respectively. Purified Hfq was added to facilitate SpoY‐*spo0A* complex formation. Mutating the respective sRNA seed region (Appendix Fig S4A, SpoY*/SpoX*) abolished the interaction, while introducing compensatory mutations into the *spo0A* target region (spo0A* ^*C*^) slightly rescued the complex formation.BIn‐line probing of 0.2 pmol of ^32^P‐labeled SpoY and SpoX (short isoform) in the absence (lane 4) or presence of increasing concentrations (lane 5‐7) of the *spo0A* long 5′UTR (starting from pTSS) and first 69 nt of the CDS. RNase T1 and alkali‐digested (OH) SpoY and SpoX serve as ladders respectively. Secondary structure and predicted seed region are highlighted. A representative image of three independent experiments is shown.CmCherry fluorescence of translational fusion constructs (error bars represent the mean ± SD of *n* = 5 biological replicates, Appendix Fig S5A) expressed in the respective sRNA knockout background. Fluorescence intensity was normalized to that of the respective *p*[*spo0A*] ctl. Mutating the sRNA seed regions (Appendix Fig S4A, SpoY*/SpoX*) abolished the impact on *spo0A* translation, while introducing compensatory mutations into the *spo0A* target region (spo0A* ^*C*^) partially rescued the effect. The long SpoX isoform was used for reporter assays. Ordinary one‐way ANOVA with Dunnett′s multiple comparison test was used to calculate statistical significance. Not significant (ns) *P* > 0.05; (*) *P* ≤ 0.05; (****) *P* ≤ 0.0001. Source data are available online for this figure.

To confirm the *in silico* predicted sRNA‐*spo0A* base‐pairing, we performed electrophoretic mobility shift assays (EMSAs), combining either SpoY or SpoX with *spo0A* (5′UTR and first 69 nt of CDS, Appendix Fig S4). Considering the 5′ location of the SpoX seed region, we decided to use the short SpoX isoform for *in vitro* experiments. For *spo0A*, we initially tested two 5′UTR lengths that correspond to the previously published sigA‐dependent (long 5′UTR) and sigH‐dependent (short 5′UTR) transcription start sites of *spo0A* (Fig 3D; Saujet *et al*, 2011; Fuchs *et al*, 2021). However, since EMSAs combining SpoY or SpoX with either the long or the short *spo0A* 5′UTR showed similar results, we focused on the long 5′UTR for further experiments (Appendix Fig S4D). Complex formation of SpoY‐*spo0A* required high concentrations of *spo0A*, but clearly improved upon addition of purified Hfq (Fig 4A; Appendix Fig S4B). The complex of SpoX and *spo0A* formed more efficiently, resulting in an apparent K_D_ of 8.7 nM (Fig 4A; Appendix Fig S4C). Mutating the respective sRNA seed regions (SpoY*/SpoX*) completely abolished the interaction in both cases, while introducing compensatory mutations in the respective *spo0A* target regions (spo0A* ^*C*^) restored the complex formation, albeit not to WT levels (Fig 4A; Appendix Fig S4A–C). In‐line probing analysis further corroborated these results. As shown in Fig 4B, duplex formation of 5′‐end‐labeled SpoY with *spo0A* (long 5′UTR and first 69 nt of CDS) protected SpoY from cleavage at positions 30–41, partially confirming the predicted interaction site. Base‐pairing was even more apparent for SpoX, where a clear concentration‐dependent effect could be observed, protecting SpoX from spontaneous cleavage at positions 22–39 and 47–52 upon duplex formation with *spo0A* (Figs 4B, 3D, and EV2B). Based on these results, we conclude that SpoY and SpoX interact with *spo0A in vitro* via direct base‐pairing at distinct target sites in the *spo0A* mRNA. To further validate these interactions and their impact on *spo0A* translation *in vivo*, we designed a translational reporter system, expressing the long 5′UTR and first 20 aa of *spo0A* fused to mCherry (Appendix Fig S5A). The *spo0A* fusion construct was expressed alone (*p*[*spo0A*]) or in combination with either sRNA (*p*[SpoY‐ or SpoX‐*spo0A*]) in the respective sRNA deletion mutant. In contrast to the *in vitro* approaches described above, the long SpoX isoform was used for all reporter assays to fully reflect the *in vivo* situation, including potential regulation by nc037. Constitutive co‐expression of SpoY and the mCherry fusion construct significantly decreased fluorescence as compared to the ΔSpoY‐*p*[*spo0A*] control (ctl), demonstrating that SpoY inhibits *spo0A* translation (Fig 4C). Mutating the SpoY seed region (SpoY*) eliminated this inhibitory effect, while introducing the corresponding compensatory mutations in *spo0A* (spo0A* ^*C*^) restored the phenotype (Fig 4C, Appendix Fig S5A). Interestingly, SpoX had the opposite effect on *spo0A* translation, as co‐expression of SpoX and the mCherry fusion construct resulted in an increase in *spo0A* translation and consequently mCherry fluorescence (Fig 4C). *spo0A* translation was restored to WT levels when co‐expressing a SpoX* seed region mutant; however, introducing compensatory mutations in the *spo0A* 5′UTR did not restore the positive effect on translation. It is possible that the compensatory mutations interfere with the *spo0A* 5′UTR secondary structure, thereby preventing SpoX‐mediated opening of the *spo0A* 5′UTR to ribosome binding, as suggested above (Fig EV2B). Overall, we were able to confirm that SpoY and SpoX directly base‐pair with the *spo0A* mRNA *in vivo*, resulting in translational repression of *spo0A* by SpoY and increased translation of *spo0A* upon interaction with SpoX.

### Post‐transcriptional regulation of *spo0A* by SpoY and SpoX has opposing effects on sporulation

**Figure 5:**
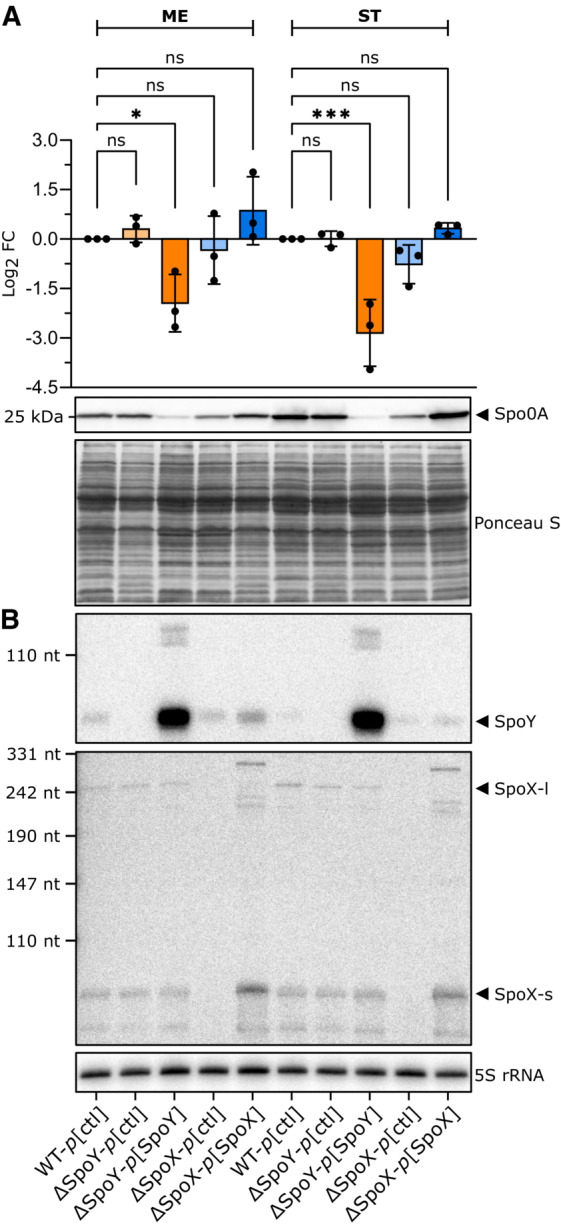
sRNA‐mediated regulation of *spo0A* affects Spo0A levels AWestern blot analysis comparing Spo0A protein levels in a WT strain (*p*[ctl]), sRNA knock‐out mutants (ΔSpoY/ΔSpoX*‐p*[ctl]) and strains constitutively expressing the respective sRNA (ΔSpoY/ΔSpoX*‐p*[ΔSpoY/ΔSpoX]). Equal optical density (OD) units of total cell lysates (error bars represent the mean ± SD of *n* = 3 biological replicates) were loaded from strains gown till mid‐exponential (ME) or stationary (ST) phase of growth in TY. Band intensities were measured and Log_2_ fold changes were calculated relative to the respective WT. Western blot membranes were incubated with anti‐Spo0A antibody. To calculate statistical significance, an ordinary one‐way ANOVA with Dunnett′s multiple comparison test was applied. Not significant (ns) *P* > 0.05; (*) *P* ≤ 0.05; (***) *P* ≤ 0.001. For western blot analysis Ponceau S staining of the blotting membrane served as loading control and for northern blot analysis 5S rRNA.BNorthern blot analysis validating sRNA expression in the respective growth conditions. Source data are available online for this figure.

**Figure 6:**
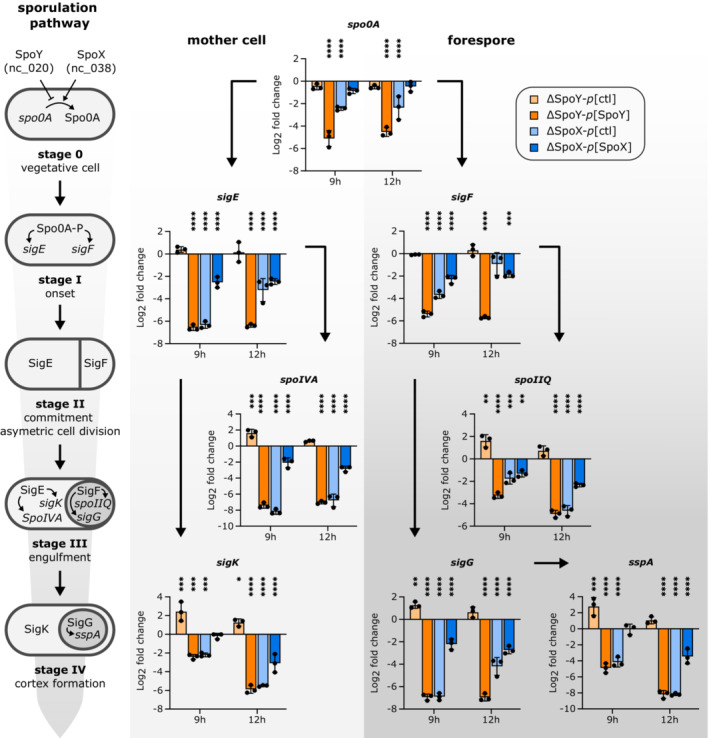
sRNA mediated post‐transcriptional regulation of *spo0A* affects sporulation‐specific gene expression Transcript levels of genes encoding sporulation‐specific sigma factors and their respective regulon in sRNA knock‐out mutants (ΔSpoY/ΔSpoX‐*p*[ctl]) and strains constitutively expressing the respective sRNA (ΔSpoY/ΔSpoX‐*p*[ΔSpoY/ΔSpoX]) relative to *C. difficile* 630 WT (*p*[ctl]). A schematic representation of the first four stages of sporulation in *C. difficile* is given on the left. RNA was extracted from samples (*n* = 3 biological replicates) taken at 9‐ and 12‐h post induction of sporulation on 70:30 sporulation plates. Log_2_ fold changes were calculated relative to the WT. Statistical significance was determined using two‐way ANOVA with Dunnett′s multiple comparison test. *P* > 0.05; (*) *P* ≤ 0.05; (**) *P* ≤ 0.01; (***) *P* ≤ 0.001; (****) *P* ≤ 0.0001. Error bars represent the mean ± SD. Validation of SpoX and SpoY expression for the respective strains and time points by northern blot analysis is shown in Appendix Fig S6A. Source data are available online for this figure.

Considering the evident alteration of *spo0A* translation, we hypothesized that SpoY and SpoX impact native Spo0A protein levels *in vivo*. Accordingly, we performed Western blot analysis to compare Spo0A protein levels in a WT strain (*p*[ctl]) to those in the SpoY and SpoX deletion mutants (e.g., ΔSpoY‐*p*[ctl]), or to strains constitutively overexpressing the respective sRNA (e.g., ΔSpoY‐*p*[SpoY]). Although there was no effect on Spo0A in a SpoY deletion mutant, overexpression of SpoY resulted in a significant decrease in Spo0A protein levels (∼2.5‐fold, Fig 5A), confirming that SpoY inhibits *spo0A* translation. In contrast, deleting SpoX slightly decreased Spo0A levels, while overexpression of SpoX restored the Spo0A signal to WT levels. Coresponding sRNA expression was confirmed via NB analysis (Fig 5B). These results corroborated our model, in which SpoX positively impacts Spo0A translation by base‐pairing to the *spo0A* 5′UTR. Although SpoX and SpoY regulate *spo0A* translation, changes in Spo0A levels might not directly translate into changes in Spo0A activity, as Spo0A requires additional activation via phosphorylation (Spo0A‐P; Edwards & McBride, 2014). To evaluate whether SpoX‐ and SpoY‐mediated changes in Spo0A levels correlated with Spo0A‐P activity, we analyzed transcript levels of several sporulation‐specific genes that operate downstream of Spo0A‐P (Fig 6). Specifically, we measured transcript levels of *sigE*, *sigF*, *spoIV* (σ^E^ regulon), *spoIIQ* (σ^F^ regulon), *sigK*, *sigG*, and *sspA* (σ^G^ regulon) as well as *spo0A* via qRT‐PCR (Fimlaid *et al*, 2013; Saujet *et al*, 2014). In accordance with our previous results, SpoY overexpression had a negative effect on transcript levels of all tested genes, whereas SpoY deletion had either no effect (*sigE*, *sigF*) or resulted in an increase in transcript abundance. In contrast, deletion of SpoX reduced transcript levels of all tested genes, while SpoX overexpression partially restored transcript abundance to WT levels. Taken together, the observed changes in expression of sporulation‐specific genes suggested that modulation of Spo0A levels by SpoY leads to an overall downregulation of the sporulation cascade, while activity of SpoX results in upregulation of spore formation. Interestingly, SpoY overexpression and SpoX deletion also resulted in a decrease in *spo0A* transcript levels, potentially by affecting ribosome occupancy of the *spo0A* mRNA (Fig 6). By altering ribosome occupancy of mRNA targets, sRNA‐mediated regulation can indirectly affect RNase‐mediated cleavage and thus mRNA stability (Deana & Belasco, 2005; Prevost *et al*, 2011).

**Figure 7:**
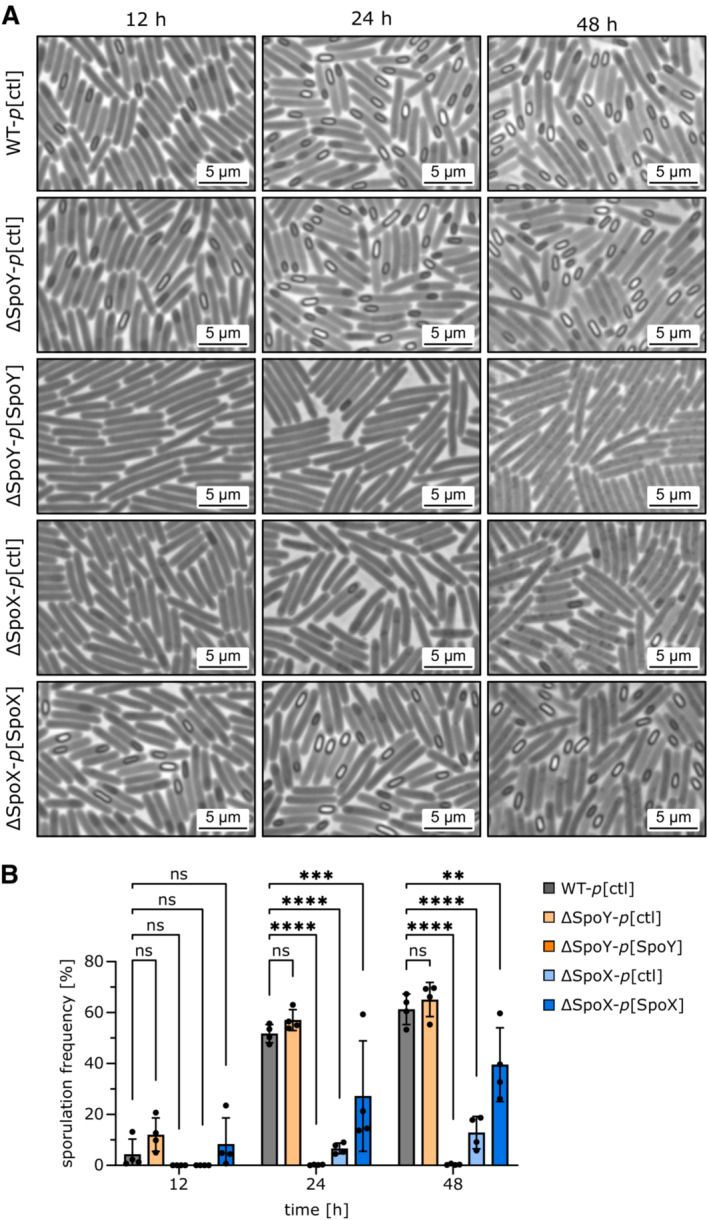
sRNA‐mediated regulation of *spo0A* affects sporulation frequencies ARepresentative phase‐contrast images (*n* = 4) of a WT strain (*p*[ctl]), sRNA knockout mutants (ΔSpoY/ΔSpoX*‐p*[ctl]) and strains constitutively expressing the respective sRNA (ΔSpoY/ΔSpoX*‐p*[ΔSpoY/ΔSpoX]) at 6‐, 12‐, 24‐, and 48‐h post inoculation of 70:30 liquid sporulation medium.BSporulation frequencies calculated based on phase‐contrast microscopy. Error bars represent the mean ± SD of *n* = 4 biological replicates (A). Two‐way ANOVA with Dunnett′s multiple comparison test was used to calculate statistical significance. Not significant (ns) *P* > 0.05; (**) *P* ≤ 0.01; (***) *P* ≤ 0.001; (****) *P* ≤ 0.0001. Source data are available online for this figure.

To further assess the impact of SpoY and SpoX on sporulation, we determined sporulation frequencies of WT and mutant strains by phase contrast microscopy. As shown in Fig 7A and B, SpoY overexpression and SpoX deletion resulted in significantly reduced sporulation frequencies during late time points (12 and 24 h). These observations were confirmed by CFU‐based calculation of sporulation frequencies (Appendix Fig S6B). Hence, our data show that SpoY and SpoX not only affect *spo0A* translation in an inverse manner, but consequently influence gene expression of sporulation‐specific genes and ultimately sporulation frequencies.

### SpoY and SpoX impact *C. difficile* gut colonization in a mouse model of *C. difficile* infection

**Figure 8:**
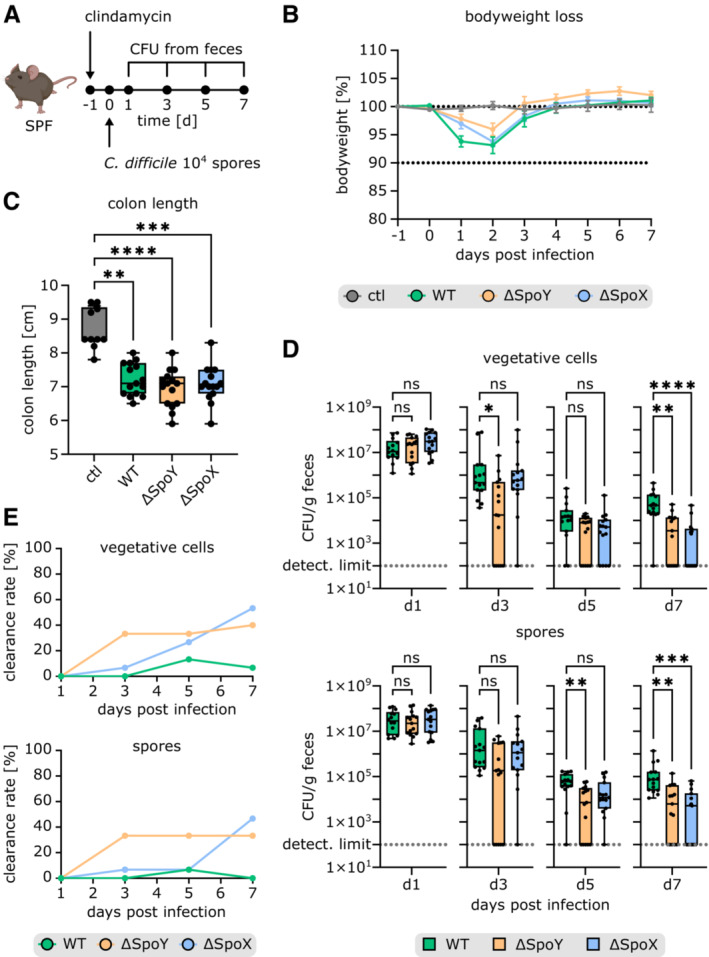
SpoY and SpoX deletion affects *C. difficile* gut colonization and spore burden in a mouse model of *C. difficile* infection ASchematic representation of the mouse model of *C. difficile* infection (partially created with BioRender.com). SPF mice were treated with clindamycin 24 h prior to infection, administered via intraperitoneal injection to induce susceptibility to *C. difficile* infection (Theriot *et al*, 2016). Following antibiotic treatment, groups of mice were infected with 10^4^ spores of *C. difficile* 630 WT (*n* = 15), ΔSpoY (*n* = 15), or ΔSpoX (*n* = 15) via oral gavage. Mice were monitored for disease between Days 0 and 7 post infection. Fecal samples were collected at indicated time points to determine pathogen burden.B, C(B) Body weight loss over the course of 7 days (data points and error bars represent mean ± standard error of the mean; the dotted lines serve as a visual guideline) as well as (C) final colon length at Day 7 post infection of uninfected mice (*n* = 10) and mice infected with *C. difficile* 630 WT (*n* = 15), ΔSpoY (*n* = 15), or ΔSpoX (*n* = 15) are indicated (box‐and‐whisker plots show minimum, first quartile, median, third quartile, and maximum).DComparison of CFUs of *C. difficile* vegetative cells and spores in fecal pellets of mice at different time points during infection with *C. difficile* 630 WT (*n* = 15), ΔSpoY (*n* = 15), or ΔSpoX (*n* = 15). Replicates with CFUs below the detection limit were set to 100. Box‐and‐whisker plots show minimum, first quartile, median, third quartile, and maximum.EClearance rate (number of mice below detection limit divided by the total number of mice) of SPF mice infected with *C. difficile* 630 WT (*n* = 15), ΔSpoY (*n* = 15), or ΔSpoX (*n* = 15). Data information: Kruskal–Wallis was performed with Dunnett′s multiple comparison test to calculate statistical significance in (C, D). Not significant (ns) *P* > 0.05; (*) *P* ≤ 0.05; (**) *P* ≤ 0.01; (***) *P* ≤ 0.001; (****) *P* ≤ 0.0001. Source data are available online for this figure.

Considering the marked impact of SpoY and SpoX deletion on sporulation in *C. difficile*, as well as their extended interactome, we decided to monitor the effect of SpoY and SpoX deletion in a mouse model of *C. difficile* infection (Fig 8A). Overall, a delayed onset of disease in mice challenged with the sRNA deletion strains was observed, as ΔSpoY and ΔSpoX infected mice showed a delayed body weight loss compared with mice infected with *C. difficile* WT (Fig 8B). Nevertheless, colon shortening on Day 7 was equally severe in mice challenged with *C. difficile* WT, ΔSpoY, or ΔSpoX suggesting similar levels of toxin production and consequently disease severity over the course of infection (Fig 8C). Initial colonization was comparable in ΔSpoY, ΔSpoX, and WT‐treated mice, as no difference in vegetative cells or spores was observed on Day 1 post infection (Fig 8D). However, lower CFUs of spores and vegetative cells were recovered from feces of ΔSpoY and ΔSpoX infected mice at Days 3, 5, and 7 post infection, compared with mice challenged with *C. difficile* WT. Accordingly, mice infected with ΔSpoY or ΔSpoX strains exhibited an accelerated *C. difficile* clearance rate (Fig 8E). This was particularly evident in ΔSpoY‐treated mice starting from Day 3, while the bacterial burden in mice infected with ΔSpoX only decreased on Day 5 (vegetative cells) and Day 7 (vegetative cells and spores) compared with WT‐treated mice. Generally, the lower spore counts in ΔSpoY‐ or ΔSpoX‐infected mice were paralleled by lower vegetative cell counts, revealing a global effect of SpoY and SpoX deletion on *C. difficile* gut colonization rather than on sporulation alone (Fig 8D). In support of this hypothesis, our RIL‐seq dataset suggested base‐pairing of SpoY with the *cwp2* mRNA as well as base‐pairing of SpoX with the *cwpV* mRNA (Dataset EV2), each encoding cell wall proteins with predicted virulence functions (Reynolds *et al*, 2011; Bradshaw *et al*, 2017). Using our translational reporter system, we were able to validate these predicted RIL‐seq targets. Quantification of fluorescence signals of the respective mCherry fusion constructs revealed that SpoY inhibits *cwp2* translation, while SpoX acts as a positive regulator of *cwpV* translation (Fig EV4A and B). Taken together, the impact of SpoY and SpoX on intestinal pathogenesis suggested that their regulatory functions extend beyond regulating Spo0A protein levels and likely include additional regulatory targets that contribute to intestinal colonization.

## Discussion

A plethora of research in human pathogens such as *Pseudomonas aeruginosa*, *S. enterica*, and *Vibrio cholerae* has highlighted the importance of sRNAs in regulating virulence pathways (Westermann, 2019). To uncover sRNA‐mediated regulatory mechanisms that might shape *C. difficile* virulence, we performed RIL‐seq during the onset of sporulation in *C. difficile* (Saujet *et al*, 2011 *)*. Endospore formation has been extensively studied, particularly in the model organism *B. subtilis* and is a tightly regulated process, defined by several sequential morphological stages (Fig 6; Errington, 2003). Although the sporulation cascade is generally conserved between *C. difficile* and other endospore‐forming Firmicutes, there are some striking differences, most notably regarding sporulation initiation (Shen *et al*, 2019). In *B. subtilis*, environmental signals that induce sporulation are channeled through a complex phosphorelay system consisting of several sensor kinases and phosphotransferases, culminating in the activation of Spo0A (Stephenson & Hoch, 2002). Phosphorylated Spo0A‐P then initiates the sporulation process by activating the transcription of several key sporulation‐specific genes (Stephenson & Hoch, 2002). Unlike *B. subtilis*, *C. difficile* does not encode an apparent intermediate phosphorelay system (Edwards & McBride, 2014). Although three putative sensor histidine kinases (PtpA‐C) have been described to directly influence Spo0A phosphorylation, the overall process of Spo0A activation remains barely understood and points toward additional unknown mechanisms regulating Spo0A activity in *C. difficile* (Edwards & McBride, 2014; Childress *et al*, 2016; Edwards *et al*, 2022).

**Figure 9:**
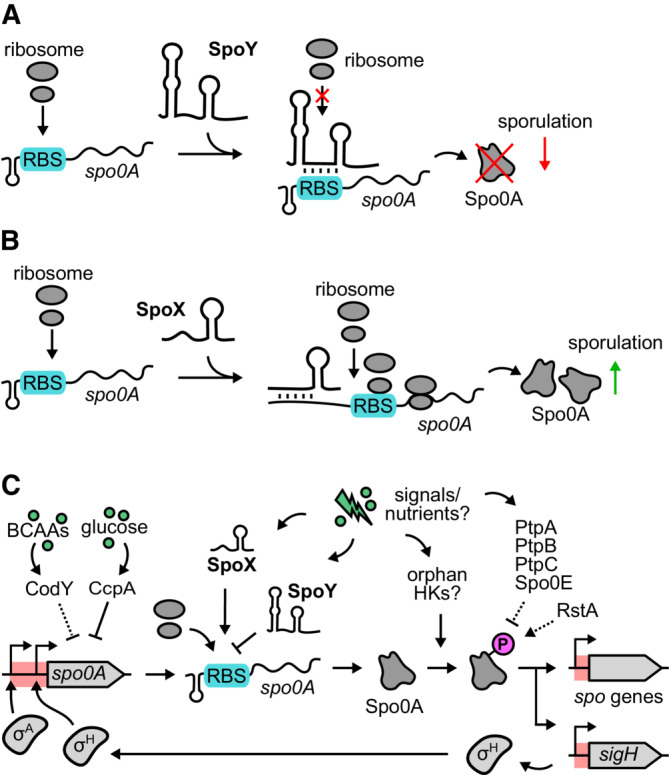
SpoY and SpoX add a post‐transcriptional layer to the complex regulatory network shaping sporulation initiation in *C. difficile* A, BModel of the SpoY‐ and SpoX‐mediated regulation of *spo0A* translation. SpoY base‐pairing with the *spo0A* RBS (highlighted in blue) inhibits ribosome binding, ultimately resulting in translational repression of *spo0A* and decreased sporulation. In contrast, SpoX base‐pairing with the *spo0A* 5′UTR most likely results in a conformational change of the *spo0A* 5′UTR that renders the RBS more accessible, ultimately leading to increased translation and sporulation.CCurrent model of transcriptional, post‐transcriptional, and post‐translational regulators affecting initiation of sporulation in *C. difficile* (Lee *et al*, 2022). Arrows indicate positive regulation and blunt ends negative regulation. Dotted lines indicate an indirect regulation or regulation where the mechanism of action is still unknown, whereas solid lines indicate verified direct interactions. Red shades correspond to untranslated regions. Phosphorylated Spo0A is highlighted by a pink “P”. Branched‐chain amino acids are abbreviated as “BCAAs” and histidine kinases as “HKs.”

In this study, we identified sRNA‐mediated post‐transcriptional regulation of *spo0A* translation as a new mechanism contributing to sporulation initiation in *C. difficile* (Fig 9A–C). There are a few examples of sRNA‐mediated regulation of sporulation in endospore‐forming Firmicutes. In *B. subtilis*, sRNA SR1 inhibits translation of the histidine kinases *kinA* that transmits environmental signals, eventually resulting in phosphorylation of Spo0A (Ul Haq *et al*, 2021). Another example is the *virX* sRNA in *C. perfringens that* negatively regulates sporulation by repressing transcription of the early forespore‐specific σ factor σ^F^ (Ohtani *et al*, 2013). Furthermore, sRNA RCd1 in *C. difficile* inhibits production of the late mother cell‐specific σ factor σ^K^ by preventing the excision of the prophage‐like element that interrupts the *sigK* gene (Boudry *et al*, 2021). Here, we characterized two novel sRNAs, SpoY and SpoX, that function by directly binding and regulating the *spo0A* mRNA (Figs 4 and 3D). We could show that SpoY operates by base‐pairing with the *spo0A* RBS region, thereby inhibiting *spo0A* translation, resulting in reduced sporulation frequencies when overexpressed (Figs 7 and 9A). In contrast, SpoX interaction with the *spo0A* 5′UTR results in an upregulation of *spo0A* translation and consequently sporulation, most likely through a change of the *spo0A* 5′UTR secondary structure upon base‐pairing with SpoX (Fig 9B). Analogous mechanisms have been described in the literature; a well‐known example is the positive regulation of *rpoS*, the key regulator of general stress responses in *E. coli* (Kim & Lee, 2020 *)*. In this case, several sRNAs (DsrA, RprA, and ArcZ) base‐pair with the *rpoS* 5′ leader to expose the *rpoS* translational start site that is otherwise blocked by an inhibitory stem‐loop structure (Kim & Lee, 2020).

**Figure EV3:**
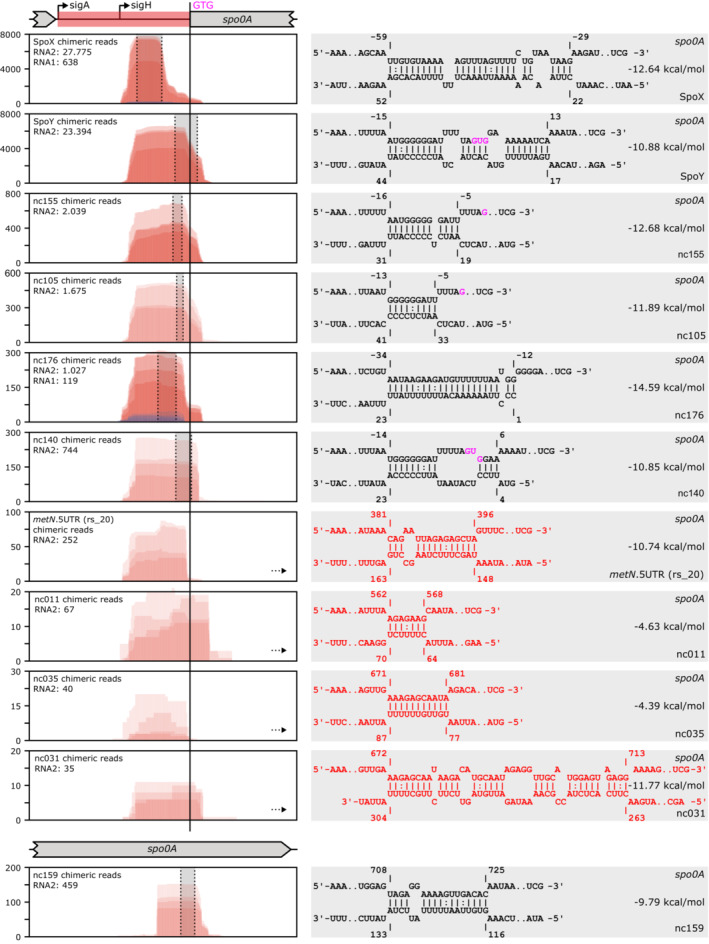
*spo0A* is a target of extensive sRNA‐mediated post‐transcriptional regulation On the left site, read coverage (y‐axis) of *spo0A* by chimeric reads of all sRNA‐*spo0A* interactions detected by RIL‐seq analysis is depicted. The *spo0A* 5′UTR position including pTSS (sigA) and sTSS (sigH), start codon and coding sequence are marked on the x‐axis. Chimeric reads covering *spo0A* were predominantly found at position 1 (RNA1) in a chimera and are color‐coded in red. Chimeric reads found at position 2 (RNA2) are marked in blue. The number of chimeric reads covering each interaction is provided on the left. On the right site, base pairing information and location of the predicted binding sites (IntaRNA; Mann *et al*, 2017) for each interaction are highlighted. *In silico* predictions that do not overlap with RIL‐seq data are marked in red, and location of the interaction in relation to the RIL‐seq peak is indicated by an arrow. Predicted interactions overlapping with RIL‐seq data are shaded in gray and superimposed over the coverage plots. The *spo0A* nucleotide position is calculated relative to the *spo0A* start codon (highlighted in pink).

Of note, chimeras formed by *spo0A* and SpoX or SpoY are not the only *spo0A* interactions present in the dataset, albeit the most enriched. In fact, we found nine additional chimeras consisting of the *spo0A* mRNA ligated to an sRNA (*n* = 8) or another mRNA (*n* = 1, Fig EV3). Of these nine interactions, eight displayed a chimeric read profile comparable with SpoY, suggesting a similar mode of action. Accordingly, these sRNAs might substitute for the SpoY function, potentially explaining the minor sporulation phenotype of a SpoY deletion strain. Interestingly, *in silico* predicted interaction sites overlap with the RIL‐seq peak profile for five of the detected *spo0A*‐sRNA interactions, further corroborating the RIL‐seq results (Fig EV3; Mann *et al*, 2017). There are known examples of mRNAs that are directly targeted by multiple sRNAs, most of which encode key regulators. For instance, *flhDC*, the master regulator of flagellar genes in *E. coli* interacts with five sRNAs (ArcZ, OmrA, OmrB, OxyS, and McaS) that base‐pair with the *flhDC* 5′UTR resulting in either negative or positive regulation of motility (De Lay & Gottesman, 2012). Similarly, biofilm formation is a central target of sRNA‐mediated regulation through base‐pairing of seven sRNAs (OmrA/B, McaS, RprA, RydC, GcvB, and RybB) with the *csgD* 5′UTR, encoding a central regulator of curli formation in *E. coli* (Mika & Hengge, 2014). sRNA transcription in turn is regulated in response to specific environmental or nutritional signals, allowing targeted mRNA regulation in relevant conditions. For example, OmrA/B are regulated by the EnvZ/OmpR two‐component system in response to high osmolarity while McaS is activated by cAMP‐CRP in response to carbon limitation (Guillier & Gottesman, 2006; Thomason *et al*, 2012).

In line with these examples, *C. difficile* Spo0A might constitute another key regulator that is a central target of sRNA‐mediated regulation. Accordingly, the various sRNAs interacting with *spo0A* may serve to integrate different environmental signals to modulate Spo0A expression, thus partially replacing the missing phosphorelay system that translates environmental cues into Spo0A activity in *B. subtilis* (Stephenson & Hoch, 2002; Edwards & McBride, 2014). This hypothesis is further corroborated by the distinct expression profiles of SpoY and SpoX, which points to *spo0A* regulation during different growth phases (Fig 3C). SpoY most likely suppresses *spo0A* translation during early growth stages, in conditions that favor active growth rather than sporulation. In contrast, SpoX expression indicates that it exerts its positive effect on *spo0A* translation mostly in late exponential and early stationary phase when the sporulation process initiates. Given the asynchronous nature of sporulation initiation in *C. difficile*, identifying the specific environmental cues and transcription factors that regulate expression of both sRNAs and thus *spo0A* will likely necessitate single‐cell‐based approaches to fully understand the nuanced post‐transcriptional regulation of *spo0A*. Accordingly, methods such as scRNA‐seq could be applied to dissect the various physiological cellular states that coexist within a population and often mask distinct regulatory events in individual cells that undergo sporulation.

Interestingly, some of the sRNAs interacting with *spo0A* also formed chimeras with SpoX, including SpoY, nc105, and nc176 (Fig EV1A). It is possible that SpoX not only upregulates *spo0A* translation, but also sponges sRNAs that would otherwise inhibit *spo0A* translation. This could explain why the deletion of SpoX has such a pronounced effect on sporulation frequencies (Fig 7). Alternatively, it is equally likely that SpoY, nc105, and nc176 sRNA regulate SpoX activity, preventing positive regulation of sporulation in conditions favoring active proliferation. sRNA sponges generally act by sequestering a target sRNA, thereby preventing the sRNA‐target interaction, an effect that depends on the stoichiometry between the sponge, sRNA, and mRNA (Denham, 2020). Reports of sRNAs simultaneously acting as sRNA sponge and mRNA regulator have been published previously, supporting this hypothesis (Denham, 2020). For example, ArcZ and CyaR, both known regulators of the *rpoS* mRNA, also interact with each other, as ArcZ overexpression reduces CyaR steady‐state levels and upregulates CyaR targets (Iosub *et al*, 2020). Further research is necessary to verify these SpoX‐sRNA interactions and decipher if, when, and how these interactions impact Spo0A activity (Denham, 2020).

**Figure EV4:**
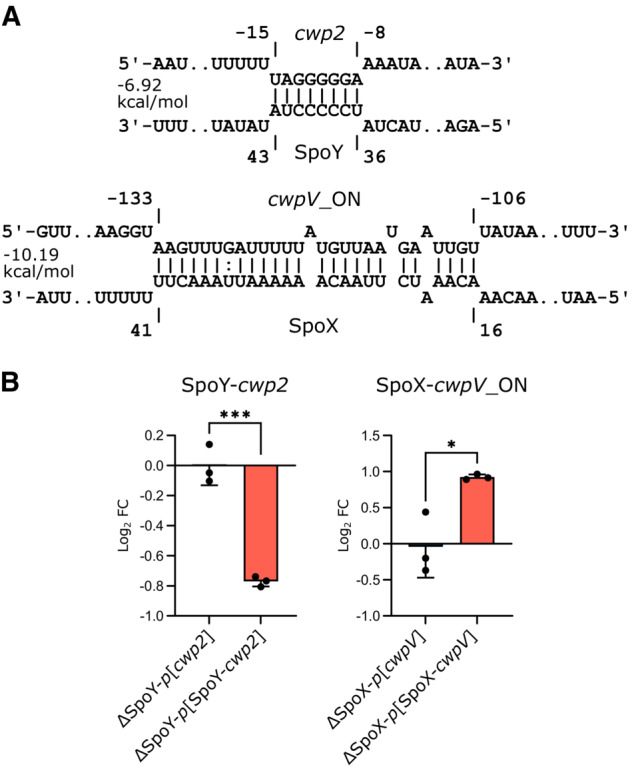
SpoY and SpoX target additional genes besides *spo0A* ABase pairing information and location of the predicted binding sites (IntaRNA; Mann *et al*, 2017) for SpoY‐*cwp2* and SpoX‐*cwpV* with the invertible region within the *cwpV* 5′UTR in the ON orientation, allowing CwpV expression (Emerson *et al*, 2009). The nucleotide positions for *cwpV* and *cwp2* are calculated relative to the respective start codons.BmCherry fluorescence of translational fusion constructs (error bars represent the mean ± SD of *n* = 3 biological replicates, Appendix Fig S5A) expressed in the respective sRNA knockout background. Fluorescence intensity was normalized to that of the respective *p*[*cwp2*]/ *p*[*cwpV*] ctl. Unpaired *t*‐test was used to calculate statistical significance. Not significant (ns) *P* > 0.05; (*) *P* ≤ 0.05; (***) *P* ≤ 0.001.

As discussed above, our study indicates a complex network of post‐transcriptional regulatory interactions that shape *C. difficile* virulence. Not surprisingly, our mouse experiments have revealed a global impact of SpoX and SpoY on *C. difficile* gut colonization. Interestingly, this impact appears to be in contrast to the opposing effects on sporulation, which we observed under isolated *in vitro* conditions that lack complex gut interactions. Of note, Spo0A has been implicated in pathways other than sporulation, as Spo0A inactivation also results in altered toxin production and biofilm formation in *C. difficile* (Underwood *et al*, 2009; Dawson *et al*, 2012; Pettit *et al*, 2014). Hence, sRNA‐mediated regulation of *spo0A* might impact additional processes besides sporulation that could not be covered in this work and will require further investigation (Underwood *et al*, 2009; Dawson *et al*, 2012; Taggart *et al*, 2021). Moreover, it is important to consider that both sRNAs interact with additional targets (Fig EV1A). For example, SpoY also inhibits translation of *cwp2*, a cell wall protein known to affect cellular adherence *in vitro* (Bradshaw *et al*, 2017). Furthermore, SpoX positively regulates translation of *cwpV*, which encodes a cell wall protein described to promote cell aggregation (Fig EV4; Reynolds *et al*, 2011). Consequently, deleting or overexpressing either sRNA *in vivo* most likely affects processes beyond sporulation and might explain the global effect of both sRNA deletion strains in the mouse model of *C. difficile* infection (Fig 8).

In summary, the application of Hfq RIL‐seq to *C. difficile* has revealed a global view of extensive Hfq‐mediated RNA interactions (Fig 1B; Dataset EV2; Park *et al*, 2021). Although we have barely scratched the surface of sRNA‐mediated regulation in *C. difficile*, our RIL‐seq data represent a starting point for the characterization of additional processes modulated by sRNAs. In this work, we uncovered a new layer of post‐transcriptional regulation in *C. difficile* hinting at a complex sRNA network regulating sporulation in this important human pathogen. Given the low conservation of mechanisms governing sporulation initiation, these results might open an interesting avenue for potential therapeutic targets to counteract CDI. In fact, the use of antisense nucleic acids to selectively target species in a microbial community has gained attention as a promising alternative to conventional antibiotics (Mondhe *et al*, 2014; Sully & Geller, 2016). Accordingly, mimicking or blocking the activity of sRNAs using antisense nucleic acid derivatives might represent an interesting alternative, especially considering the contribution of antibiotics to CDI recurrence (Zhu *et al*, 2018; Westermann, 2019).

## Materials and Methods

### Bacterial strains and growth conditions

A complete list of all *C. difficil*e and *E. coli* strains used in this study is provided in Appendix Table S1. *C. difficile* 630 cultures were routinely grown anaerobically inside a Coy chamber (85% N_2_, 10% H_2_ and 5% CO_2_) in Brain Heart Infusion (BHI) broth or on BHI agar plates (1.5% agar) unless stated otherwise. If necessary, antibiotics were added to the medium at the following concentrations: thiamphenicol (TAP) 15 μg/ml, cycloserine (CS) 250 μg/ml, cefoxitin (FOX) 8 μg/ml. *E. coli* cultures were propagated aerobically in Luria‐Bertani (LB) broth (10 g/l tryptone, 5 g/l yeast extract, 10 g/l NaCl) or on LB agar plates (1.5% agar) supplemented with chloramphenicol (CHL, 20 μg/ml). *E. coli* strain Top10 (Invitrogen) served as a recipient for all cloning procedures, and *E. coli* CA434 (HB101 carrying the IncPβ conjugative plasmid R702) was used as donor strain for plasmid conjugations into *C. difficile* 630.

### Plasmid construction

All plasmids and DNA oligonucleotides used in this study are listed in Appendix Tables S2 and S3, respectively. *E. coli* TOP10 was used for plasmid propagation according to the standard procedures (Green & Sambrook, 2012).

#### pFF‐53—plasmid for generating a *hfq*::3 × FLAG strain

For insertion of a C‐terminal *hfq::*3 × FLAG‐tag, allelic exchange cassettes were designed with approximately 1.2 kb of homology to the chromosomal sequence flanking the up‐ and downstream regions of the *hfq* stop codon. Both homology regions were PCR‐amplified from *C. difficile* 630 using high‐fidelity Fusion Polymerase (Mobidiag) with 5% DMSO, FFO‐364/−365 and FFO‐368/−369. The resulting fragments were gel‐purified with NucleoSpin Gel and PCR Clean‐Up Kit (Macherey‐Nagel). The 3 × FLAG‐tag (DYKDHDGDYKDHDIDYKDDDDK) was similarly amplified and purified with FFO‐366/−367, using the previously published pFF‐12 as a template (Fuchs *et al*, 2021). Insert assembly and ligation into PCR‐linearized pJAK184 (FFO‐362/−363) was achieved via Gibson Assembly (Gibson Assembly® Master Mix, New England BioLabs) according to the manufacturer's instructions, resulting in pFF‐53 (Fuchs *et al*, 2021).

#### pFF‐162/‐163/‐164/‐245/‐166/‐248/‐247/‐167—plasmids for *in vitro* transcriptions

SpoY (FFO‐958/‐959), SpoY* (FFO‐958/‐960), SpoX (short isoform, FFO‐961/‐962), SpoX* (short isoform, FFO‐1261/‐1262), *spo0A* 5′UTR starting from pTSS and first 69 nt of coding region (FFO‐964/‐966) and *spo0A* 5′UTR starting from sTSS and first 69 nt of coding region (FFO‐965/‐966) were PCR‐amplified from *C. difficile* 630 using Fusion Polymerase (Mobidiag), adding a 5′ overhang comprising the T7‐promoter sequence (5′‐GTTTTTTTTAATACGACTCACTATAGGG). For inserting SpoY* compensatory mutations in *spo0A*, spo0A* ^*C*^ was amplified in two parts using FFO‐964/‐1268 and FFO‐1269/‐966, before joining both fragments via SOEing PCR with FFO‐964/‐966. SpoX* compensatory mutations were inserted similarly using FFO‐964/‐1259 and FFO‐1267/‐966, followed by SOEing PCR with FFO‐964/‐966. In each case, PCR products were gel‐purified as described above. Subsequently, 3′‐adenine overhangs were added to all PCR product using Taq Polymerase (Biozym). The resulting fragments were cloned into the StrataClone TA‐cloning vector and transformed into StrataClone SoloPack competent cells according to the manufacturer's protocol (StrataClone PCR Cloning Kit, Agilent), resulting in pFF‐162 (SpoY), pFF‐163 (SpoY*), pFF‐164 (SpoX), pFF‐245 (SpoX*), pFF‐166 (*spo0A* with 5′UTR starting from pTSS), pFF‐248 (spo0A* ^*C*^ SpoY*), pFF‐247 (spo0A* ^*C*^ SpoX*) and pFF‐167 (*spo0A* with 5′UTR starting from sTSS).

#### pFF‐170/‐171—Plasmids for generating SpoY and SpoX deletion mutants

For deletion of SpoY and SpoX, allelic exchange cassettes were designed with approximately 1.2 kb of homology to the chromosomal sequence flanking the deletion sites of SpoY and SpoX. To avoid polar effects on genes or sRNAs encoded adjacent to SpoY or antisense to SpoX (Fig 3B), the deleted region was restricted to nucleotide 11–46 in case of SpoY, and nucleotide 1–83 of SpoX in addition to 40 nt upstream of SpoX encompassing the SpoX promoter region. Homology arms were PCR‐amplified, and gel‐purified as described above, using FFO‐977/‐978 and FFO‐979/‐980 for the SpoY homology arms, and FFO‐985/‐986 and FFO‐987/‐988 for amplification of the SpoX homology regions, respectively. The homology arms were joined via SOEing PCR resulting in one large fragment encompassing both homology regions, and a BamHI/SacI restriction site at the 5′/3′ end for both, SpoY (FFO‐977/‐980) and SpoX (FFO‐985/‐988). Following restriction digest using BamHI and SacI, the fragments were mixed in a 3:1 ratio with an equally digested and gel‐purified pJAK112 and ligated overnight at 4°C using T4 DNA ligase (Thermo Scientific), resulting in pFF‐170 (SpoY deletion) and pFF‐171 (SpoX deletion; Fuchs *et al*, 2021).

#### pFF‐185/‐186/‐191/‐254/‐285/‐187/‐192/‐260/‐289/‐207/‐344/‐345/‐346/‐347—translational fusion reporter

To discern the impact of sRNA‐target interactions on target translation, we designed a translational fusion system based on the previously published pDSW1728 mCherryOpt plasmid, which was initially designed to study gene expression (Ransom *et al*, 2015). The full reporter system (RS) constitutively co‐expresses an sRNA controlled by an *fdxA* promoter and the target 5′UTR fused to mCherryOpt, controlled by a *cwp2* promoter. Regulation of target translation via sRNAs is measured by comparing fluorescence of the strain expressing the full RS (*p*[sRNA‐*target*]) to a control strain only expressing the sRNA (*p*[sRNA]) or the target 5′UTR fused to mCherryOpt (*p*[*target*]). All plasmids were designed to allow easy exchange of individual components via restriction digestion and are illustrated in Appendix Fig S5A. sRNA expression was verified via northern blot analysis (Appendix Fig S5B and C). For generating a *p*[*spo0A*] plasmid, the *cwp2* promoter and the *spo0A* 5′UTR (starting from the pTSS and including 60 nt of CDS) were PCR‐amplified and gel‐purified as described above from *C. difficile* 630. FFO‐1004/‐1000 and FFO‐1001/‐1002 were used, respectively, thereby adding NheI/XhoI restriction at the 5′/3′ end of the *cwp2* promoter, and a XhoI/SacI restriction site at the 5′/3′ end of the *spo0A* 5′UTR, respectively. mCherryOpt was PCR‐amplified from pDSW1728 with FFO‐1056/‐1057 starting with the second codon of the mCherryOpt CDS, adding a SacI restriction site directly upstream and preserving the BamHI restriction site at the 3′ end. All components were subjected to restriction digest using the appropriate restriction enzymes and ligated into the NheI/BamHI digested pDSW1728 vector as described above, resulting in pFF‐185 (*p*[*spo0A*]). To generate a *p*[sRNA] plasmid, the *fdxA* promoter and SpoY were PCR‐amplified from *C. difficile* 630 with FFO‐995/‐1005 and FFO‐1006/‐1007, respectively, inserting an NheI restriction site at the *fdxA* 5′ end and a SpoY overlapping region at the 3′ end. Inserting a restriction site upstream of SpoY was avoided to preserve the sRNA primary and secondary structure. Accordingly, an *fdxA* overlapping region was added to the SpoY 5′ end and an XbaI restriction site at the 3′ end followed by the *slpA* terminator and a BamHI restriction site to prevent readthrough. Both fragments were joined via SOEing PCR with FFO‐995/‐1007, NheI/BamHI digested and ligated into the NheI/BamHI digested pDSW1728 vector, resulting in pFF‐186 (*p*[SpoY]). Finally, the *p*[sRNA‐*target*] plasmid was generated by PCR amplifying the *cwp2*‐*spo0A* 5′UTR‐mCherryOpt construct from pFF‐185, using FFO‐999 and FFO‐1057, thereby exchanging the 5′ NheI with an XbaI restriction site followed by the *slpA* terminator. The resulting fragment was digested with XbaI/BamHI and ligated into the equally digested pFF‐186 yielding pFF‐191 (*p*[SpoY‐*spo0A*]). All remaining plasmids were generated by exchanging either sRNA or target of the plasmids described above. For SpoX constructs, *fdxA* and SpoX were PCR‐amplified using FFO‐995/‐1008 and FFO‐1009/‐1010. Both fragments were joined via SOEing PCR with FFO‐995/‐1010, digested with NheI/XbaI and ligated into NheI/XbaI digested pFF‐186 and pFF‐191, resulting in pFF‐187 (*p*[SpoX]) and pFF‐192 (*p*[SpoX‐*spo0A*]). For SpoY* constructs, SpoY* was PCR‐amplified from pFF‐163 with FFO‐1006/‐1007 and joined via SOEing PCR with the previously amplified *fdxA* promoter, using FFO‐995/‐1007. The SOEing product was NheI/XbaI digested and ligated into the equally digested pFF‐191, yielding pFF‐254 (*p*[SpoY*‐*spo0A*]). Next, *spo0A* harboring compensatory mutations was PCR‐amplified from pFF‐248, using FFO‐1001/‐1002, digested with XhoI/SacI, and ligated into XhoI/SacI digested pFF‐254 resulting in pFF‐285 (*p*[SpoY*‐spo0A* ^*C*^]). Generation of SpoX* constructs was achieved by first amplifying SpoX* in two fragments, inserting the seed region mutations with FFO‐1264/‐1262 and FFO‐1263/‐1010. Both fragments were then joined via SOEing PCR with FFO‐1264/‐1010, followed by a second SOEing PCR, combining the previously amplified *fdxA* promoter and the full length SpoX*, using FFO‐995/‐1010. The resulting product was NheI/XbaI digested and ligated into a similarly digested pFF‐191, yielding pFF‐260 (*p*[SpoX*‐*spo0A*]). Finally, *spo0A* harboring compensatory mutations was PCR‐amplified from pFF‐247, using FFO‐1001/‐1002, digested with XhoI/SacI, and ligated into XhoI/SacI digested pFF‐260, resulting in pFF‐289 (*p*[SpoX*‐spo0A* ^*C*^]). In addition to the reporter system constructs, an empty control vector was generated by linearizing pFF‐191 via PCR with FFO‐994/‐1205, adding an additional NheI restriction site at the 5′ end. The resulting product was NheI digested and re‐ligated yielding pFF‐207 (*p*[ctl]). For generating a RS constitutively expressing either *cwp2* alone or coexpressing SpoY and *cwp2*, *cwp2* (5′UTR and 75 nt of CDS) was PCR‐amplified and gel‐purified as described above from *C. difficile* 630. FFO‐1354/‐1355 was used, adding a XhoI/SacI restriction site at the 5′/3′ end of the *cwp2* 5′UTR and start of CDS. The resulting product was XhoI/SacI digested and ligated into a similarly digested pFF‐191 and pFF‐185, yielding pFF‐344 (*p*[SpoY‐*cwp2*]) and pFF‐345 (*p*[*cwp2*]). Finally, a RS constitutively expressing either *cwpV* alone or coexpressing SpoX (long isoform) and *cwpV*, *cwpV* (5′UTR and 75 nt of CDS) was PCR‐amplified and gel‐purified from *C. difficile* 630 using FFO‐1469/‐1470, adding a XhoI/SacI restriction site at the 5′/3′ end of the *cwpV* 5′UTR and start of CDS. The resulting product was XhoI/SacI digested and ligated into a similarly digested pFF‐192 and pFF‐185, yielding pFF‐346 (*p*[SpoX‐*cwpV*]) and pFF‐347 (*p*[*cwpV*]).

### Plasmid conjugation

For conjugation purposes, plasmids were transformed using 80 μl of electro competent *E. coli* CA434 mixed with 100–500 ng of plasmids in a prechilled electroporation cuvette. Following electroporation with 1.8 kV, 200 Ω, 4–5 s, cells were recovered for 4 h at 37°C in 1 ml LB. Colonies harboring the plasmid were selected on LB supplemented with CHL and confirmed via colony PCR. Conjugation was performed according to Kirk & Fagan (2016), as published previously (Kirk & Fagan, 2016; Fuchs *et al*, 2021).

### Generation of deletion and insertion strains

Gene deletions were constructed using homologous recombination as previously published (Cartman *et al*, 2012; Fuchs *et al*, 2021). In short, plasmids were conjugated in *C. difficile* 630 strains as described above. Following conjugation, colonies were screened for the first recombination event via PCR. Positive recombinants were streaked on nonselective BHI, followed by incubation for 2–3 days. Growth was harvested using 900 μl 1× PBS, and 50 μl of a 10^−4^ and 10^−5^ dilution of the mixtures were streaked either on CDMM supplemented with 50 μg/ml fluorocytosine for pJAK112 derived plasmids, or on TY containing 4% w/v xylose for pJAK184 derived plasmids. Once colonies appeared, 8–15 were restreaked to purity and tested for secondary recombination events via PCR. To test for plasmid loss, colonies were simultaneously streaked on selective plates containing TAP. Sanger sequencing was applied to confirm successful deletions and insertions.

### RIL‐seq (RNA interaction by ligation and sequencing)

#### RIL‐seq experimental procedure

RIL‐seq was performed, following the original protocol published by Melamed and collogues with minor alterations (Melamed *et al*, 2016, 2018). Briefly, *C. difficile* 630 WT and Hfq‐FLAG were grown in sterile filtered TY in four biological replicates until OD_600_ of ∼ 1.2 (transition phase; Saujet *et al*, 2011; Hofmann *et al*, 2018). Of each replicate, 100 ODs were harvested (4,500 *g*, 15 min at 4°C), resuspended in 10 ml ice‐cold 1× PBS and irradiated with UV light (254 nm, 80,000 mJ/cm^2^). Following centrifugation (4,500 *g*, 15 min at 4°C), pellets were resuspended in 800 μl of ice‐cold wash buffer (50 mM NaH_2_PO_4_, 300 mM NaCl, 0.05% Tween, 1:200 diluted protease inhibitor cocktail set III [Calbiochem]) supplemented with 0.1 U/μl of recombinant RNase inhibitor (wash buffer‐RIn, Takara). Mechanical cell lysis was achieved by mixing each sample with 0.1 mm glass beads and grinding in a Retsch mixer mill at a frequency of 30/s for 5 min. The grinding was repeated four times; after each step, the adaptors were placed on ice for 2 min. The resulting supernatant was transferred to a new tube (17,000 *g*, 2 min at 4°C), 400 μl wash buffer‐RIn was added to the glass beads and the grinding was repeated once more. For Hfq co‐immunoprecipitations, the accumulated lysates were incubated with protein A/G magnetic beads (Thermo‐Fisher) precoupled with anti‐Flag antibody (M2 monoclonal antibody, Sigma‐Aldrich) for 2 h at 4°C with rotation, followed by three washing steps with wash buffer‐RIn. Next, samples were treated with 480 μl of wash buffer supplemented with RNase A/T1 (1:520, Thermo Fisher Scientific) for 5 min at 22°C, trimming any exposed RNA ends. The process was stopped by washing each sample with 200 μl of wash buffer supplemented with SUPERase In RNase inhibitor (final concentration of 0.1 U/μl, Ambion) three times for 5 min at 4°C. Following PNK treatment for 2 h at 22°C (1× PNK buffer A, 1 mM ATP, 1 U/μl recombinant RNAse inhibitor, 0.5 U/μl T4 PNK [New England BioLabs]), samples were washed again three times with wash buffer‐RIn. Hfq‐bound RNAs were then proximity ligated with T4 RNA ligase I (1× T4 RNA ligase buffer, 9% DMSO, 1 mM ATP, 20% PEG 8000, 0.6 U/μl recombinant RNase inhibitor, 2.7 U/μl T4 RNA ligase I [New England BioLabs]) overnight at 22°C with agitation, followed by three washing steps with wash buffer‐RIn at 4°C. Finally, RNA was eluted by incubating the beads with Proteinase K (50 mM Tris–HCl pH 7.8, 50 mM NaCl, 1% SDS, 5 mM EDTA pH 8.0, 5 mM β‐mercaptoethanol, 0.1 U/μl recombinant RNase inhibitor, 0.33 mg/ml Proteinase K [Thermo Fisher Scientific]) for 2 h at 55°C. RNA purification was achieved using TRIzol LS according to the manufacturer's instructions. Purified RNA was resuspended in 7 μl of nuclease‐free water and quality controlled on a Bioanalyzer Pico RNA chip.

cDNA library preparation and sequencing were performed by Vertis Biotechnologie AG. First, oligonucleotide adapters were ligated to the RNA 5′ and 3′ ends followed by first‐strand cDNA synthesis using M‐MLV reverse transcriptase and the 3′ adapters as primers. The resulting cDNAs were PCR‐amplified using a high‐fidelity DNA polymerase for 14–16 cycles. Next, cDNA was purified using the Agencourt AMPure XP kit (Beckman Coulter Genomics) and quality controlled by capillary electrophoresis on a Shimadzu MultiNA microchip electrophoresis system. For Illumina NextSeq sequencing, the samples were pooled in approximately equimolar amounts. The resulting cDNA pool was then size fractionated in the size range of 170–400 bp by polyacrylamide gel electrophoresis and paired‐end sequenced on an Illumina HighSeq system using 2 × 150 bp read lengths.

#### RIL‐seq data analysis

Sequencing and mapping results are listed in Dataset EV1. Data analysis was performed as described by Melamed *et al*, with a few modifications (Melamed *et al*, 2016, 2018). Briefly, raw reads were trimmed and quality filtered with BBDuk (min. phred‐score of 20, min. read length of 25). First mapping was conducted with BWA provided by the RIL‐seq computational pipeline (Li, 2013). The map_chimeric_fragments.py script provided by the RIL‐seq pipeline was used to classify fragments into single and chimeric. All parameters were set to default. Fragments that mapped within a distance of 1,000 nt or within the same transcript were considered single, whereas fragments that mapped to two different loci were considered chimeric. To test whether two fragments mapped within the same transcript, an additional annotation file for CP010905.2 was used. To decide whether the replicates can be considered reproducible, their correlation was computed as described by Melamed *et al*, by comparing the numbers of mapped fragments in corresponding genomic windows between each pair of libraries, for single and chimeric fragments, respectively (Melamed *et al*, 2018). To be able to use the ribozero option of RILseq_significant_regions.py, which excludes rRNAs from the analysis, the necessary BioCyc database was generated with Pathway Tools based on the CP010905.2 annotation (Karp *et al*, 2021). The high correlation coefficient of all *hfq*::3xFLAG pairs (*r* ≥ 0.79) allowed us to unify the replicates into a single dataset (Appendix Fig S1A). Fisher's exact test was applied to assign an odds ratio and a *P*‐value to each chimera. Chimeras with a min. odds ratio of 1 and a *P*‐value < 0.05 were considered significant and termed S‐chimeras. In addition, only S‐chimeras covered by ≥ 25 chimeric reads were considered for further analysis. The final output table was merged with the CP010905.2 annotation manually curated by assigning each interaction partner to one of the following categories: sRNA, 5′UTR, riboswitch, coding sequence (CDS), 3′UTR, CRISPR, tRNA, IGR, or anti‐sense (AS), resulting in Datasets EV2 and EV3 (Fuchs *et al*, 2021). Pairs can appear more than once if corresponding chimeric reads span multiple regions, such as an mRNA 5′UTR and CDS. Only counting each pair once, the dataset consisted of 1,569 unique interactions (1,198,921 chimeric reads) in the Hfq‐FLAG strain and six interactions (461 chimeric reads) in the WT, yielding similar numbers to published RIL‐seq data (Dataset EV1; Melamed *et al*, 2016; Matera *et al*, 2022).

To analyze intra‐operon RBS overlaps of interactions, an inhouse python script was used. First a database of all intra‐operon RBSs was build, based on the operon table published in Fuchs *et al* (2021). The table was filtered for operons with primary TSSs, excluding the first gene of an operon. Intra‐operon RBS regions were defined as 25 nt upstream and 20 nt downstream of the respective start codon of the gene. RNA1 and RNA2 of all S‐chimeras were searched for overlaps with intra‐operon RBS regions. For this purpose, the coordinates listed in the S‐chimera table were used, whereas the coordinate of either RNA1 start of last read and RNA2 start of first read were extended 100 nt in the respective direction. Additionally, the final output of interactions overlapping with intra‐operon RBS regions was curated manually (Dataset EV5).

#### RIL‐seq data visualization

To count how many chimeric or single reads overlap with given features in the CP010905.2 annotation, the script count_chimeric_reads_per_gene.py of the RILseq computational pipeline was used with slight modifications. We modified the script in a way that it would consider a list of feature categories instead of a single one. The following features were considered for the counting: CDS, 3′UTR, 5′UTR, ncRNA (which includes riboswitches, tmRNA, SRP_RNA, RNase_P_RNA, 6S RNA), sRNAs, rRNA, tRNA, antisense and intergenic regions. A fragment was counted as intergenic if it did not overlap with any of the other features. The minimal overlap between a fragment and a gene was set to 5. If any fragment overlapped with two features, both were counted.

To create images of specific interactions, bed files of chimeric fragments of single interactions were generated using the script generate_BED_file_of_endpoints.py of the RIL‐seq computational pipeline. The bed files were visualized with IGV 2.12.3, and further processed with Inkscape 0.92.4 for the respective figure (Fig 2D). For coverage plots, these bed files were first converted into coverage files with bedtools genomecov, followed by plot generation using the R package Gviz (*e.g*., Fig 3D; Quinlan, 2014; Hahne & Ivanek, 2016).

For circos plot visualization of sRNA networks, data were obtained by using the script plot_circos_plot.py from the RIL‐seq computational pipeline. Circos plots were generated with Circos (Krzywinski *et al*, 2009). To avoid overloading the plots, only a fraction of the interactions were shown, as indicated in the respective figure description. For a more detailed visualization of all SpoY and SpoX target interactions, Cytoscape 3.9.1 was used. All targets (nodes) were included, with targets supported by ≥ 25 chimeras marked by a solid line, while targets supported by < 25 chimeras were highlighted with a dashed line. Target types were discriminated by color as indicated in the figure legend. Edge strengths correlate with the total number of chimeras supporting an individual interaction as listed in Dataset EV2.

### Hot phenol extraction of total RNA

Total RNA was extracted using the hot phenol protocol. Bacterial cultures were grown to the desired OD_600_, mixed with 0.2 volumes of STOP solution (95% ethanol, 5% phenol) and snap‐frozen in liquid nitrogen. Once thawed on ice, the cell suspension was centrifuged for 20 min, 4,500 rpm at 4°C and the supernatant discarded. For cell lysis, pellets were suspended in 600 μl of 10 mg/ml lysozyme in TE buffer (pH 8.0) and incubated at 37°C for 10 min. Next, 60 μl of 10% w/v SDS was added, respectively, and samples were mixed by inversion, and incubated in a water bath at 64°C, 1–2 min before adding 66 μl 3 M NaOAc, pH 5.2. Phase separation was induced by mixing samples with 750 μl of acid phenol (Roti‐Aqua phenol), followed by incubation for 6 min at 64°C, while regularly inverting the tubes. Samples were briefly placed on ice to cool before centrifugation for 15 min, 13,000 rpm at 4°C. The aqueous layer was transferred into a 2 ml phase lock gel tube (Eppendorf) and mixed with 750 μl chloroform (Roth) by shaking, followed by centrifugation for 12 min, 13,000 rpm at room temperature. For ethanol precipitation, the aqueous layer was transferred to a new tube, 2 volumes of a 30:1 EtOH:3 M NaOAc, pH 6.5 mix was added and incubated overnight at −20°C. Finally, samples were centrifuged, washed with cold 75% v/v ethanol and air‐dried for 15 min. Precipitated RNA was resuspended in 50 μl nuclease‐free water and stored at −80°C.

### Northern blotting

Cells were grown in TY (Figs 2, 3 and 5; Appendix Fig S5), BHI, TY, TY supplemented with 0.5% glucose (TYG) or 0.5% fructose (TYF) and 70:30 sporulation medium (Appendix Fig S3), or on 70:30 sporulation plates (Appendix Fig S6) as described in the respective figure legends. RNA was purified as described above. Samples were mixed with equal amounts of gel loading buffer II (95% formamide, 18 mM EDTA, 0,025% SDS, 2% bromphenolblue), boiled at 98°C for 5 min and cooled down on ice before loading on a denaturing 6% polyacrylamide gel containing 7 M urea. RNA was separated for 1 h and 50 min, 300 V and transferred onto a Hybond‐N+ membrane (GE Healthcare Life Sciences) at 4°C for 1 h, 50 V (~ 100 W) followed by UV irradiation (0.12 J/cm^2^). Once cross‐linked, membranes were pre‐hybridized 1 h in ROTI Hybri‐Quick Buffer (Roth) before adding ^32^P‐labeled DNA oligonucleotides. 5′‐labeling was performed by incubating 10 pmol oligonucleotide with 1 μl of ^32^P‐γ‐ATP (10 μCi/μl) and 5 U T4 Polynucleotide Kinase (Thermo Fisher Scientific) for 1 h at 37°C in a 10 μl reaction. Labeled oligonucleotides were purified using microspin G‐25 columns (GE Healthcare) according to the manufacturer's instructions. Following hybridization overnight at 42°C, membranes were washed three times with decreasing concentrations (5×, 1× and 0.5×) of SSC buffer (20× SSC: 3 M NaCl, 0.3 M sodium citrate, pH 7.0). Air‐dried membranes were then exposed onto a phosphor screen for 1–7 days, and signals were visualized on a Typhoon FLA 7000 phosphor imager. Following signal detection, the membranes were stripped (0.1% SDS in freshly boiled water, 15 min), and incubated with ROTI Hybri‐Quick Buffer (Roth) before adding a new ^32^P‐labeled DNA oligonucleotide.

### *In vitro* transcription and radiolabeling of RNA

For *in vitro* transcription of SpoY, SpoX, *spo0A* and associated mutants, pFF‐162/−163/−164/−245/−166/−248/−247/−167 and corresponding primers were used for template generation via Phusion High‐Fidelity PCR. Resulting PCR products were purified from 1% agarose gels with NucleoSpin Gel and PCR Clean‐Up Kit (Macherey‐Nagel) to prevent the production of side products during *in vitro* transcription. *In vitro* transcription was performed using the Invitrogen MEGAscript T7 Transcription Kit (Thermofisher Scientific) in 40 μl reactions according to the manufacturer's protocol, followed by DNase treatment (1 μl TURBO DNase, 2 U/μl for 15 min at 37°C). Resulting RNA fragments were separated on a denaturing urea PAGE with 6% polyacrylamide and 7 M urea, followed by ethidium bromide (Carl Roth) staining for 10 min and imaging using an Intas Gel Doc system. Bands of correct size were cut out in small pieces and transferred into 2 ml tubes. For RNA elution, 750 μl RNA elution buffer (0.1 M NaAc, 0.1% SDS, 10 mM EDTA) was added, and the samples were incubated at 4°C and 1,000 rpm overnight. Following centrifugation at 5,000 *g* and 4°C for 1 min, the supernatants were transferred to new tubes and RNA extraction was performed using a single phenol‐chloroform extraction step (ROTI phenol/chloroform/isoamylalkohol). Purified RNA was resuspended in 20 μl RNase‐free water and stored at −80°C. For radioactive labeling, 50 pmol of *in vitro* transcribed SpoY, SpoY*, SpoX, SpoX* or *spo0A* (5′UTR starting from pTSS and start of CDS) was dephosphorylated using 25 U of calf intestine alkaline phosphatase (NEB) in a 50 μl reaction volume and incubated for 1 h at 37°C. RNA was extracted again, using a single phenol‐chloroform extraction step (ROTI phenol/chloroform/isoamylalkohol) and resuspended in 16 μl RNase‐free water. Subsequently, 20 pmol of dephosphorylated and purified RNA was 5′ end‐labeled (20 μCi of ^32^P‐γATP) using 1 U of Polynucleotide Kinase (NEB) for 1 h at 37°C in a 20 μl reaction volume. Finally, the labeled RNA was purified on a G‐50 column (GE Healthcare) according to the manufacturer's instructions and extracted from a polyacrylamide gel as described above following visualization on a Phosphorimager (FLA‐3000 Series, Fuji). Purified RNA was resuspended in 10 μl RNase‐free water and stored at −80°C for up to 2 weeks.

### EMSA (electrophoretic mobility shift assays)

Electrophoretic mobility shift assays were performed by incubating 0.04 pmol of radio‐labeled sRNA either alone or with increasing concentrations of *in vitro* transcribed mRNA. Prior to incubation, labeled sRNA and unlabeled mRNA were denatured at 95°C for 1 min and chilled on ice for 5 min. All components were mixed to a final concentration of 1× structure buffer (10 mM Tris–HCl pH 7.0, 0.1 M KCl, 10 mM MgCl_2_), 0.004 pmol/μl sRNA, 0.1 μg/μl yeast RNA (Ambion) and mRNA ranging from 0 to 1,000 pmol/μl. For EMSAs analyzing SpoY interactions with *spo0A* (5′UTR starting from pTSS), 500 pmol/μl purified *C. difficile* Hfq_6_ was added as well (Fuchs *et al*, 2021). Reactions were incubated at 37°C for 1 h, stopped by adding 3 μl of 5× native loading dye (0.5× TBE, 50% glycerol, 0.2% xylene cyanol, 0.2% bromophenol blue) and directly loaded on a native 6% polyacrylamide gel at 4°C in 0.5% TBE at 300 V for 3–4 h. The gel was dried for 1 h at 80°C on a Gel Dryer 583 (Bio‐Rad) and visualized after appropriate exposure on a Phosphorimager (FLA‐3000 Series, Fuji).

### In‐line probing

In‐line probing exploits the natural instability of unpaired RNA that leads to differential degradation according to its structure, allowing elucidation of secondary structure information. In‐line probing assays were performed by incubating 0.2 pmol of labeled RNA (sRNA or *spo0A* mRNA starting from pTSS) either alone or with increasing concentrations of *in vitro* transcribed *spo0A* (0.2, 2, and 20 pmol) or SpoX (0.2, and 2 pmol) for 40 h at room temperature in 1× in‐line probing buffer (100 mM KCl, 20 mM MgCl_2_, 50 mM Tris–HCl, pH 8.3). Both, sRNAs and mRNA, were denatured at 95°C for 1 min and chilled on ice for 5 min before assembling the reactions. Ladders were prepared directly prior to loading. For the RNase T1 ladder, 0.2 pmol of labeled RNA was incubated with 8 μl of 1× sequencing buffer (Ambion) at 95°C for 1 min followed by the addition of 1 μl RNase T1 (0.1 U/ μl) and incubation at 37°C for 5 min. The alkaline hydrolysis ladder was prepared by incubating 0.2 pmol labeled sRNA with 9 μl of 1× alkaline hydrolysis buffer (Ambion) and incubated at 95°C for 5 min. All reactions were stopped by the addition of 10 μl of 2× colorless gel‐loading solution (10 M urea, 1.5 mM EDTA) and stored on ice. 0.2 pmol of labeled RNA mixed with 10 μl 2× colorless gel‐loading solution served as a control. Samples were resolved on a 10% (Fig 4B) or 6% (Fig EV2C; vol/vol) polyacrylamide, 7 M urea sequencing gel pre‐run for 30 min, 45 W prior to sample loading. The gel was dried for 2 h on a Gel Dryer 583 (Bio‐Rad) and visualized after appropriate exposure on a Phosphorimager (FLA‐3000 Series, Fuji).

### Reporter system assay

Single colonies of *C. difficile* 630 ΔSpoY harboring pFF‐185/‐186/‐191/‐254/‐285/‐344, or ‐345 (FFS‐536/‐535/‐537/‐779/‐798/‐929/‐930) and *C. difficile* ΔSpoX harboring pFF‐185/‐187/‐192/‐260/‐289/‐346, or ‐347 (FFS‐539/‐538/‐540/‐785/‐802/‐931/‐932) were used to inoculate overnight cultures in biological triplicates in sterile filtered TY supplemented with TAP. Main cultures were inoculated by diluting overnight cultures 1:330 in sterile filtered TY supplemented with TAP and grown till ME growth phase (OD_600_ of ∼ 0.5) before harvesting 0.07 ODs, respectively, in a 96‐well plate (5 min 4,500 *g*). Cell pellets were resuspended in 200 μl 4% PFA and incubated at room temperature for 30 min in the dark. Following cell fixation, samples were washed three times in 200 μl 1× PBS, resuspended in 30 μl 1× PBS and incubated overnight at 4°C in the dark, allowing full maturation of mCherry. Subsequently, samples were diluted in 1× PBS to a final volume of 200 μl and mCherry fluorescence was detected using an Agilent NovoCyte Flow Cytometer. The sample acquisition threshold was set to 5,000, ungated in the FSC‐H channel, and a maximum of 100,000 events. Three parameters were recorded for each particle, including FSC‐H, SSC‐H and PE‐Texas Red‐H. sRNA expression was verified via northern blot analysis of samples grown in the same growth conditions (Appendix Fig S5B and C).

### Western blotting

To test Spo0A expression in a SpoY and SpoX deletion mutant and corresponding overexpression strains, FFS‐591, FFS‐593, FFS‐535, FFS‐594, and FFS‐538 were inoculated into sterile filtered TY supplemented with TAP in biological triplicates from single colonies. Main cultures were inoculated by diluting overnight cultures to OD_600_ = 0.05 in sterile filtered TY containing TAP. For Western blot analysis samples were taken at mid‐exponential (5.5 h post inoculation, OD_600_ of ∼ 0.5) and stationary phase (9 h post inoculation, OD_600_ of ∼ 1.3) by harvesting 2 OD units via centrifugation for 5 min at 5,000 *g*. Pellets were frozen overnight at −20°C, followed by cell resuspension in 50 μl 1× PBS and incubation for 50 min at 37°C, leading to cell lysis (Fagan & Fairweather, 2011). Subsequently, cell lysates were mixed with equal amounts of 2× protein loading dye and boiled for 5 min at 95°C. Of each sample, 0.3 OD (15 μl) was loaded and separated on a 15% SDS–polyacrylamide gel followed by transfer of proteins to a Protran 0.2 μm NC membrane (Amersham) at 4°C for 1.5 h, 340 mA using a semidry blotting system. Equal loading of protein samples was confirmed via Ponceau S staining (Sigma‐Aldrich) for 4 min. Staining was reversed by washing the stained membrane with 0.1 M NaOH for 1 min, followed by blocking in TBS‐T with 5% powdered milk for 1 h at room temperature. Subsequently, the membrane was incubated overnight at 4°C with anti‐Spo0A antibody (kindly provided by Amy Shen; Putnam *et al*, 2013) diluted 1:5,000 in TBS‐T with 5% powdered milk and washed again 3× in TBS‐T for 10 min. Following the last washing step, the membrane was incubated for 1 h at room temperature with anti‐mouse‐HRP antibody (RRID: AB_228307; Thermo Scientific # 31430) diluted 1:10,000 in TBS‐T with 5% powdered milk and finally washed 3× in TBS‐T for 10 min before adding ECL substrate (Amersham) for detection of HRP activity using a CCD camera (ImageQuant, GE Healthcare). sRNA expression was verified via northern blot analysis of samples grown in the same growth conditions (Fig 5B).

### RT‐qPCR analysis of sporulation genes

Glycerol stocks of *C. difficile* strains (FFS‐535, FFS‐538, FFS‐591, FFS‐593, and FFS‐594) were inoculated onto BHIS plates (BHI agar containing 5 g/l yeast extract [Roth] and 0.1% sterile filtered cysteine) supplemented with taurocholate (TA, 0.1% w/v) and TAP. Single colonies were then inoculated into liquid BHIS‐TA‐TAP media in biological triplicates and grown overnight. These cultures were diluted to OD_600_ = 0.05 with BHIS‐TA‐TAP media and grown till early stationary phase. Precultures were then diluted using BHIS‐TAP to OD_600_ = 0.05 and grown as main cultures until an OD_600_ of ~ 0.5. To induce sporulation, 120 μl of the main cultures were spread onto 70:30 agar plates (70% SMC media and 30% BHIS media) supplemented with TAP. Sporulating cultures were collected at 9 and 12 h after plating using phosphate‐buffered saline (PBS) and flash‐frozen immediately upon the addition of 0.2 volumes ice‐cold STOP mix (5% water‐saturated phenol [pH < 7.0] in ethanol). Total RNA was then isolated from collected samples using the hot‐phenol extraction procedure as described above. For DNA removal, 5 μg of total RNA was treated with DNase I (Thermo Scientific) for 1 h at 37°C and then further purified using a single phenol‐chloroform extraction step (ROTI Phenol/Chloroform/Isoamylalkohol). Purified RNA was resuspended in 50 μl RNase‐free water and stored at −80°C. Reverse transcription was performed using the M‐MLV Reverse Transcriptase Kit (Invitrogen) as per the manufacturer's instructions, with 1 μg of DNase I treated, purified total RNA and Random Hexamer Primer (Invitrogen). cDNA was then diluted 20‐fold, and 1 μl was used for each qPCR reaction along with 20 nM of gene‐specific oligonucleotides in a 10 μl reaction mix. qPCR was performed with Takyon™ No ROX SYBR 2× MasterMix blue dTTP (Eurogentec) reagent in technical duplicates using the QuantStudio™ 5 Real‐Time PCR cycler (Thermo Fisher Scientific) and following conditions: TakyonTM activation at 95°C for 3 min; DNA denaturing at 95°C for 10 s; 40 cycles of annealing and extension at 60°C for 60 s, followed by melting curve denaturation at 95°C for 15 s and melting curve analysis at 55–95°C using “step and hold” with 0.5°C and 10 s of incubation per step. Transcript levels were normalized to 5S rRNA and are displayed as ΔΔ*C*_t_ values, representing Log_2_‐fold change relative to FFS‐591 (WT‐*p*[ctl]). All oligonucleotide sequences used for RT‐qPCR are listed in Appendix Table S7. sRNA expression was verified via northern blot analysis of samples grown in the same growth conditions (Appendix Fig S6A).

### Sporulation frequencies

Sporulation assays were performed in 70:30 sporulation broth medium according to Edwards *et al* (2014) with minor alterations. In short, *C. difficile* cultures (FFS‐535, FFS‐538, FFS‐591, FFS‐593, and FFS‐594) were started in four biological replicates in BHIS medium supplemented with 0.1% taurocholate (TA) and 0.2% fructose until mid‐log phase (OD_600_ ≤ 0.9). Cultures were then back‐diluted in 70:30 medium to OD_600_ = 0.01 and monitored for the production of spores. At each timepoint (6, 12, 24, and 48 h post inoculation), samples were taken, serially diluted, and spotted (10 μl spots in technical triplicates) on BHIS‐TA plates and incubated for 24–48 h to enumerate total number of CFU (spores and vegetative cells). Simultaneously, 500 μl from each culture was removed, mixed 1:1 with 95% EtOH, and incubated for 30 min to kill all vegetative cells. EtOH‐treated samples were then serially diluted, similarly plated, and incubated, representing the spore CFU. The sporulation frequency was determined by dividing the number of spores by the total number of CFUs at each time point (spore ratio), multiplied by 100 (percentage of spores formed).

For phase‐contrast microscopy, *C. difficile* strains were grown in 70:30 sporulation medium as described above in four biological replicates. At 12, 24, and 48 h post inoculation, 1 ml of culture was removed from the anaerobic chamber, centrifuged at full speed for 30 s, and resuspended in ∼10–30 μl of supernatant. Microcopy slides were prepared by placing 2 μl of the concentrated cultures onto thin 1% agarose pads that were applied directly to the surface of the slide. Phase‐contrast microscopy was performed using a HC PLAN FLUOTAR 100×/1.32 PH3 oil immersion objective on a LEICA DM2500 microscope. Two fields of view for each strain and replicate were acquired and used to calculate the percentage of spores (the number of spores divided by the total number of spores, prespores, and vegetative cells; 300 cells per field of view were analyzed).

### Murine model of *C. difficile* infection

All animal experiments were performed in agreement with the guidelines of the Helmholtz Centre for Infection Research (HZI), Brunswick, Germany, the national animal protection law (TierSchG), the animal experiment regulations (TierSchVersV), and the recommendations of the Federation of European Laboratory Animal Science Association (FELASA). Mice experiments were approved by the Lower Saxony State Office for Nature, Environment and Consumer Protection (LAVES), Oldenburg, Lower Saxony, Germany; permit No. 33.19‐42502‐04‐19/3126.

C57BL/6N SPF mice were maintained (including housing) at the animal facilities of the HZI under enhanced specific pathogen‐free (SPF) conditions for at least 2 weeks before the start of the experiment. Female mice aged between 12 and 14 weeks were used. Sterilized food and water were provided *ad libitum*. Mice were kept under a strict 12 h light cycle (lights on at 7:00 am and off at 7:00 pm) and housed in groups of up to six mice per cage. All mice were euthanized by asphyxiation with CO_2_ and cervical dislocation.

Infection experiments were performed with *C. difficile* 630 strain FFS‐01 (WT), FFS‐491 (ΔSpoY), and FFS‐492 (ΔSpoX). SPF mice were weighted and treated with 10 mg/kg clindamycin 24 h prior to infection, administered via intraperitoneal injection to induce susceptibility to *C. difficile* infection (Theriot *et al*, 2016). Spores were heat‐treated at 65°C for 20 min before infection to kill remaining vegetative cells. Mice were infected with 10^4^ *C. difficile* spores in 200 μl 1× PBS administered via oral gavage. Following infection, mice were monitored and scored daily for symptoms of clinically severe CDI including behavior, posture, fur and skin, provoked behavior, weight loss, and feces consistency. Mice showing signs of CDI were monitored twice a day and euthanized after losing 20% of their initial weight or developing severe clinical signs of features listed above.

For quantification of bacterial burden, fresh fecal samples were collected at different time points, their weight recorded, supplemented with 1.0 mm diameter zirconium/glass beads in 1 ml 1× PBS, and subsequently homogenized for 50 s with Mini‐Beadbeater‐96 (Biospec). To determine colony forming units (CFUs), serial dilutions of homogenized samples were plated on bioMérieuxTM *C. difficile* agar. For the quantification of spores, aliquots of homogenized samples were incubated 65°C for 20 min to kill remaining vegetative cells and plated on bioMérieux™ *C. difficile* agar pretreated with 0.1% taurocholic acid to induce germination. Plates were cultured at 37°C for 48 h in anaerobic jars before counting. CFUs of *C. difficile* were calculated by normalization to feces weight.

### Prediction of RNA folding and sRNA target interactions

Secondary structures of sRNAs were predicted with the RNAfold WebServer, while RNAcofold was used to predict secondary structures of single stranded sRNA and mRNA sequences upon duplex formation (Bernhart *et al*, 2006; Lorenz *et al*, 2011). In both cases, structures were visualized with VARNA (Darty *et al*, 2009).

Potential sRNA‐target interactions were predicted using IntaRNA by uploading either the SpoY, SpoX or *spo0A* sequence in combination with interaction partners revealed by RIL‐seq analysis (Dataset EV2; Mann *et al*, 2017). Default settings were used.

### Prediction of sRNA target motif

Motif search and generation of sequence logos was accomplished with MEME version 5.4.1 (Bailey & Elkan, 1994). For both sRNAs, all target sequences were extracted from the RIL‐seq data, including target interactions supported by < 25 chimeras (Dataset EV2). For this purpose, the coordinates listed in the S‐chimera table for “start of first read” and “start of last read” were used and extended 50 nt downstream. The resulting sequences were uploaded to MEME for target motif identification (SpoY = 28 targets, SpoX = 42 targets). The number of motifs to be found by MEME was set to 5 and only the given strand was searched. All other settings were left at default.

### Quantification and statistical analysis

Statistical analysis of RIL‐seq results is described above and was performed according to the original protocol (Melamed *et al*, 2016, 2018). Quantification and analysis of Western blot, northern blot, and EMSA signals as well as in‐line probing and microscopy images was performed with ImageJ (Schneider *et al*, 2012). GraphPad Prism 9 was used for all statistical analyses and data visualizations, in combination with Inkscape 0.92.4 (Schneider *et al*, 2012). Sample sizes and detailed descriptions of statistical analyses are indicated in the figure legends and method section for each experiment separately.

## Author contributions

**Manuela Fuchs:** Conceptualization; data curation; formal analysis; validation; investigation; visualization; methodology; writing – original draft; writing – review and editing. **Vanessa Lamm‐Schmidt:** Data curation; formal analysis; visualization; methodology; writing – review and editing. **Tina Lenče:** Data curation; formal analysis; validation; investigation; methodology; writing – review and editing. **Johannes Sulzer:** Formal analysis; validation; investigation; methodology; writing – review and editing. **Arne Bublitz:** Data curation; formal analysis; validation; investigation; methodology; writing – review and editing. **Janet Wackenreuter:** Data curation; formal analysis; validation; investigation; methodology; writing – review and editing. **Milan Gerovac:** Software; visualization; writing – review and editing. **Till Strowig:** Conceptualization; investigation; methodology; writing – review and editing. **Franziska Faber:** Conceptualization; supervision; funding acquisition; methodology; writing – original draft; writing – review and editing.

## Disclosure and competing interests statement

The authors declare that they have no conflict of interest.

## Supporting information



Appendix

Expanded View Figures PDF

Dataset EV1

Dataset EV2

Dataset EV3

Dataset EV4

Dataset EV5

PDF+

Source Data for Figure 2

Source Data for Figure 3

Source Data for Figure 4

Source Data for Figure 5

Source Data for Figure 6

Source Data for Figure 7

Source Data for Figure 8

## Data Availability

All RNA‐sequencing data are available at the National Center for Biotechnology Information Gene Expression Omnibus database (https://www.ncbi.nlm.nih.gov/geo) under accession number GSE213005. The RIL‐seq dataset can be accessed via an RNA–RNA interactome browser (https://resources.helmholtz‐hiri.de/rilseqcd/). Phase‐contrast microscopy images (Fig 7) are available on BioImage Archive under accession number S‐BIAD622.
