# Supplementary material for: A network of small RNAs regulates sporulation initiation in Clostridioides difficile
Source: EMBO J. 2023 May 4;42(12):e112858. doi: 10.15252/embj.2022112858 (PMC10267692; doi:10.15252/embj.2022112858)
Supplement: Supplementary file 1 — Appendix [file EMBJ-42-e112858-s013.pdf]

Supplementary Information

A network of small RNAs regulates sporulation initiation in *Clostridioides difficile*

Manuela Fuchs [1,2], Vanessa Lamm-Schmidt [1,2], Tina Lenče [2], Johannes Sulzer [2], Arne Bublitz [3], Janet Wackenreuter [1], Milan Gerovac [2], Till Strowig [3,4], Franziska Faber [1,2] *

[1] Helmholtz Institute for RNA-based Infection Research (HIRI), Helmholtz Centre for Infection Research (HZI), Würzburg, Germany

[2] Julius-Maximilians-University of Würzburg (JMU), Faculty of Medicine, Institute for Molecular Infection Biology (IMIB), Germany

[3] Helmholtz Centre for Infection Research (HZI), Braunschweig, Germany

[4] German Center for Infection Research (DZIF), partner site Hannover-Braunschweig, Germany

* Correspondence:
Franziska Faber: phone +49-931-3186280; email: franziska.faber@uni-wuerzburg.de

Keywords: *Clostridioides difficile*, RIL-seq, small RNA, Hfq, Spo0A

This PDF file includes:

Appendix Figures S1 to 6

Appendix Table S1 to 3

SI References

Contents

Appendix Figures 3
- Appendix Figure S1 3
- Appendix Figure S2 4
- Appendix Figure S3 5
- Appendix Figure S4 6
- Appendix Figure S5 7
- Appendix Figure S6 8

Appendix Tables 9
- Appendix Table S1 9
- Appendix Table S2 13
- Appendix Table S3 16

SUPPLEMENTARY REFERENCES 24

APPENDIX FIGURES

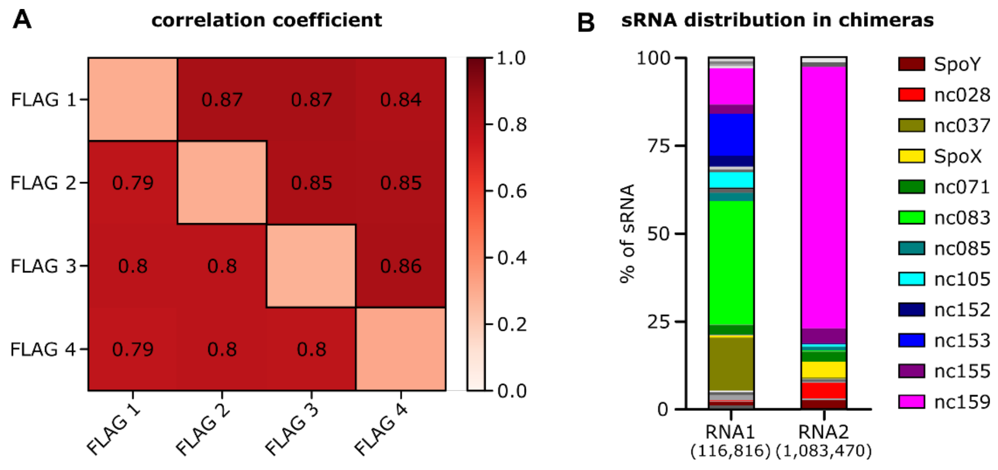

Appendix Figure S1: Hfq serves as a platform for RNA-RNA interactions in *C. difficile*. (A) Replicate reproducibility calculated as correlation coefficient by comparing the numbers of mapped fragments in corresponding genomic windows between each pair of libraries, for single (below diagonal) and chimeric (above diagonal) fragments, respectively. **(B)** Distribution of sRNAs in chimeric fragments, where RNA1 constitutes the 5'end and RNA2 the 3'end of a chimera (n=4). sRNAs that are present in ≥ 1.5% off all chimeras in either RNA1 or RNA2 are highlighted in order of genomic location. A total of 116,816 chimeric reads mapped to sRNAs in RNA1 and 1,083,470 to sRNAs in RNA2**.**

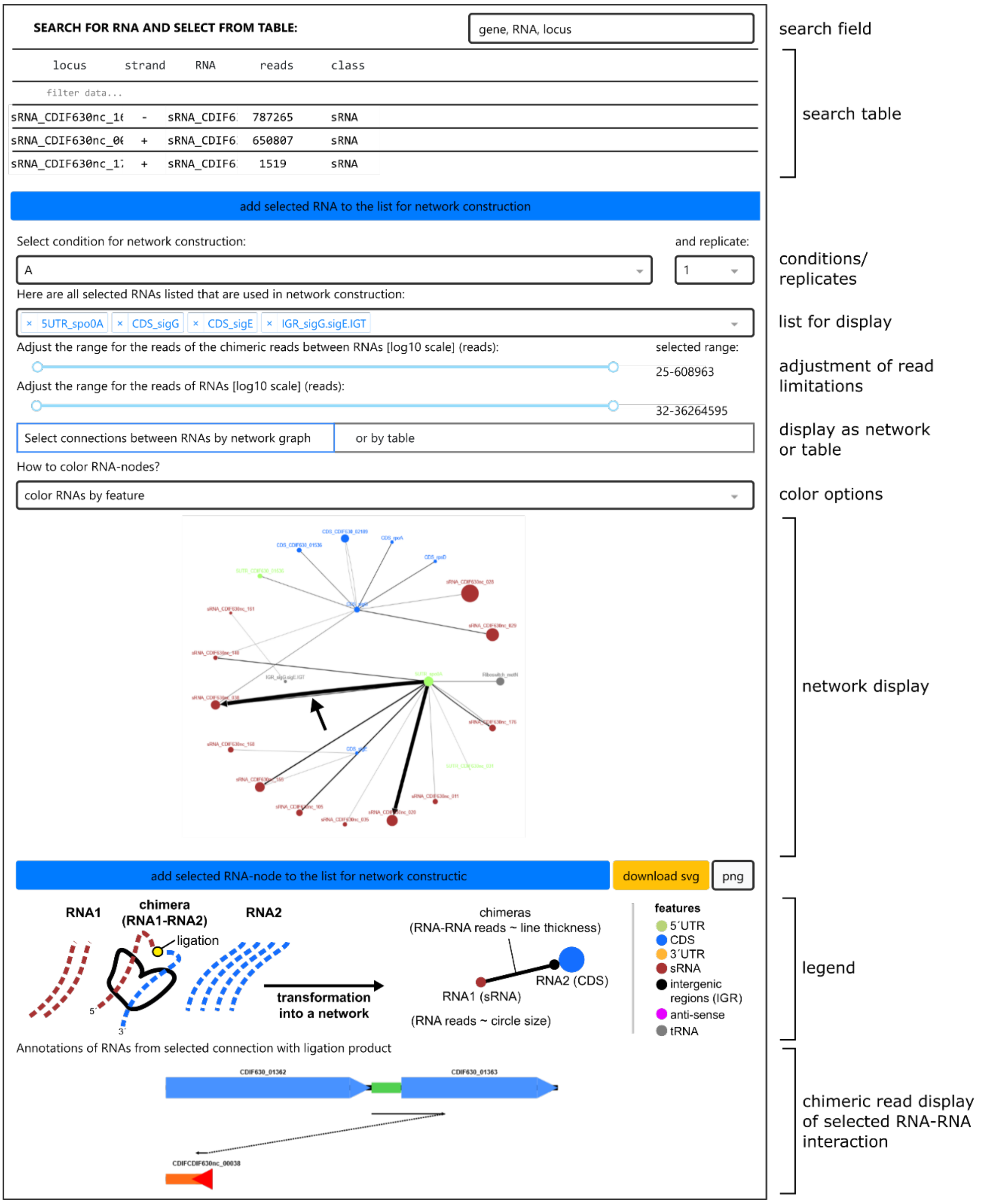

Appendix Figure S2: Rilseqcd is a web-browser that allows easy access to our RIL-seq data and an interactive search for RNA-RNA interactions. Screenshot of the RIL-seq browser accessible *via* https://resources.helmholtz-hiri.de/rilseqcd/. Details explaining the available options are given on the right. So far only one condition and one replicate are available, the latter because all four replicates have been pooled into a single dataset. Specific targets can be searched and added to the network display either *via* the search bar and table at the top, or by directly typing into the "list for display" field. If no targets are selected, a network of all detected interactions will be shown. By clicking on specific interaction in the "network display", a schematic representation of the selected RNA-RNA interaction will appear on the bottom.

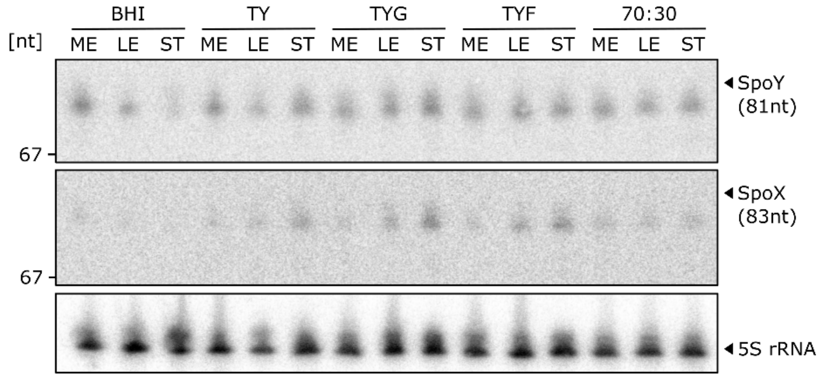

Appendix Figure S3: SpoY and SpoX expression in selected growth conditions. Northern blot validation of SpoY and SpoX expression in mid-exponential (ME), late exponential (LE) and stationary (ST) phase of growth in either BHI, TY, TY supplemented with 0.5% glucose (TYG) or 0.5% fructose (TYF) or 70:30 sporulation medium respectively. 5S rRNA served as a loading ctl. A representative image of three independent experiments is shown.

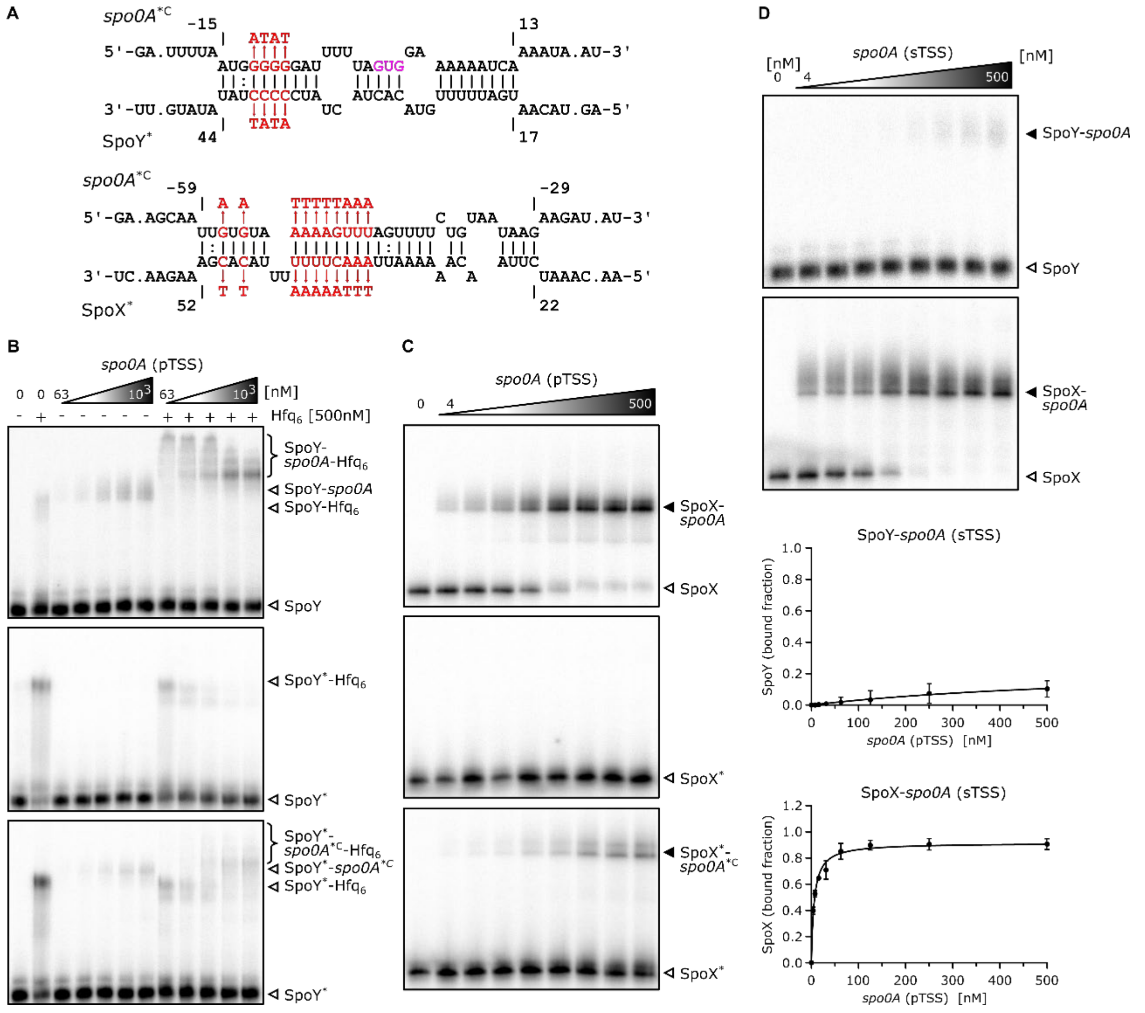

Appendix Figure S4: **SpoY and SpoX directly interact with the *spo0A* mRNA *in vitro*. (A)** *In silico* predicted SpoY-*spo0A* and SpoX-*spo0A* interaction sites (IntaRNA[1]). Mutations introduced in the sRNA seed region as well as compensatory mutations in the *spo0A* target region are highlighted in red. The *spo0A* nucleotide position is calculated relative to the *spo0A* start codon (highlighted in pink). **(B-C)** EMSAs performed with either ^{32}P-labeled SpoY (B) or SpoX (short isoform) (C) with increasing concentrations of the long *spo0A* 5'UTR and first 69 nt of CDS, respectively. Purified Hfq was added to facilitate SpoY-*spo0A* complex formation. Mutating the respective sRNA seed region (SpoY*/SpoX*) abolished the interaction, while introducing compensatory mutations into the *spo0A* target region (*spo0A*^{*C}) slightly rescued the complex formation. A representative image of three independent experiments is shown, respectively. **(D)** EMSAs and corresponding quantifications (n=3) were performed with either ^{32}P-labeled SpoY or SpoX (short isoform) with increasing concentrations of the short *spo0A* 5'UTR and first 69 nt of CDS, respectively.

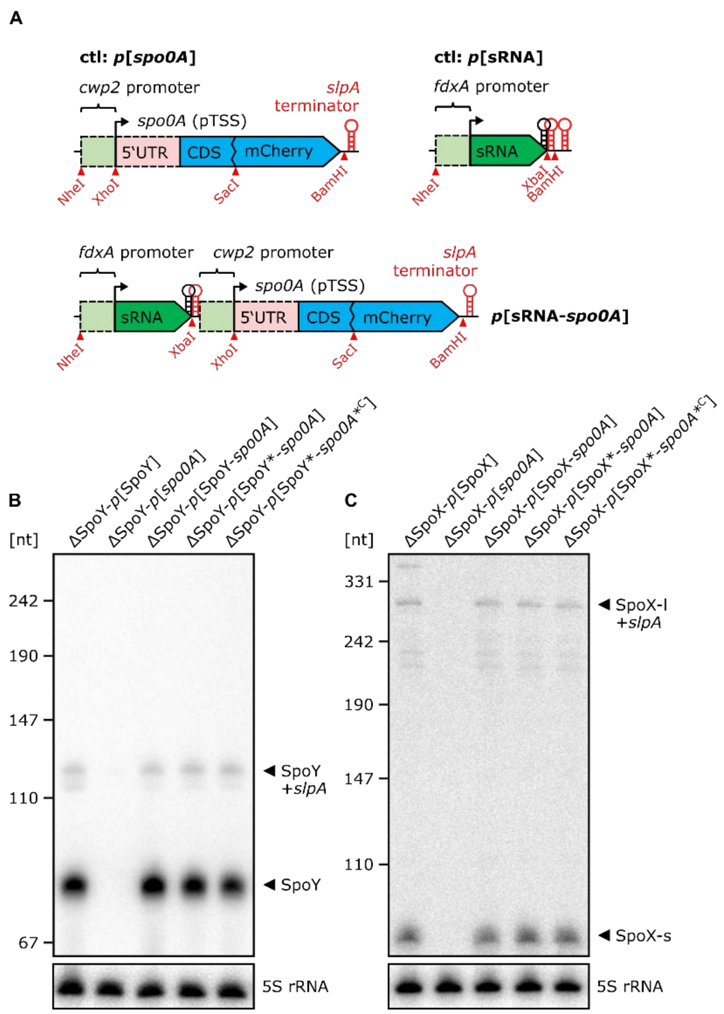

Appendix Figure S5: **SpoY and SpoX directly interact with the *spo0A* mRNA *in vivo.* (A)** Schematic representation of translational fusion constructs designed for *in vivo* reporter system assays. Restriction sites that allow easy exchange of each component individually are annotated. "sRNA" refers to either SpoY or SpoX (long isoform. mCherry fused to the *spo0A* 5'UTR (starting from the pTSS) and beginning of CDS serves as a readout. For Figure EV4, *spo0A* was replaced by either *cpw2* or *cwpV* 5'UTR and first 20 aa of CDS. **(B-C)** Northern blot validation of sRNA expression from reporter constructs grown in TY till ME growth phase in the respective sRNA deletion mutant. A representative image of three independent experiments is shown, respectively.

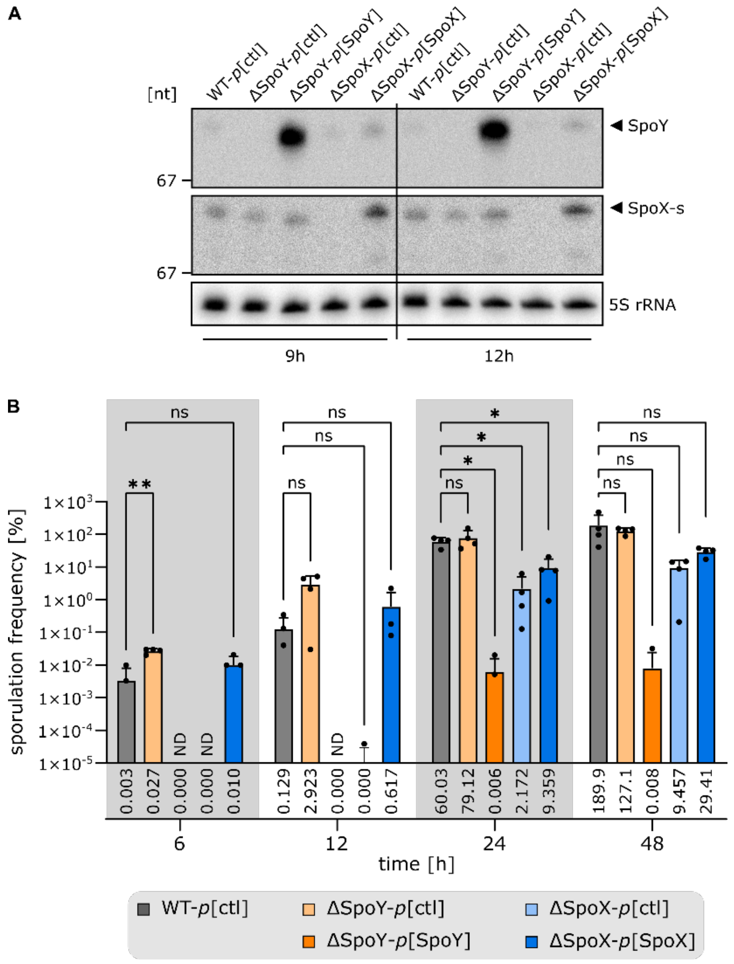

Appendix Figure S6: sRNA mediated regulation of *spo0A* affects sporulation specific genes and sporulation frequencies. (A) Northern blot validation of sRNA expression in conditions used for Figure 6. RNA was extracted from samples (n = 3) taken at 9 h and 12 h post induction of sporulation on 70:30 sporulation plates. **(B)** Sporulation frequencies (n=4) of a WT strain (*p*[ctl]), sRNA knock-out mutants (ΔSpoY/ΔSpoX-*p*[ctl]) and strains constitutively expressing the respective sRNA (ΔSpoY/ΔSpoX-*p*[ΔSpoY/ΔSpoX]) at 6 h, 12 h, 24 h and 48 h post inoculation of 70:30 liquid sporulation medium. ND: not determined – no viable spores. 2-way ANOVA with Dunnett's multiple comparison test was used to calculate statistical significance. Not significant (ns) $P > 0.05$; (*) $P \leq 0.05$; (**) $P \leq 0.01$.

APPENDIX TABLES

Appendix Table S1: Bacterial strains used in this study.

Strain	Relevant markers / Genotype	Origin
Escherichia coli		
TOP10	F- mcrA Δ(mrr-hsdRMS-mcrBC) φ80lacZΔM15 ΔlacX74 nupG recA1 araD139 Δ(ara-leu)7697 galE15 galK16 rpsL(StrR) endA1 λ-.	Invitrogen
CA434	thi-1 hsdS20 (r-B, m-B) supE44 recAB ara-14 leuB5proA2 lacY1 galK rpsL20 (strR) xyl-5 mtl-1.	Dieter Jahn
FFS-204	Top 10 carrying pFF-53.	this study
FFS-210	CA434 carrying pFF-53.	this study
FFS-420	StrataClone SoloPack Competent Cells carrying pFF-162.	this study
FFS-421	StrataClone SoloPack Competent Cells carrying pFF-163.	this study
FFS-422	StrataClone SoloPack Competent Cells carrying pFF-164.	this study
FFS-694	StrataClone SoloPack Competent Cells carrying pFF-245.	this study
FFS-424	StrataClone SoloPack Competent Cells carrying pFF-166.	this study
FFS-697	StrataClone SoloPack Competent Cells carrying pFF-248.	this study
FFS-696	StrataClone SoloPack Competent Cells carrying pFF-247.	this study
FFS-425	StrataClone SoloPack Competent Cells carrying pFF-167.	this study
FFS-428	Top 10 carrying pFF-170.	this study
FFS-450	CA434 carrying pFF-170.	this study
FFS-429	Top 10 carrying pFF-171.	this study
FFS-451	CA434 carrying pFF-171.	this study

FFS-479	Top 10 carrying pFF-185 (*p*[*spo0A*]).	this study
FFS-480	Top 10 carrying pFF-186 (*p*[SpoY]).	this study
FFS-505	Top 10 carrying pFF-191 (*p*[SpoY-*spo0A*]).	this study
FFS-714	Top 10 carrying pFF-254 (*p*[SpoY*-*spo0A*]).	this study
FFS-31	Top 10 carrying pFF-285 (*p*[SpoY*-*spo0A*^{*C}]).	this study
FFS-481	Top 10 carrying pFF-187 (*p*[SpoX]).	this study
FFS-506	Top 10 carrying pFF-192 (*p*[SpoX-*spo0A*]).	this study
FFS-720	Top 10 carrying pFF-260 (*p*[SpoX*-*spo0A*]).	this study
FFS-606	Top 10 carrying pFF-289 (*p*[SpoX*-*spo0A*^{*C}]).	this study
FFS-502	CA434 carrying pFF-185 (*p*[*spo0A*]).	this study
FFS-503	CA434 carrying pFF-186 (*p*[SpoY]).	this study
FFS-529	CA434 carrying pFF-191 (*p*[SpoY-*spo0A*]).	this study
FFS-753	CA434 carrying pFF-254 (*p*[SpoY*-*spo0A*]).	this study
FFS-771	CA434 carrying pFF-285 (*p*[SpoY*-*spo0A*^{*C}]).	this study
FFS-504	CA434 carrying pFF-187 (*p*[SpoX]).	this study
FFS-530	CA434 carrying pFF-192 (*p*[SpoX-*spo0A*]).	this study
FFS-759	CA434 carrying pFF-260 (*p*[SpoX*-*spo0A*]).	this study
FFS-775	CA434 carrying pFF-289 (p[SpoX*-spo0A*C]).	this study
FFS-564	Top 10 carrying pFF-207 (*p*[ctl]).	this study
FFS-586	CA434 carrying pFF-207 (*p*[ctl]).	this study

FFS-918	Top 10 carrying pFF-344 (*p*[SpoY-*cwp2*]).	this study
FFS-919	Top 10 carrying pFF-345 (*p*[*cwp2*]).	this study
FFS-920	Top 10 carrying pFF-346 (*p*[SpoX-*cwpV*]).	this study
FFS-921	Top 10 carrying pFF-347 (*p*[*cwpV*]).	this study
FFS-923	CA434 carrying pFF-344 (*p*[SpoY-*cwp2*]).	this study
FFS-924	CA434 carrying pFF-345 (*p*[*cwp2*]).	this study
FFS-925	CA434 carrying pFF-346 (*p*[SpoX-*cwpV*]).	this study
FFS-926	CA434 carrying pFF-347 (*p*[*cwpV*]).	this study
Clostridioides difficile		
630	630 wild-type strain.	DSMZ
FFS-220	630 *hfq*::3xFLAG.	this study
FFS-491	630 ΔSpoY (ΔCDIF630nc_020).	this study
FFS-492	630 ΔSpoX (ΔCDIF630nc_038).	this study
FFS-536	630 ΔSpoY carrying pFF-185 (*p*[*spo0A*]).	this study
FFS-535	630 ΔSpoY carrying pFF-186 (*p*[SpoY]).	this study
FFS-537	630 ΔSpoY carrying pFF-191 (*p*[SpoY-*spo0A*]).	this study
FFS-779	630 ΔSpoY carrying pFF-254 (*p*[SpoY*-*spo0A*]).	this study
FFS-798	630 ΔSpoY carrying pFF-285 (*p*[SpoY*-*spo0A*^C*]).	this study
FFS-539	630 ΔSpoX carrying pFF-185 (*p*[*spo0A*]).	this study
FFS-538	630 ΔSpoX carrying pFF-187 (*p*[SpoX]).	this study

FFS-540	630 ΔSpoX carrying pFF-192 (*p*[SpoX-*spo0A*]).	this study
FFS-785	630 ΔSpoX carrying pFF-260 (*p*[SpoX*-*spo0A*]).	this study
FFS-802	630 ΔSpoX carrying pFF-289 (*p*[SpoX*-$spo0A^{*C}$]).	this study
FFS-591	630 WT carrying pFF-207 (*p*[ctl]).	this study
FFS-593	630 ΔSpoY carrying pFF-207 (*p*[ctl]).	this study
FFS-594	630 ΔSpoX carrying pFF-207 (*p*[ctl]).	this study
FFS-929	630 ΔSpoY carrying pFF-344 (*p*[SpoY-*cwp2*]).	this study
FFS-930	630 ΔSpoY carrying pFF-345 (*p*[*cwp2*]).	this study
FFS-931	630 ΔSpoX carrying pFF-346 (*p*[SpoX-*cwpV*]).	this study
FFS-932	630 ΔSpoX carrying pFF-347 (*p*[*cwpV*]).	this study

Appendix Table S2: Plasmids used in this study.

Plasmid	Description	Origin
pJAK184	To generate gene deletions or insertions in *C. difficile* by homologous recombination. Carrying *E. coli mazF* for counter selection in *C. difficile*.	[2]
pFF-53	Derived from pJAK184 for generating a *hfq*::3xFLAG strain.	this study
pSC-A-amp/kan	For cloning of PCR products using the StrataClone PCR Cloning Kit.	Agilent Technologies, Inc.
pFF-162	Derived from pSC-A-amp/kan for PCR amplification and subsequent *in vitro* transcription of SpoY (CDIF630nc_020).	this study
pFF-163	Derived from pSC-A-amp/kan for PCR amplification and subsequent *in vitro* transcription of SpoY* (CDIF630nc_020 with a mutated seed region).	this study
pFF-164	Derived from pSC-A-amp/kan for PCR amplification and subsequent *in vitro* transcription of SpoX (CDIF630nc_038, short isoform).	this study
pFF-245	Derived from pSC-A-amp/kan for PCR amplification and subsequent *in vitro* transcription of SpoX* (CDIF630nc_038, short isoform with a mutated seed region).	this study
pFF-166	Derived from pSC-A-amp/kan for PCR amplification and subsequent *in vitro* transcription of *spo0A* (CDIF630_01363 5'UTR starting from primary TSS plus 84 nt of CDS).	this study
pFF-248	Derived from pSC-A-amp/kan for PCR amplification and subsequent *in vitro* transcription of *spo0A*^{*C} SpoY* (CDIF630_01363 5'UTR starting from primary TSS plus 69 nt of CDS with mutations compensating for the mutated SpoY seed region at the SpoY binding site).	this study
pFF-247	Derived from pSC-A-amp/kan for PCR amplification and subsequent *in vitro* transcription of *spo0A*^{*C} SpoX* (CDIF630_01363 5'UTR starting from primary TSS plus 69 nt of CDS with mutations compensating for the mutated SpoX seed region at the SpoX binding site).	this study
p JAK112	To generate gene deletions in *C. difficile* 630 by homologous recombination. Carrying *E. coli codA* for counterselection in *C. difficile*.	[2]
pFF-170	Derived from pJAK112 for deletion of SpoY (CDIF630nc_020).	this study

pFF-171	Derived from pJAK112 for deletion of SpoX (CDIF630nc_038).	this study
pDSW1728	To monitor gene expression with a codon-optimized variant of mCherry (mCherryOpt) in *C. difficile*. Designed for cloning a promoter of interest upstream of *mCherryOpt*.	[3]
pFF-185	*p*[*spo0A*] - Derived from pDSW1728 for constitutive expression of *spo0A* (CDIF630_01363 5'UTR starting from primary TSS) plus 60 nt of CDS fused to *mCherryOpt*, controlled by the *C. difficile* 630 *cwp2* promoter.	this study
pFF-186	*p*[SpoY] - Derived from pDSW1728 for constitutive expression of SpoY (CDIF630nc_020), controlled by the *C. difficile* 630 *fdxA* promoter.	this study
pFF-191	*p*[SpoY-*spo0A*] - Derived from pDSW1728 for constitutive co-expression of SpoY (CDIF630nc_020), controlled by the *C. difficile* 630 *fdxA* promoter, and *spo0A* (CDIF630_01363 5'UTR starting from primary TSS plus 60 nt of CDS) fused to *mCherryOpt*, controlled by the *C. difficile* 630 *cwp2* promoter.	this study
pFF-254	*p*[SpoY*-*spo0A*] - Derived from pDSW1728 for constitutive co-expression of SpoY* (CDIF630nc_020 with a mutated seed region), controlled by the *C. difficile* 630 *fdxA* promoter, and *spo0A* (CDIF630_01363 5'UTR starting from primary TSS plus 60 nt of CDS) fused to *mCherryOpt*, controlled by the *C. difficile* 630 *cwp2* promoter.	this study
pFF-285	*p*[SpoY*-*spo0A**^{C}] - Derived from pDSW1728 for constitutive co-expression of SpoY* (CDIF630nc_020 with a mutated seed region), controlled by the *C. difficile* 630 *fdxA* promoter, and *spo0A* (CDIF630_01363 5'UTR starting from primary TSS plus 60 nt of CDS with mutations compensating for the mutated SpoY seed region at the SpoY binding site) fused to *mCherryOpt*, controlled by the *C. difficile* 630 *cwp2* promoter.	this study
pFF-187	*p*[SpoX] - Derived from pDSW1728 for constitutive expression of SpoX (CDIF630nc_038, long isoform), controlled by the *C. difficile* 630 *fdxA* promoter.	this study
pFF-192	*p*[SpoX-*spo0A*] - Derived from pDSW1728 for constitutive co-expression of SpoX (CDIF630nc_038, long isoform), controlled by the *C. difficile* 630 *fdxA* promoter, and *spo0A* (CDIF630_01363 5'UTR starting from primary TSS plus 60 nt of CDS) fused to *mCherryOpt*, controlled by the *C. difficile* 630 *cwp2* promoter.	this study
pFF-260	*p*[SpoX*-*spo0A*] - Derived from pDSW1728 for constitutive co-expression of SpoX* (CDIF630nc_038, long isoform with a mutated seed region), controlled by the *C. difficile* 630 *fdxA* promoter, and *spo0A*	this study

	(CDIF630_01363 5'UTR starting from primary TSS plus 60 nt) of CDS fused to *mCherryOpt*, controlled by the *C. difficile* 630 *cwp2* promoter.	
pFF-289	*p*[SpoX*-$spo0A*^{C}$] - Derived from pDSW1728 for constitutive co-expression of SpoX* (CDIF630nc_038, long isoform with a mutated seed region), controlled by the *C. difficile* 630 *fdxA* promoter, and *spo0A* (CDIF630_01363 5'UTR starting from primary TSS plus 60 nt of CDS with mutations compensating for the mutated SpoX seed region at the SpoX binding site) fused to *mCherryOpt*, controlled by the *C. difficile* 630 *cwp2* promoter.	this study
pFF-207	*p*[ctl] - Derived from pDSW1728, empty control vector.	this study
pFF-167	Derived from pSC-A-amp/kan for PCR amplification and subsequent *in vitro* transcription of *spo0A* (CDIF630_01363 5'UTR starting from secondary TSS plus 84 nt of CDS).	this study
pFF-344	*p*[SpoY-*cwp2*] - Derived from pDSW1728 for constitutive co-expression of SpoY (CDIF630nc_020), controlled by the *C. difficile* 630 *fdxA* promoter, and *cwp2* (CDIF630_03054 5'UTR plus 75 nt of CDS) fused to *mCherryOpt*, controlled by the *C. difficile* 630 *cwp2* promoter.	this study
pFF-345	*p*[*cwp2*] - Derived from pDSW1728 for constitutive expression of *cwp2* (CDIF630_03054 5'UTR plus 75 nt) of CDS fused to *mCherryOpt*, controlled by the *C. difficile* 630 *cwp2* promoter.	this study
pFF-346	*p*[SpoX-*cwpV*] - Derived from pDSW1728 for constitutive co-expression of SpoX (CDIF630nc_038, long isoform), controlled by the *C. difficile* 630 *fdxA* promoter, and *cwpV* (CDIF630_00626 5'UTR plus 75 nt of CDS) fused to *mCherryOpt*, controlled by the *C. difficile* 630 *cwp2* promoter.	this study
pFF-347	*p*[*cwpV*] - Derived from pDSW1728 for constitutive expression of *cwpV* (CDIF630_00626 5'UTR plus 75 nt) of CDS fused to *mCherryOpt*, controlled by the *C. difficile* 630 *cwp2* promoter.	this study

Appendix Table S3: DNA oligonucleotides used in this study.

Oligo	Sequence (5'-3')	Purpose and Reference
Plasmid construction		
FFO-364	cgtagaaatacggtgtttttgttaccctaTTCTATGCAAATATATGAATATATGGATATTG	Amplification of *hfq* CDS and upstream region for Gibson assembly into pJAK184 – for insertion of an *hfq* C-terminal 3XFLAG tag.
FFO-365	atggtctttgtagtcTCTGTTGTTATTATTATTGTTGTTTTG	Amplification of *hfq* CDS and upstream region for Gibson assembly into pJAK184 – for insertion of an *hfq* C-terminal 3XFLAG tag.
FFO-368	gacgatgacaagtagATAATTAATTTAATTTAAGATGATTGAGAGG	Amplification of *hfq* CDS and downstream region for Gibson assembly into pJAK184 – for insertion of an *hfq* C-terminal 3XFLAG tag.
FFO-369	gggattttggtcatgagattatcaaaaaggTACATAAGAATCGACTGGTGC	Amplification of *hfq* CDS and downstream region for Gibson assembly into pJAK184 – for insertion of an *hfq* C-terminal 3XFLAG tag.
FFO-366	aataataacaacagaGACTACAAAGACCATGACGG	Amplification of 3XFLAG tag for Gibson cloning into pJAK184– for insertion of an *hfq* C-terminal 3XFLAG tag.
FFO-367	aattaaattaattatCTACTTGTCATCGTCATCCTTG	Amplification of 3XFLAG tag for Gibson cloning into pJAK184 – for insertion of an *hfq* C-terminal 3XFLAG tag.
FFO-362	CCTTTTTGATAATCTCATGACCAAAATC	Linearization of pJAK184 for insertion of homology arms[4].
FFO-363	TAGGGTAACAAAAACACCGTATTTC	Linearization of pJAK184 for insertion of homology arms[4].
FFO-958	GTTTTTTTTAATACGACTCACTATAGGGagatagtagattacaatgatttttg	Amplification of SpoY (CDIF630nc_020) for Strata cloning into pSC-A-amp/kan, adding a T7 promoter to the 5' end.

FFO-959	aaaaaaaagagacagccc	Amplification of SpoY (CDIF630nc_020) for Strata cloning into pSC-A-amp/kan, adding a T7 promoter to the 5' end.
FFO-960	AAAAAAAAGAGACAGCCCGTTTAAGAAGCT GTCATATATAATATGATAGTAG	Amplification and mutation (seed region) of SpoY (CDIF630nc_020), for Strata cloning into pSC-A-amp/kan, adding a T7 promoter to the 5' end.
FFO-961	GTTTTTTTTAATACGACTCACTATAGGGaata taaaataacaaacaaatcttaacaaaaaattaaac	Amplification of SpoX (CDIF630nc_038, short isoform) for Strata cloning into pSC-A-amp/kan, adding a T7 promoter to the 5' end.
FFO-962	aaaaataaagaaggcaacg	Amplification of SpoX (CDIF630nc_038, short isoform) for Strata cloning into pSC-A-amp/kan, adding a T7 promoter to the 5' end.
FFO-1261	GTTTTTTTTAATACGACTCACTATAGGGAAT ATAAAATAACAAACAAATCTTAACAAAAAA TTTTTAAAAATTTAT	Amplification and mutation (seed region) of SpoX (CDIF630nc_038, short isoform), for Strata cloning into pSC-A-amp/kan, adding a T7 promoter to the 5' end.
FFO-1262	AAAAATAAAGAAGGCAACGGGAAGCCTTCT TTCATATAAATTTTTAAAAA	Amplification and mutation (seed region) of SpoX (CDIF630nc_038, short isoform), for Strata cloning into pSC-A-amp/kan, adding a T7 promoter to the 5' end.
FFO-964	GTTTTTTTTAATACGACTCACTATAGGGgagg cattaaaaaattttattattttatc	Amplification of 5'UTR (pTSS) and start of CDS (69 nt) of *spo0A* for Strata cloning into pSC-A-amp/kan, adding a T7 promoter to the 5' end.
FFO-965	GTTTTTTTTAATACGACTCACTATAGGGgagt agataattaggaagcaattg	Amplification of 5'UTR (sTSS) and start of CDS (69 nt) of *spo0A* for Strata cloning into pSC-A-amp/kan, adding a T7 promoter to the 5' end.
FFO-966	caaatactcttttaatacctgac	Amplification of 5'UTR (pTSS) and start of CDS (69 nt) of *spo0A* for Strata cloning into pSC-A-amp/kan, adding a T7 promoter to the 5' end.

FFO-1268	taaaaatcatatcattaaaaaacatcttcttattacag	To insert compensatory mutations at the SpoY target site in *spo0A* (pTSS only).
FFO-1269	ttttttaatgatatgatttttagtggaaaaaatcaaaatag	To insert compensatory mutations at the SpoY target site in *spo0A* (pTSS only).
FFO-1259	tttaaaaatatataattgcttcctaattatc	To insert compensatory mutations at the SpoX target site in *spo0A* (pTSS only).
FFO-1267	atatatttttaaaagttttctgtaataagaag	To insert compensatory mutations at the SpoX target site in *spo0A* (pTSS only).
M13 rev	CAGGAAACAGCTATGAC	Amplification of fragments inserted in pSC-A-amp/kan.
M13 fwd	GTAAAACGACGGCCAGT	Amplification of fragments inserted in pSC-A-amp/kan.
FFO-977	ACCCTAGAGCTCgagcatggtttaataaattagaaaatg	Amplification of 1.2 kb homology arm upstream of SpoY (CDIF630nc_020) deletion region for insertion into pJAK112, SacI site.
FFO-978	ttaagaagctgtcattctactatctatatattattatacgatactactttttatatg	Amplification of 1.2 kb homology arm upstream of SpoY (CDIF630nc_020) deletion region for insertion into pJAK112, SacI site.
FFO-979	tatatagatagtagaatgacagcttcttaaacggg	Amplification of 1.2 kb homology arm downstream of SpoY (CDIF630nc_020) deletion region for insertion into pJAK112, BamHI site.
FFO-980	AAAAGGGGATCCaaagtatctatcaactctttatcaaaaag	Amplification of 1.2 kb homology arm downstream of SpoY (CDIF630nc_020) deletion region for insertion into pJAK112, BamHI site.
FFO-985	ACCCTAGAGCTCtagtaagaggagacagagaaaaag	Amplification of 1.2 kb homology arm upstream of SpoX (CDIF630nc_038) deletion region for insertion into pJAK112, SacI site.

FFO-986	gaccagttgtgcaaaataaaaaaataagctgtttctaaaatgatttc	Amplification of 1.2 kb homology arm upstream of SpoX (CDIF630nc_038) deletion region for insertion into pJAK112, SacI site.
FFO-987	agcttatttttttattttgcacaactggtcattattaatg	Amplification of 1.2 kb homology arm downstream of SpoX (CDIF630nc_038) deletion region for insertion into pJAK112, BamHI site.
FFO-988	AAAAGGGGATCCctctctattcatgcacaaaattg	Amplification of 1.2 kb homology arm downstream of SpoX (CDIF630nc_038) deletion region for insertion into pJAK112, BamHI site.
FFO-1004	CATCAAGCTAGCaaaagtttatatcttttggttaattattacaataag	Amplification of the *cwp2* promoter (80 nt upstream of TSS), inserting a NheI restriction site at the 5' end.
FFO-1000	attttttaatgcctcctcgagttaccaattataatatatatttgatattatttc	Amplification of the *cwp2* promoter (80 nt upstream of TSS), inserting a XhoI restriction site at the 3' end.
FFO-1001	aattggtaactcgaggaggcattaaaaaatttttattattttatcaattatc	Amplification of 5'UTR (pTSS) and start of CDS (60 nt) of *spo0A*, inserting a XhoI restriction site at the 5' end.
FFO-1002	aatatcttcagatccaaatgctacatgttcgagctcttttaatacctgacaaaaatc	Amplification of 5'UTR (pTSS) and start of CDS (60 nt) of *spo0A*, inserting a SacI restriction site at the 3' end.
FFO-1056	ttaaaagagctcgtatctaaaggagaagaagataatatg	Amplification of *mCherryOpt*, inserting a SacI restriction site directly upstream of the second codon in the *mCherryOpt* CDS.
FFO-1057	cttataggatccttatttatataattcatccatacctcc	Amplification of *mCherryOpt*.
FFO-995	catcaagctagcaacaagaatatcataataaagtttttgttg	Amplification of the *fdxA* promoter (80 nt upstream of TSS), inserting a NheI restriction site at the 5' end.
FFO-1005	tgtaatctactatctcaataacattataacaaatattattgaatataacaattaaattaattc	Amplification of the *fdxA* promoter (80 nt upstream of TSS), inserting a SpoY overlapping region at the 3'end.

FFO-1006	gttataatgttattgagatagtagattacaatgatttttgtac	Amplification of SpoY (CDIF630nc_020), inserting a *fdxA* overlapping region at 5' end.
FFO-1007	CTTATAGGATCCaaaaaagacttctcatgagagaagc cttttttctagaaaaaaaaagagacagcccg	Amplification of SpoY (CDIF630nc_020), inserting a XbaI restriction site, *slpA* terminator and BamHI restriction site at 3' end.
FFO-999	tctagaaaaaaggcttctctcatgagaagtctttttaaaagt ttatatcttttggttaattattacaataag	Amplification of the *cwp2* promoter (80 nt upstream of TSS) and 5'UTR (pTSS) and start of CDS (60 nt) of *spo0A* fused to *mCherryOpt* from pFF-185, exchanging the NheI restriction site for a XbaI restriction site and *slpA* terminator at the 5' end.
FFO-1057	cttataggatccttatttatataattcatccatacctcc	Amplification of the *cwp2* promoter (80 nt upstream of TSS) and 5'UTR (pTSS) and start of CDS (60 nt) of *spo0A* fused to *mCherryOpt* from pFF-185, including the BamHI restriction site at the 3' end.
FFO-1008	ttgttattttatattcaataacattataacaaatattattgaata taacaattaaattaattc	Amplification of the *fdxA* promoter (80 nt upstream of TSS), inserting a SpoX overlapping region at the 3'end.
FFO-1009	gttataatgttattgaatataaaataacaaacaaatcttaaca aaaaattaaac	Amplification of SpoX (CDIF630nc_038), inserting a *fdxA* overlapping region at 5' end.
FFO-1010	cttttttctagataaatatagaaagaactagcttaaaacataa tataattac	Amplification of SpoX (CDIF630nc_038, long isoform), inserting a XbaI restriction site at the 3' end.
FFO-1264	GTTATAATGTTATTGAATATAAAATAACAA ACAAATCTTAACAAAAAATTTTTAAAAATT TAT	Amplification and mutation (seed region) of SpoX (CDIF630nc_038), inserting a *fdxA* overlapping region at the 5' end.
FFO-1263	gccttctttatttttataaaaaaataagctgtttctaaaatg	To mutate the SpoX seed region (CDIF630nc_038).
FFO-994	CTTGTTgctagcttgatgcagaattc	To linearize pDSW1728 products, starting at the NheI restriction site.

FFO-1205	catcaagctagctataagttttaataaaactttaaatagaaaaagg	To linearize pDSW1728 products downstream of the BamHI restriction site, inserting an additional NheI restriction site at the 5 ’end.
FFO-1354	tggtaactcgagaataaggaaaaaataataaaaatttgaattttttaggg	Amplification of 5'UTR and start of CDS (75 nt) of CDIF630_03054 (*cwp2*), inserting a XhoI restriction site at the 5’ end.
FFO-1355	agatacgagctctgcagcaaaaactggagc	Amplification of 5'UTR and start of CDS (75 nt) of CDIF630_03054 (*cwp2*), inserting a SacI restriction site at the 3’ end.
FFO-1469	tggtaactcgagataaataataaaatttatttgtaaaaagagtagcac	Amplification of 5'UTR and start of CDS (75 nt) of CDIF630_00626 (*cwpV*) , inserting a XhoI restriction site at the 5’ end.
FFO-1470	agatacgagctcagctatgcctgctgttgaac	Amplification of 5'UTR and start of CDS (75 nt) of CDIF630_00626 (*cwpV*), inserting a SacI restriction site at the 3’ end.
Northern blot probes		
FFO-942	CAATTTTCAAAGGGTTAGGG	Targeting CDIF630nc_152.
FFO-943	AATCACCCAAACGCCAATAA	Targeting CDIF630nc_153.
FFO-944	AAATGGGGGAGATTGAGTAT	Targeting CDIF630nc_155.
FFO-947	AAAAAAGCACTCCCCCAGCA	Targeting CDIF630nc_164.
FFO-948	TTATAAGGAGTGCTTTGGTG	Targeting CDIF630nc_165.
FFO-951	GGCTCGATTCAGAAAATAT	Targeting CDIF630nc_171.
FFO-317	TAAGAAGCTGTCATATATAGGGGGA	Targeting SpoY (CDIF630nc_020), WT only.
FFO-1014	TTTGTTAAGATTTGTTTGTT	Targeting SpoX (CDIF630nc_038).

FFO-352	GAAACAGCCAAGTTATTCTA	Targeting CDIF630nc_037.
FFO-1507	AAATAAAGAAGGCAACGGGAAGC	Targeting SpoX (CDIF630nc_038), short isoform only.
FFO-1550	CCCGTTTAAGAAGCTGTCAT	Targeting SpoY (CDIF630nc_020), WT and seed region mutant.
CD76	TCAGCGCTAGAGAGCTTAAC	Targeting 5S rRNA[2].
RT-qPCR primer		
FFO-1421	ATGGGGGGATTTTTAGTGG	*spo0A*, 5' end[5]
FFO-1422	TCATTTGAGTCTCTTGAACTGGTC	*spo0A*, 3' end[5]
FFO-1423	GTTGGTTATGGCACTTGACAG	*sigE*, 5' end[5]
FFO-1424	GACTGTGATATTCCAAGC	*sigE*, 3' end[5]
FFO-1425	CAAGCAATTTAGGTCTAGTTAGGAGC	*sigF*, 5' end[5]
FFO-1426	AAGCTTCACTTTCCATCTTTGCC	*sigF*, 3' end[5]
FFO-1427	GGATAGAACAAGAGATGAGATACCC	*spoIVA*, 5' end[6]
FFO-1428	CTGCTGCCTTTTCAAATGTC	*spoIVA*, 3' end[6]
FFO-1429	GATGCTATCCCTACTGCAACG	*spoIIQ*, 5' end[6]
FFO-1430	GTCCTTCTGTTACCTTCTGTTC	*spoIIQ*, 3' end[6]
FFO-1431	TGGTACAGAGGCTAACTATGTTCTTG	*sigK*, 5' end[7]
FFO-1432	CTGGACAATTTCTCTTTCTCTAGG	*sigK*, 3' end[7]
FFO-1433	GTGGTGTTAATACATCAGAACTTCC	*sigG*, 5' end[5]
FFO-1434	GTTGAAAACCTTACATTTTGGC	*sigG*, 3' end[5]

FFO-1437	ACAGAACAGTAGTACCAGG	*sspA*, 5' end[5]
FFO-1438	CTATCTGTTGCTTTTTCCAGC	*sspA*, 3' end[5]

SUPPLEMENTARY REFERENCES

1. Mann, M., Wright, P. R. & Backofen, R. IntaRNA 2.0: enhanced and customizable prediction of RNA–RNA interactions. *Nucleic Acids Res.* **45**, W435–W439 (2017).

2. Fuchs, M. *et al.* An RNA-centric global view of Clostridioides difficile reveals broad activity of Hfq in a clinically important gram-positive bacterium. *Proc. Natl. Acad. Sci.* **118**, (2021).

3. Ransom, E. M., Ellermeier, C. D. & Weiss, D. S. Use of mCherry red fluorescent protein for studies of protein localization and gene expression in Clostridium difficile. *Appl. Environ. Microbiol.* **81**, 1652–1660 (2015).

4. Cartman, S. T., Kelly, M. L., Heeg, D., Heap, J. T. & Minton, N. P. Precise manipulation of the Clostridium difficile chromosome reveals a lack of association between the tcdC genotype and toxin p roduction. *Appl. Environ. Microbiol.* **78**, 4683–4690 (2012).

5. Oliveira, P. H. *et al.* Epigenomic characterization of Clostridioides difficile finds a conserved DNA methyltransferase that mediates sporulation and pathogenesis. *Nat. Microbiol.* **5**, 166–180 (2020).

6. Fimlaid, K. A. *et al.* Global analysis of the sporulation pathway of Clostridium difficile. *PLoS Genet.* **9**, e1003660 (2013).

7. Saujet, L. *et al.* Genome-wide analysis of cell type-specific gene transcription during spore formation in Clostridium difficile. *PLoS Genet.* **9**, e1003756 (2013).
