# Supplementary material for: A network of small RNAs regulates sporulation initiation in Clostridioides difficile
Source: EMBO J. 2023 May 4;42(12):e112858. doi: 10.15252/embj.2022112858 (PMC10267692; doi:10.15252/embj.2022112858)
Supplement: Supplementary file 2 — Expanded View Figures PDF [file EMBJ-42-e112858-s011.pdf]

Expanded View Figures

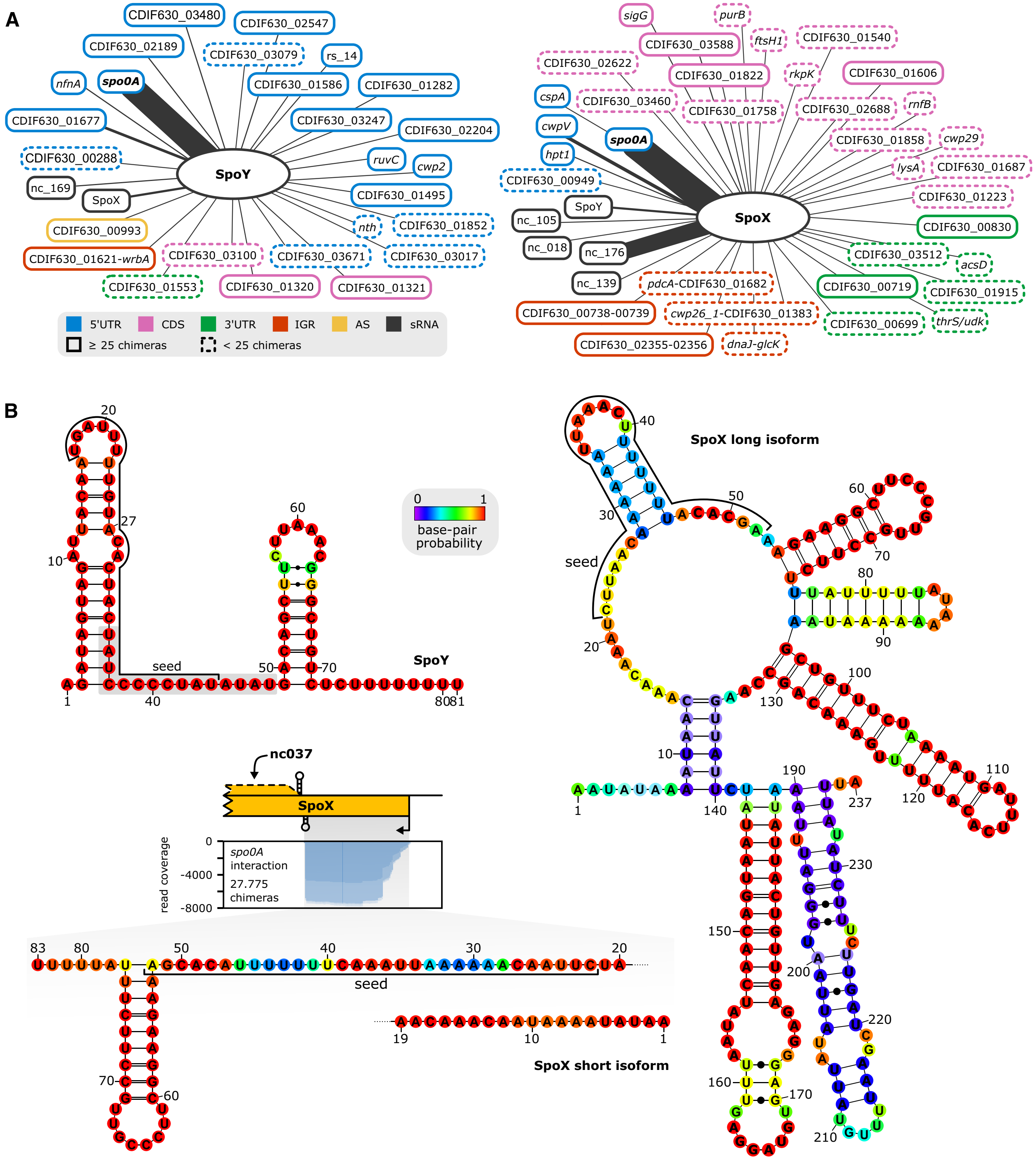

Figure EV1.

◀ **Figure EV1. RIL-seq reveals *spo0A* as a target of sRNA-mediated post-transcriptional regulation.**

A Target network of SpoY and SpoX, targets supported by ≥ 25 chimeras are marked by a solid line, while targets supported by < 25 chimeras are highlighted with a dashed line. Target types are discriminated by color. Edge strength correlates with the number of chimeras supporting an individual interaction.

B Predicted secondary structure (RNAfold; Lorenz *et al*, 2011) for SpoY and both isoforms of SpoX are provided. Seed regions relevant for *spo0A* interaction were predicted *in silico* (IntaRNA; Mann *et al*, 2017) and emphasized in the secondary structure. Read coverage of SpoX by SpoX-*spo0A* chimeric reads is highlighted in relation to the SpoX-encoding region.

 © 2023 The Authors

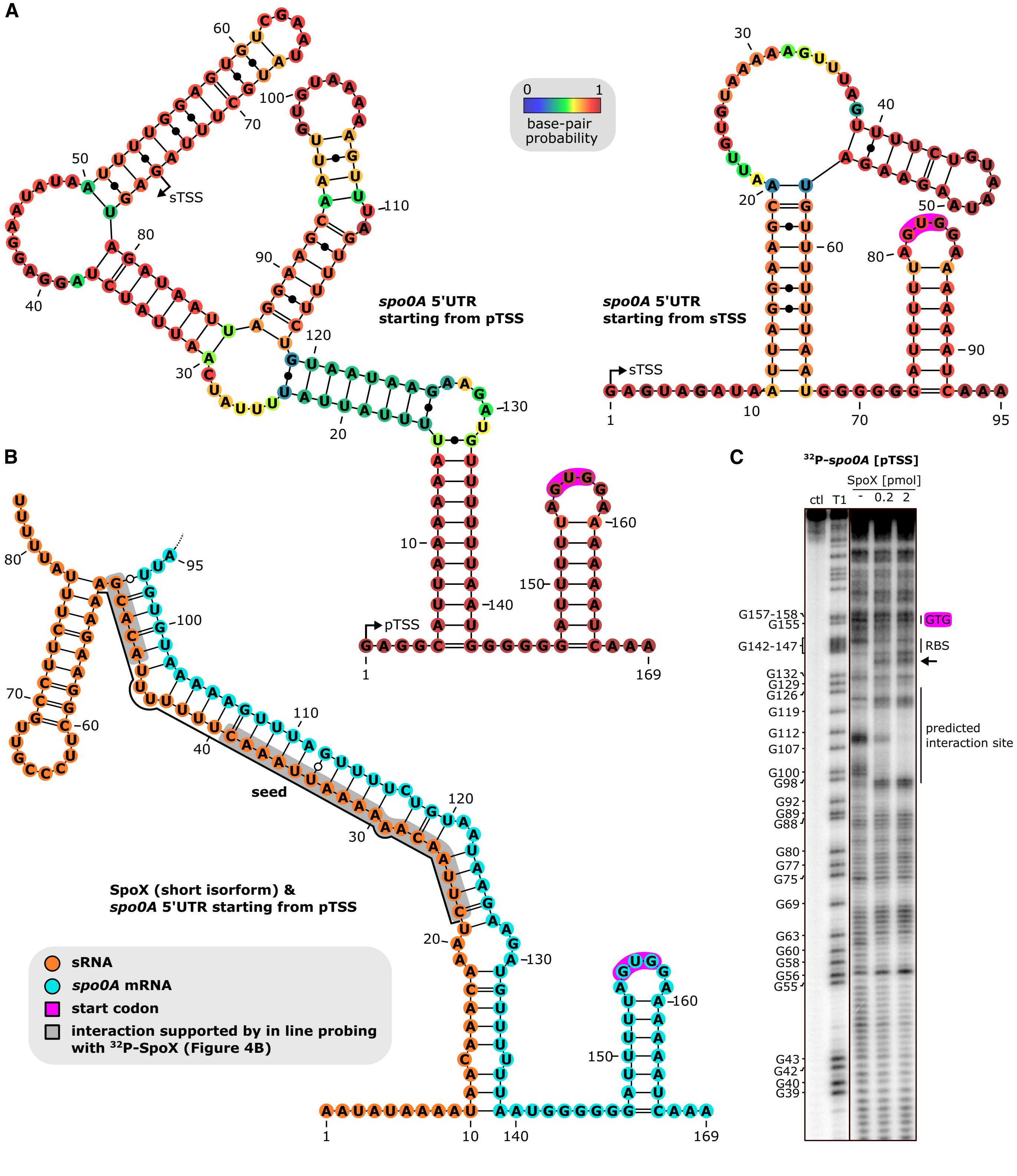
A
0
1
base-pair probability
sTSS
spo0A 5'UTR starting from pTSS
spo0A 5'UTR starting from sTSS
pTSS
B
seed
SpoX (short isorform) & spo0A 5'UTR starting from pTSS
sRNA
spo0A mRNA
start codon
interaction supported by in line probing with ³²P-SpoX (Figure 4B)
C
³²P-spo0A [pTSS]
SpoX [pmol]
ctl
T1
-
0.2
2
GTG
RBS
predicted interaction site
G157-158
G155
G142-147
G132
G129
G126
G119
G112
G107
G100
G98
G92
G89
G88
G80
G77
G75
G69
G63
G60
G58
G56
G55
G43
G42
G40
G39

Figure EV2.

◀

Figure EV2. SpoX binding potentially renders the *spo0A* RBS region more accessible.

A Predicted secondary structures of the *spo0A* 5′UTR and beginning of CDS, starting from the primary transcription start site (pTSS) and secondary TSS (RNAfold; Lorenz *et al*, 2011). The start codon is highlighted in pink.

B Predicted secondary structures of the *spo0A* 5′UTR and beginning of CDS upon dimer formation with SpoX (shown is the short isoform). Resulting dimer for the depicted region looks identical, independent of which SpoX isoform or *spo0A* 5′UTR length was used (RNAcofold; Bernhart *et al*, 2006). sRNA and mRNA are highlighted in orange and blue, respectively. sRNA-target base-pairings supported by in-line probing (Fig 4B) are shaded in gray.

C In-line probing of 0.2 pmol of ^{32}P-labeled *spo0A* (starting from pTSS) in the absence (lane 3) or presence of increasing concentrations (lane 4&5) of SpoX (short isoform). RNase T1 digested *spo0A* serves as a ladder. Start codon and predicted seed region are highlighted. A representative image of three independent experiments is shown.

Figure EV3. *spo0A* is a target of extensive sRNA-mediated post-transcriptional regulation. ▶

On the left site, read coverage (y-axis) of *spo0A* by chimeric reads of all sRNA-*spo0A* interactions detected by RIL-seq analysis is depicted. The *spo0A* 5′UTR position including pTSS (sigA) and sTSS (sigH), start codon and coding sequence are marked on the x-axis. Chimeric reads covering *spo0A* were predominantly found at position 1 (RNA1) in a chimera and are color-coded in red. Chimeric reads found at position 2 (RNA2) are marked in blue. The number of chimeric reads covering each interaction is provided on the left. On the right site, base pairing information and location of the predicted binding sites (IntaRNA; Mann *et al*, 2017) for each interaction are highlighted. *In silico* predictions that do not overlap with RIL-seq data are marked in red, and location of the interaction in relation to the RIL-seq peak is indicated by an arrow. Predicted interactions overlapping with RIL-seq data are shaded in gray and superimposed over the coverage plots. The *spo0A* nucleotide position is calculated relative to the *spo0A* start codon (highlighted in pink).

 © 2023 The Authors

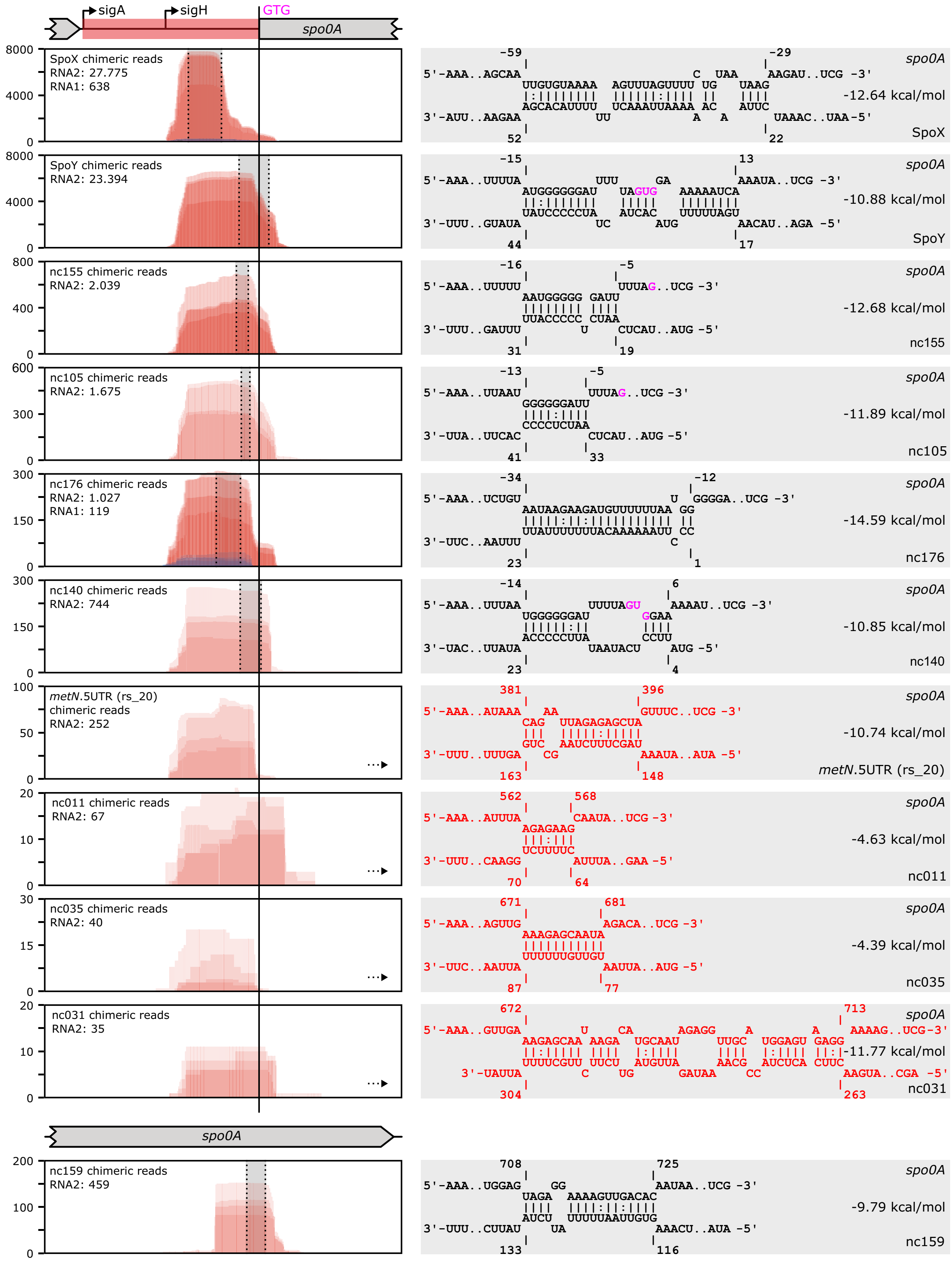
sigA
sigH
GTG
spo0A
SpoX chimeric reads
RNA2: 27.775
RNA1: 638
SpoY chimeric reads
RNA2: 23.394
nc155 chimeric reads
RNA2: 2.039
nc105 chimeric reads
RNA2: 1.675
nc176 chimeric reads
RNA2: 1.027
RNA1: 119
nc140 chimeric reads
RNA2: 744
metN.5UTR (rs_20) chimeric reads
RNA2: 252
nc011 chimeric reads
RNA2: 67
nc035 chimeric reads
RNA2: 40
nc031 chimeric reads
RNA2: 35
nc159 chimeric reads
RNA2: 459
-12.64 kcal/mol
SpoX
-10.88 kcal/mol
SpoY
-12.68 kcal/mol
nc155
-11.89 kcal/mol
nc105
-14.59 kcal/mol
nc176
-10.85 kcal/mol
nc140
-10.74 kcal/mol
metN.5UTR (rs_20)
-4.63 kcal/mol
nc011
-4.39 kcal/mol
nc035
-11.77 kcal/mol
nc031
-9.79 kcal/mol
nc159

Figure EV3.

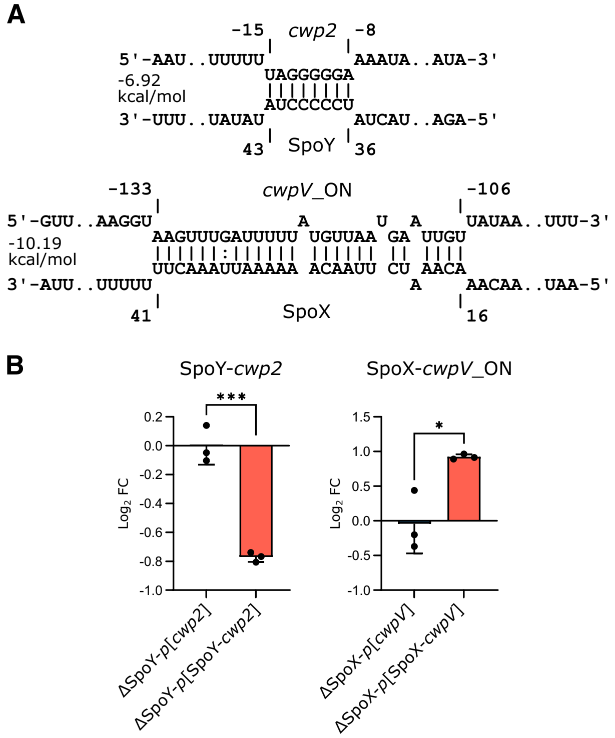

Figure EV4. SpoY and SpoX target additional genes besides *spo0A*.

A Base pairing information and location of the predicted binding sites (IntaRNA; Mann *et al*, 2017) for SpoY-*cwp2* and SpoX-*cwpV* with the invertible region within the *cwpV* 5′UTR in the ON orientation, allowing CwpV expression (Emerson *et al*, 2009). The nucleotide positions for *cwpV* and *cwp2* are calculated relative to the respective start codons.

B mCherry fluorescence of translational fusion constructs (error bars represent the mean ± SD of $n = 3$ biological replicates, Appendix Fig S5A) expressed in the respective sRNA knockout background. Fluorescence intensity was normalized to that of the respective *p*[*cwp2*]/ *p*[*cwpV*] ctl. Unpaired *t*-test was used to calculate statistical significance. Not significant (ns) $P > 0.05$; (*) $P \leq 0.05$; (***) $P \leq 0.001$.

© 2023 The Authors
