# Supplementary figures and images for: A network of small RNAs regulates sporulation initiation in Clostridioides difficile
Source: EMBO J. 2023 May 4;42(12):e112858. doi: 10.15252/embj.2022112858 (PMC10267692; doi:10.15252/embj.2022112858)

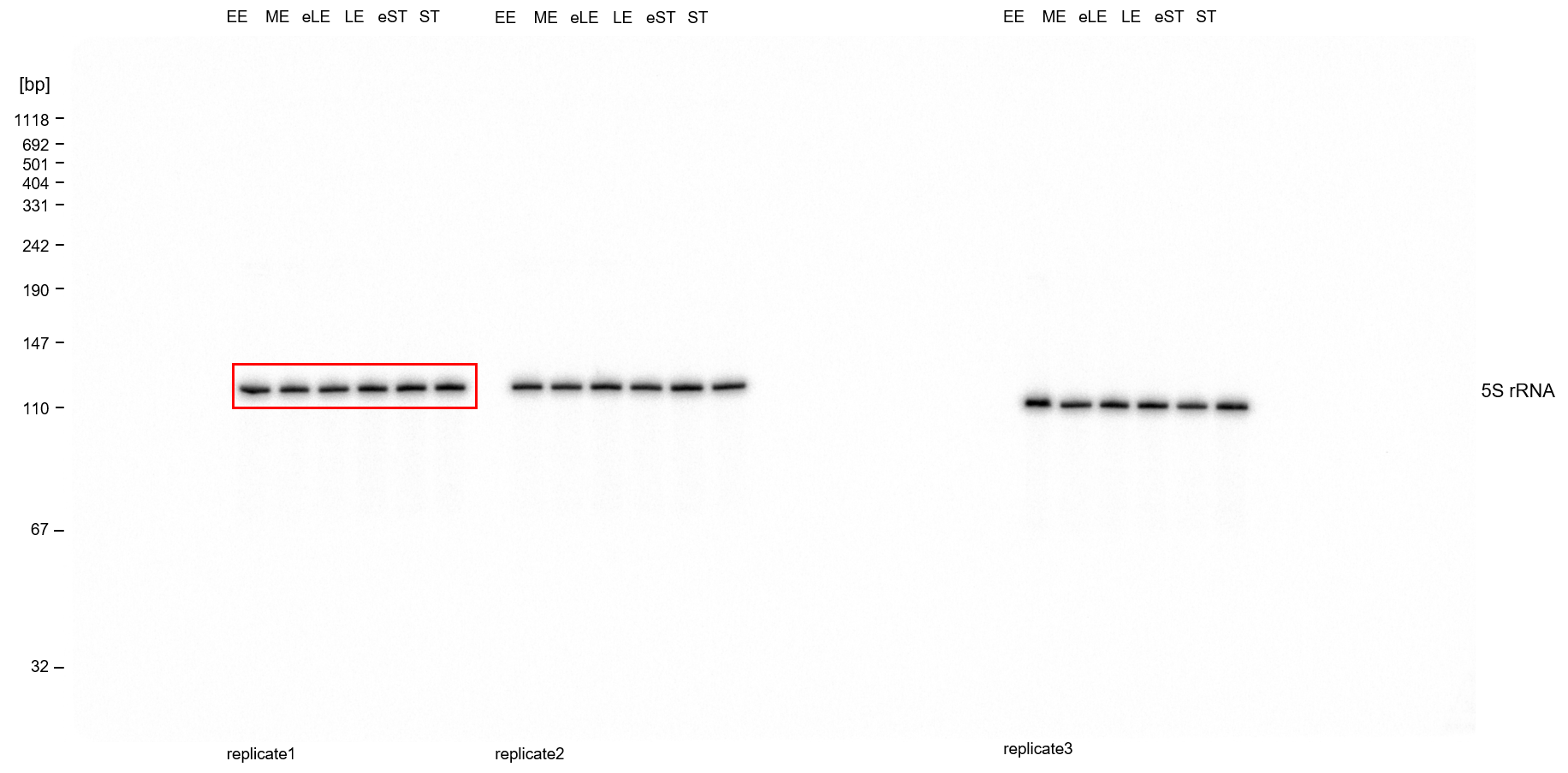

Supplement: Supplementary file 9 — Source Data for Figure 2 [file EMBJ-42-e112858-s004.zip › Figure 2/2C/2C-NB-5S_replicate1-3.tif]

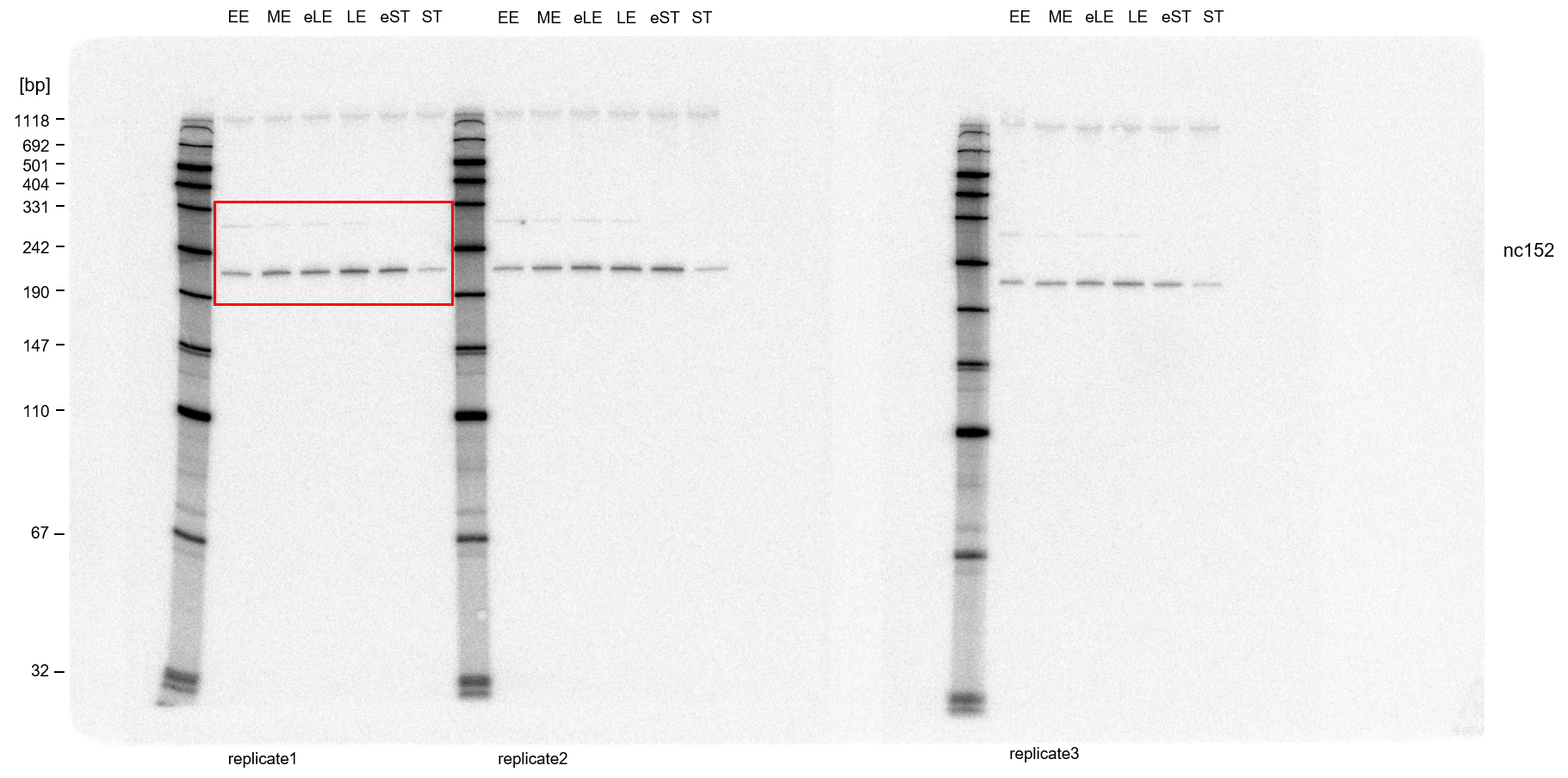

Supplement: Supplementary file 9 — Source Data for Figure 2 [file EMBJ-42-e112858-s004.zip › Figure 2/2C/2C-NB-nc152_replicate1-3.tif]

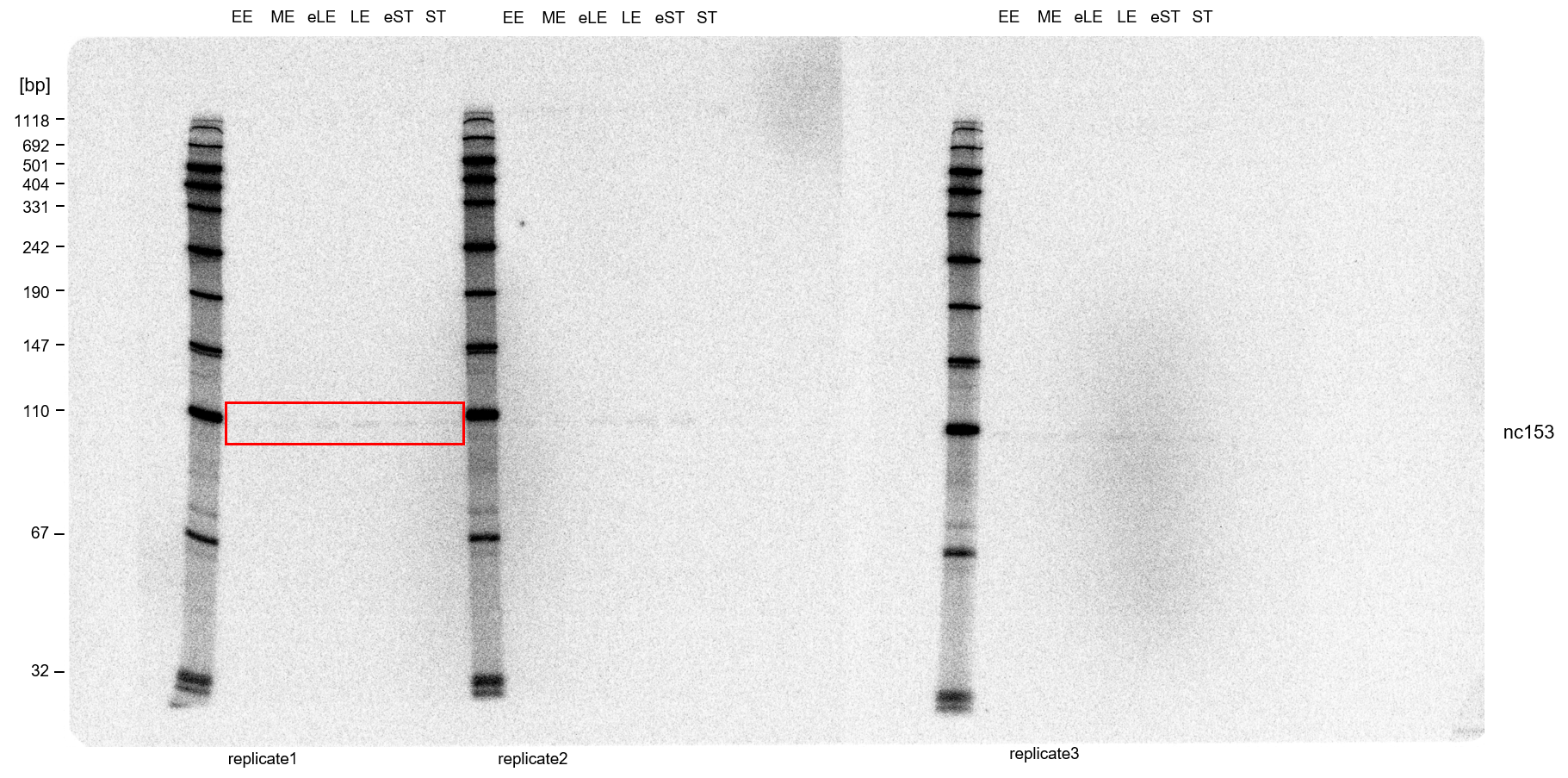

Supplement: Supplementary file 9 — Source Data for Figure 2 [file EMBJ-42-e112858-s004.zip › Figure 2/2C/2C-NB-nc153_replicate1-3.tif]

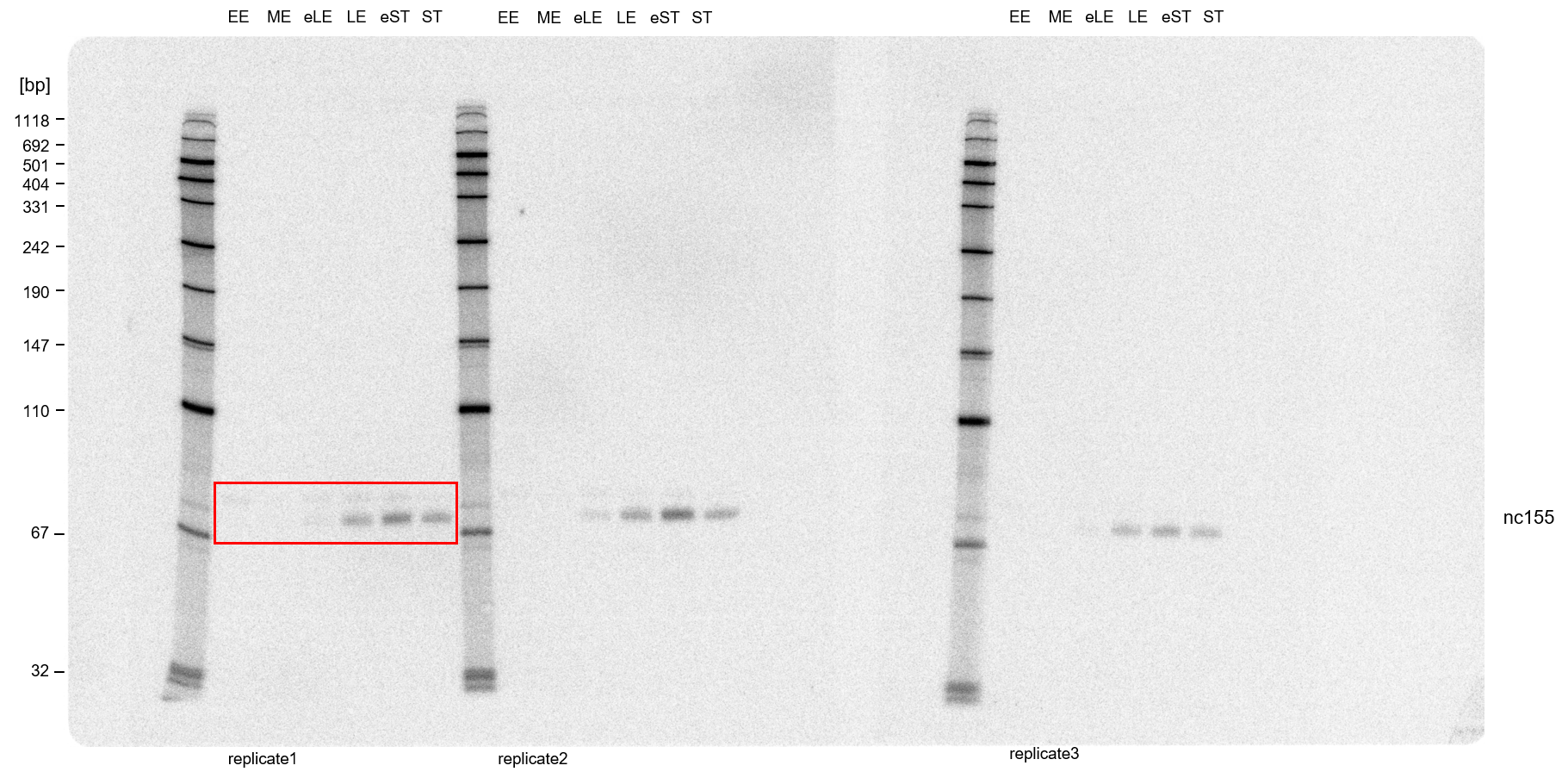

Supplement: Supplementary file 9 — Source Data for Figure 2 [file EMBJ-42-e112858-s004.zip › Figure 2/2C/2C-NB-nc155_replicate1-3.tif]

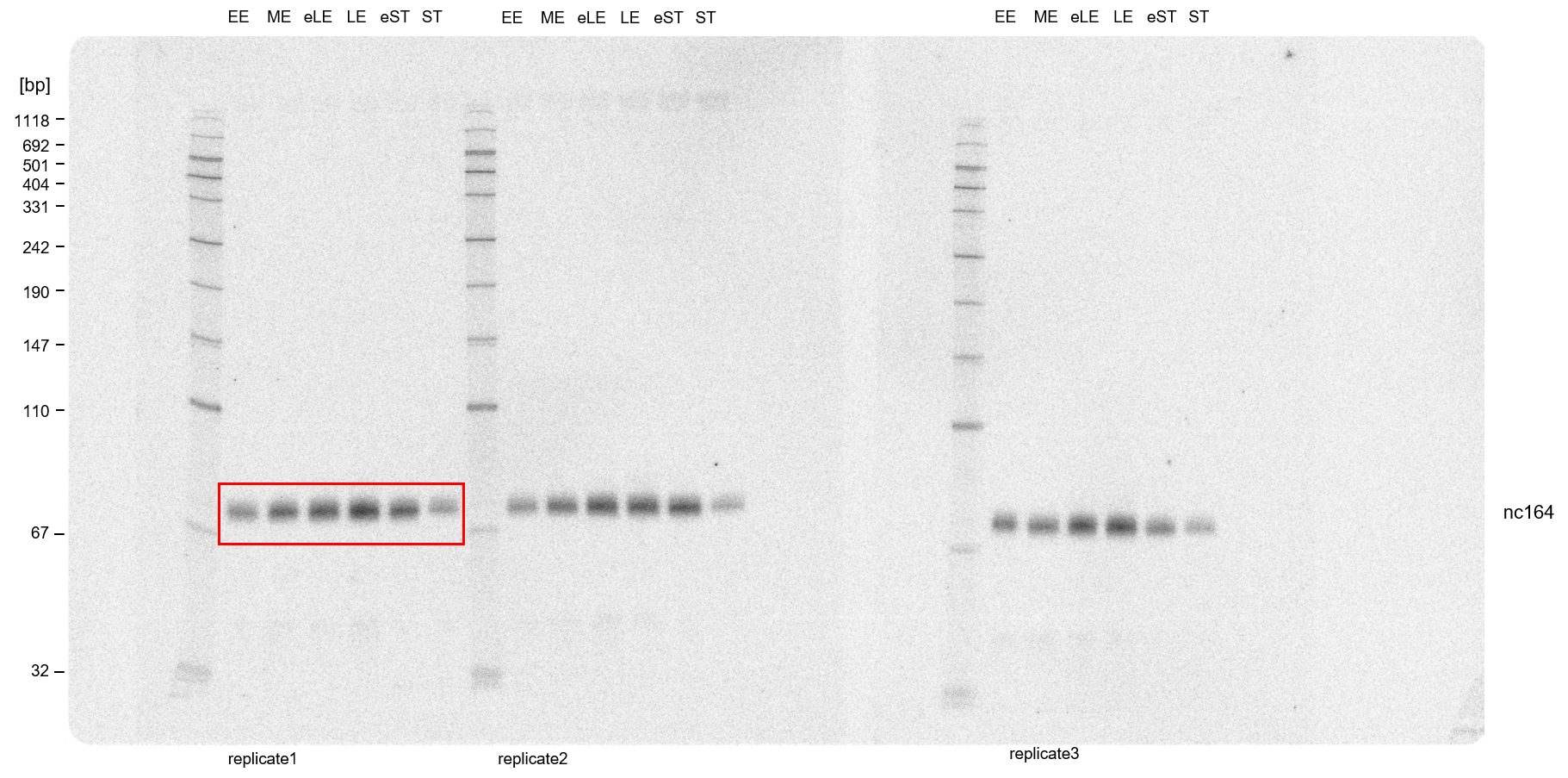

Supplement: Supplementary file 9 — Source Data for Figure 2 [file EMBJ-42-e112858-s004.zip › Figure 2/2C/2C-NB-nc164_replicate1-3.tif]

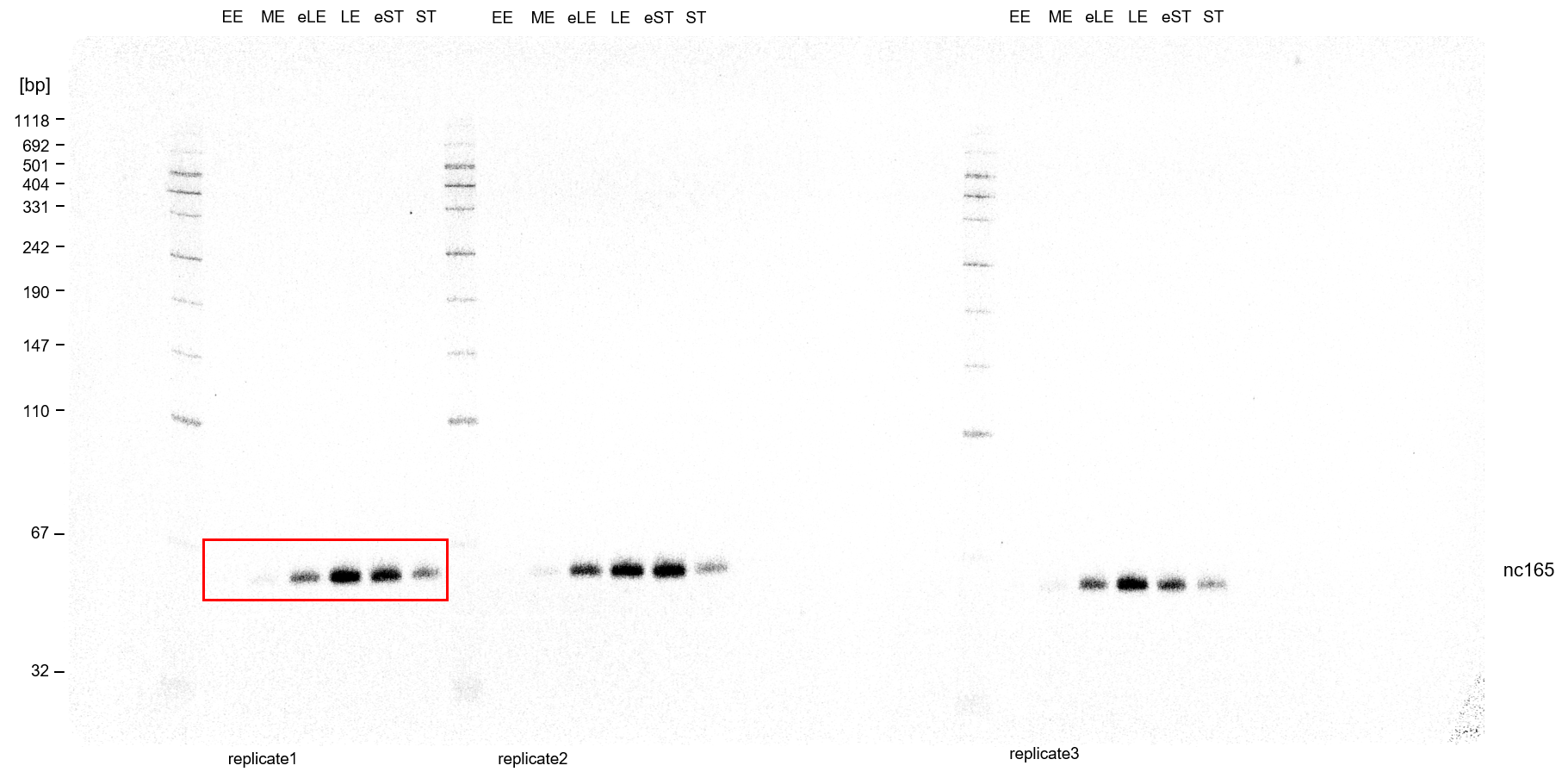

Supplement: Supplementary file 9 — Source Data for Figure 2 [file EMBJ-42-e112858-s004.zip › Figure 2/2C/2C-NB-nc165_replicate1-3.tif]

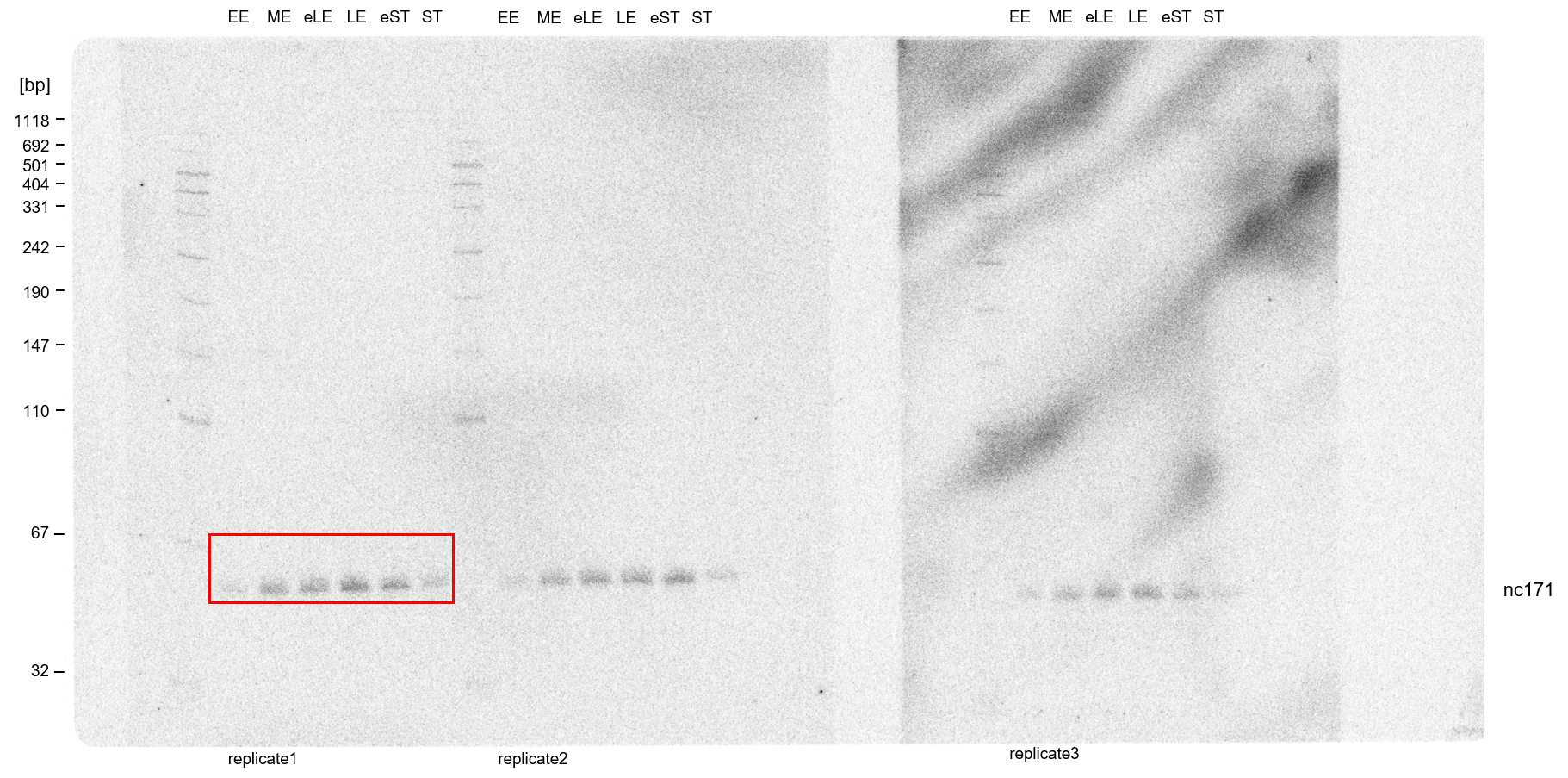

Supplement: Supplementary file 9 — Source Data for Figure 2 [file EMBJ-42-e112858-s004.zip › Figure 2/2C/2C-NB-nc171_relicate1-3.tif]

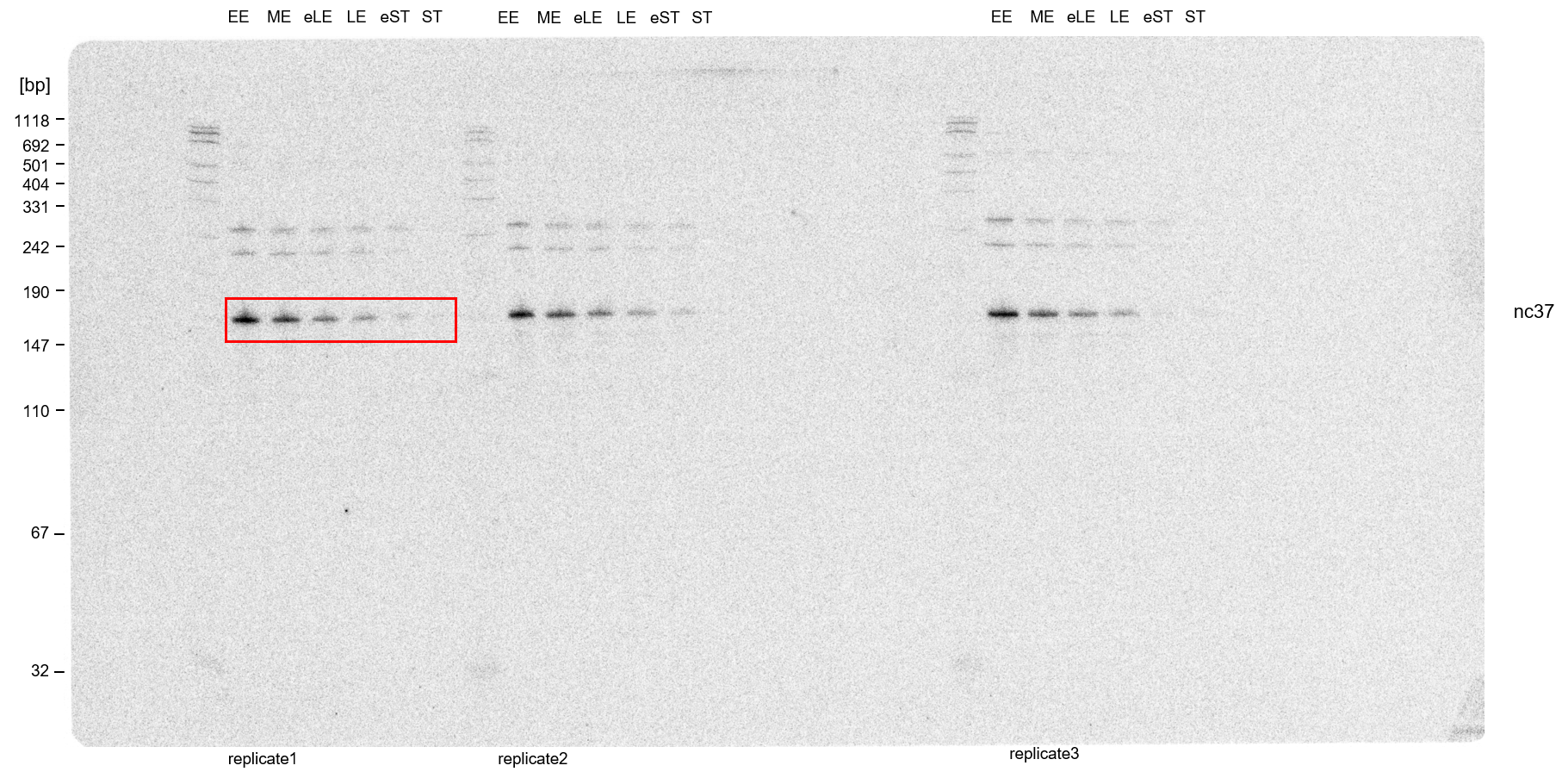

Supplement: Supplementary file 10 — Source Data for Figure 3 [file EMBJ-42-e112858-s015.zip › Figure 3/3C/3C-NB-nc037_replicate1-3.tif]

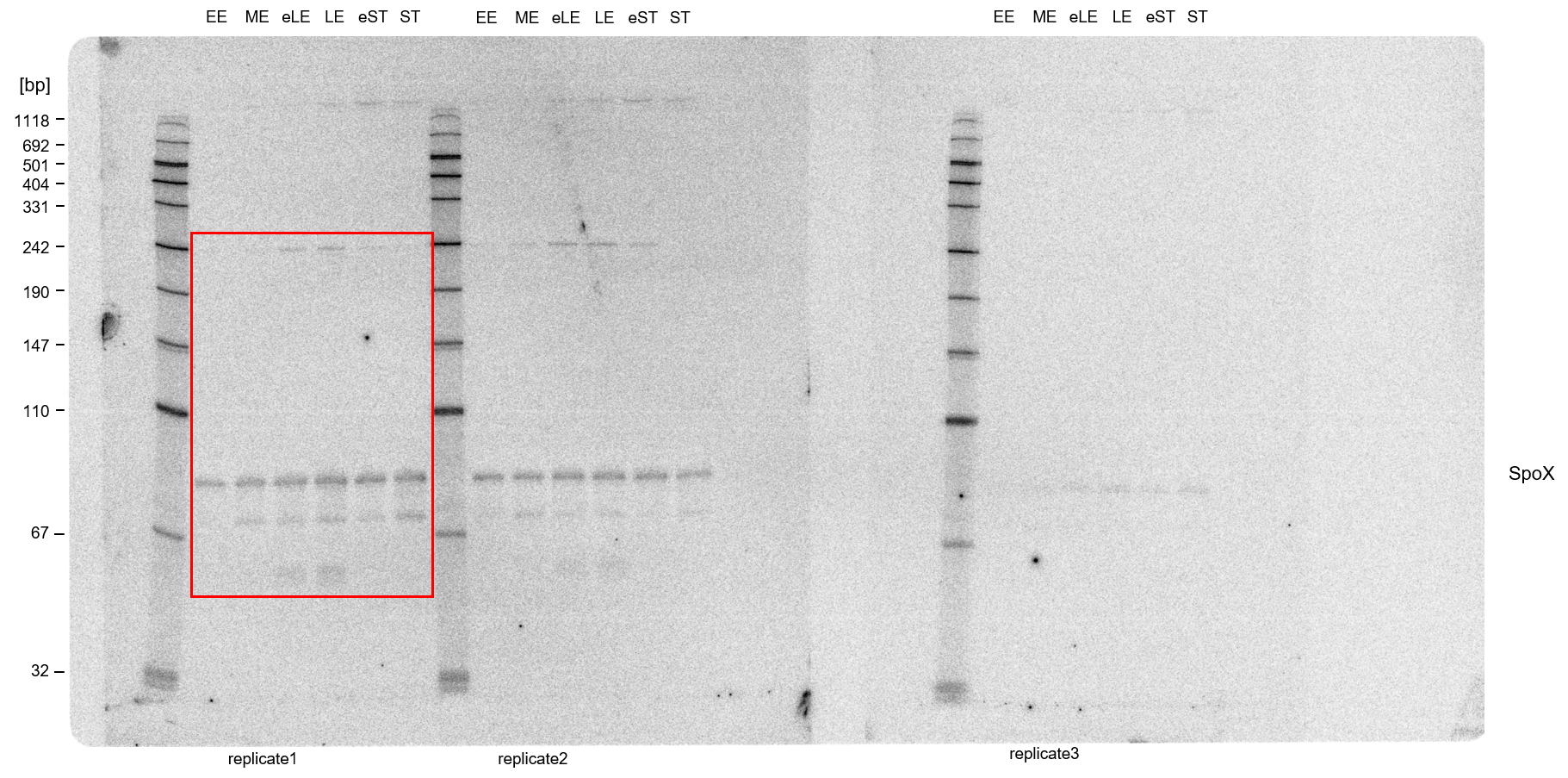

Supplement: Supplementary file 10 — Source Data for Figure 3 [file EMBJ-42-e112858-s015.zip › Figure 3/3C/3C-NB-SpoX_replicate1-3.tif]

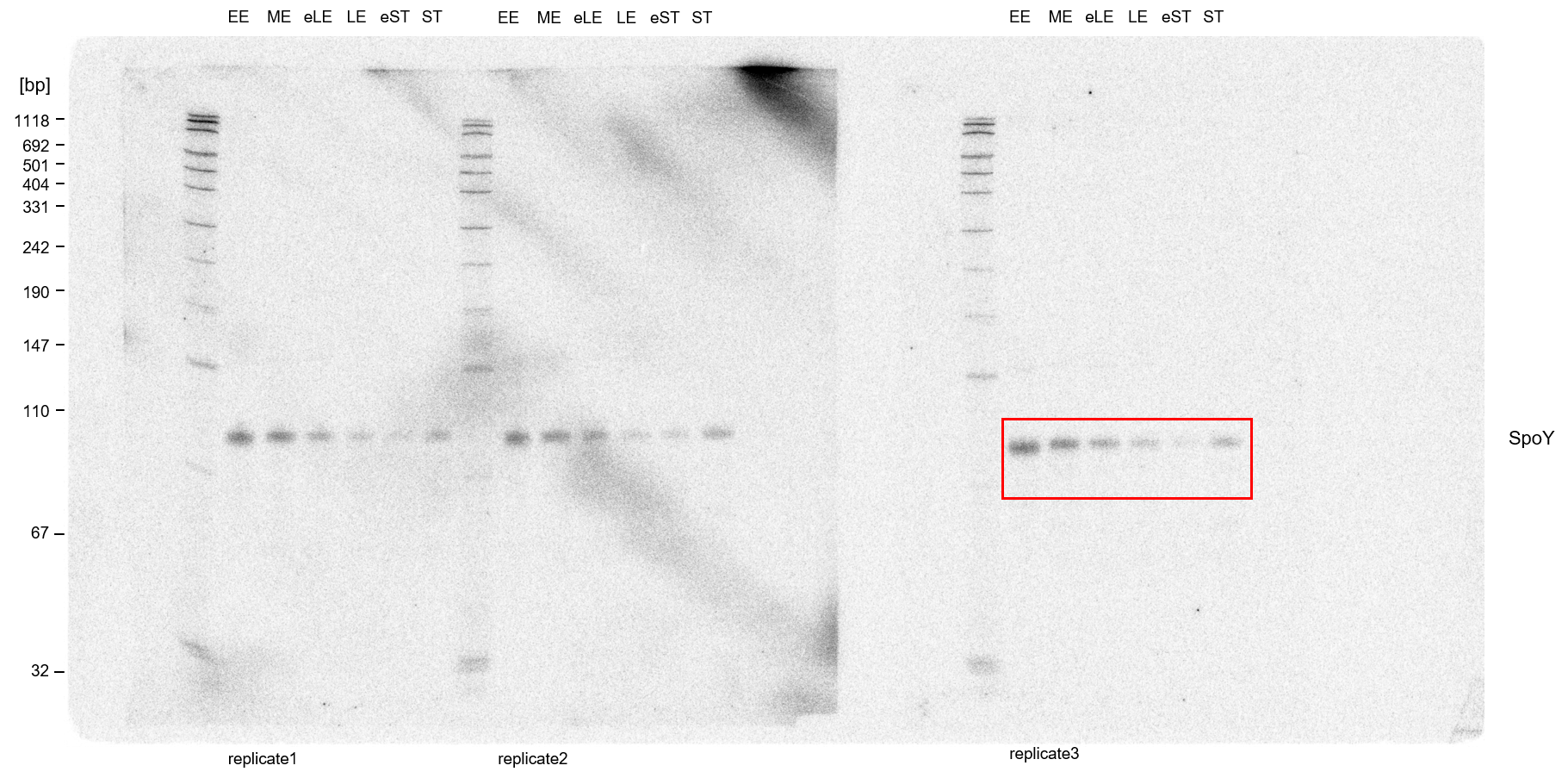

Supplement: Supplementary file 10 — Source Data for Figure 3 [file EMBJ-42-e112858-s015.zip › Figure 3/3C/3C-NB-SpoY_replicate1-3.tif]

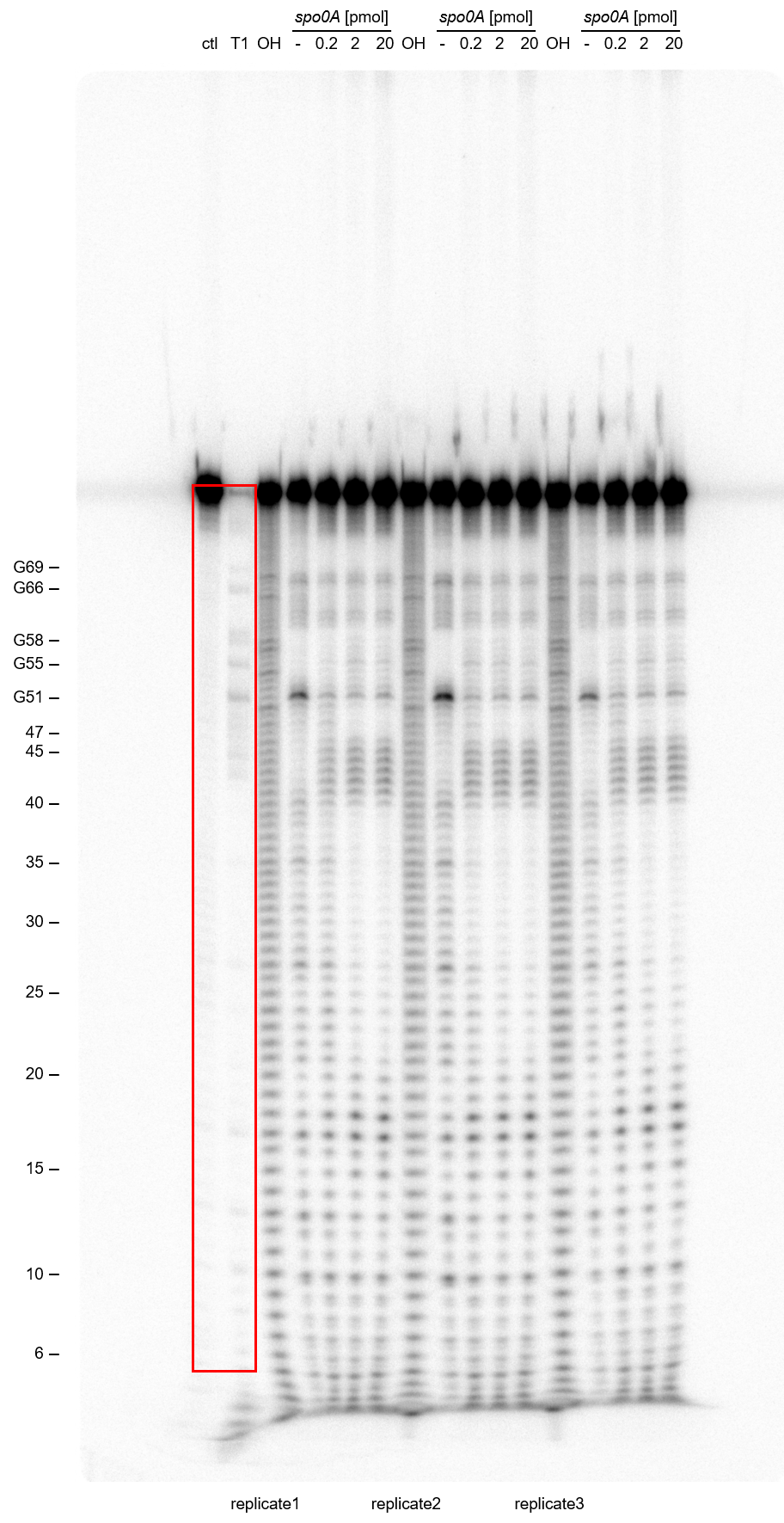

Supplement: Supplementary file 11 — Source Data for Figure 4 [file EMBJ-42-e112858-s002.zip › Figure 4/4B/4B-InLine-SpoX_replicate1-3.tif]

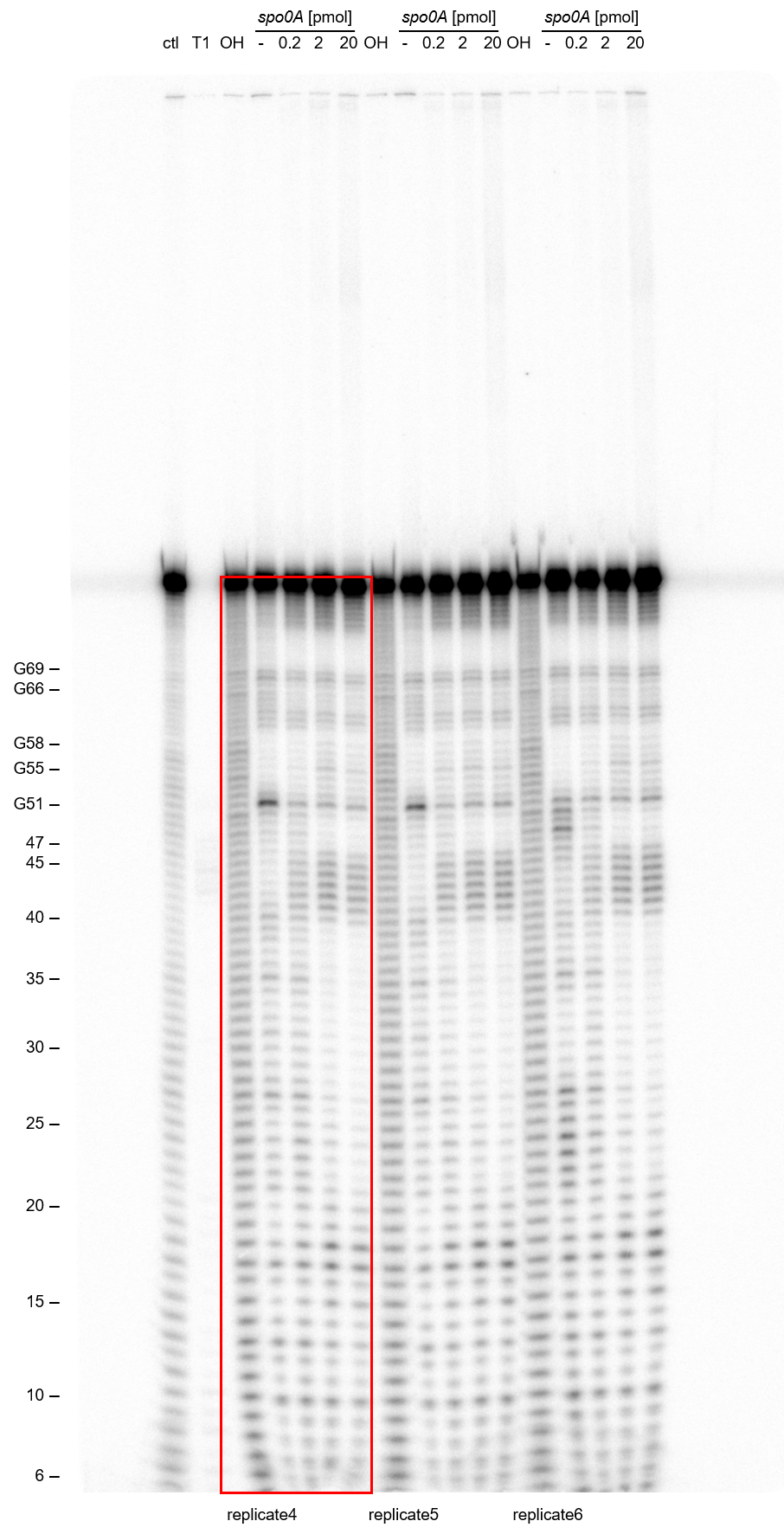

Supplement: Supplementary file 11 — Source Data for Figure 4 [file EMBJ-42-e112858-s002.zip › Figure 4/4B/4B-InLine-SpoX_replicate4-6.tif]

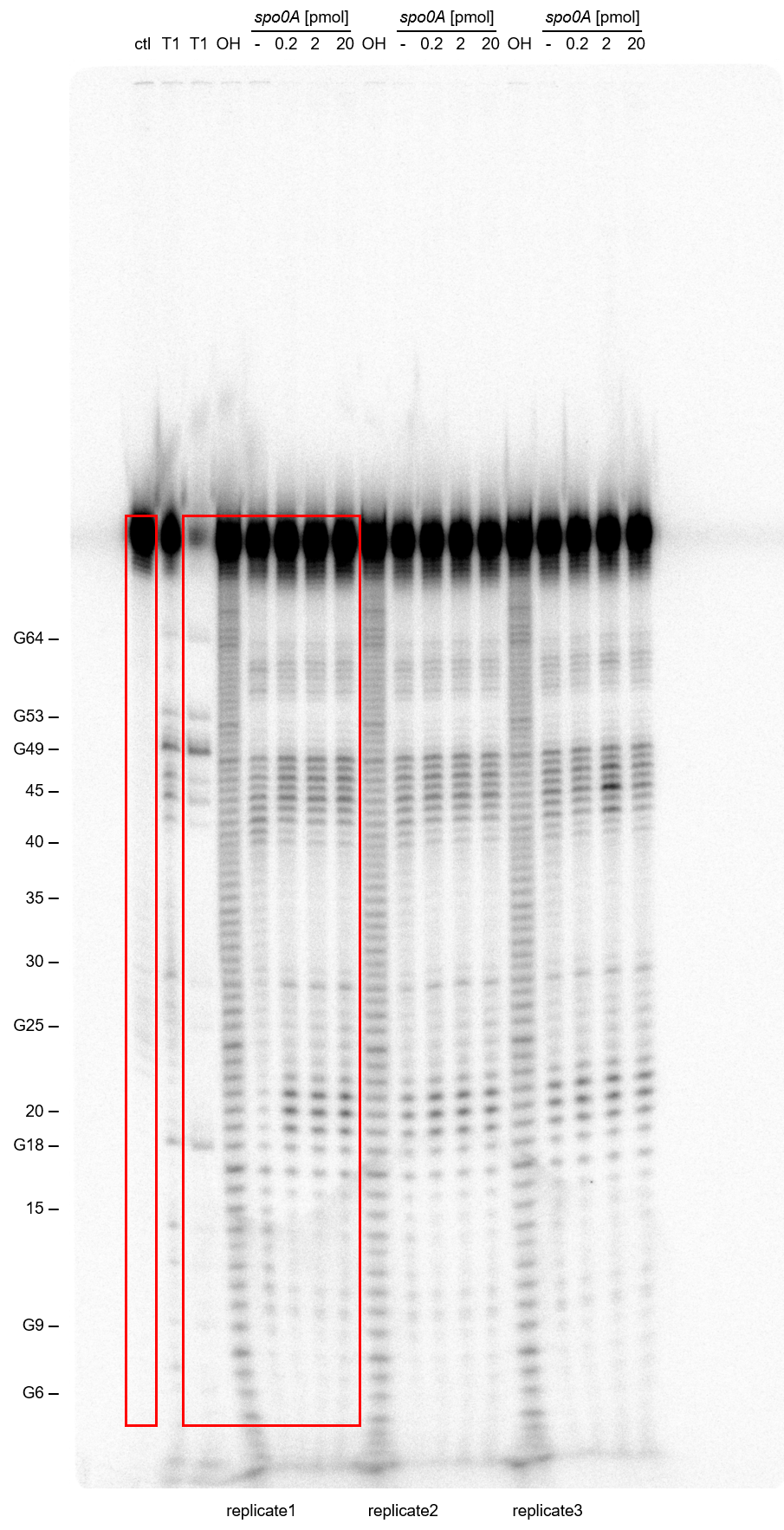

Supplement: Supplementary file 11 — Source Data for Figure 4 [file EMBJ-42-e112858-s002.zip › Figure 4/4B/4B-InLine-SpoY_replicate1-3.tif]

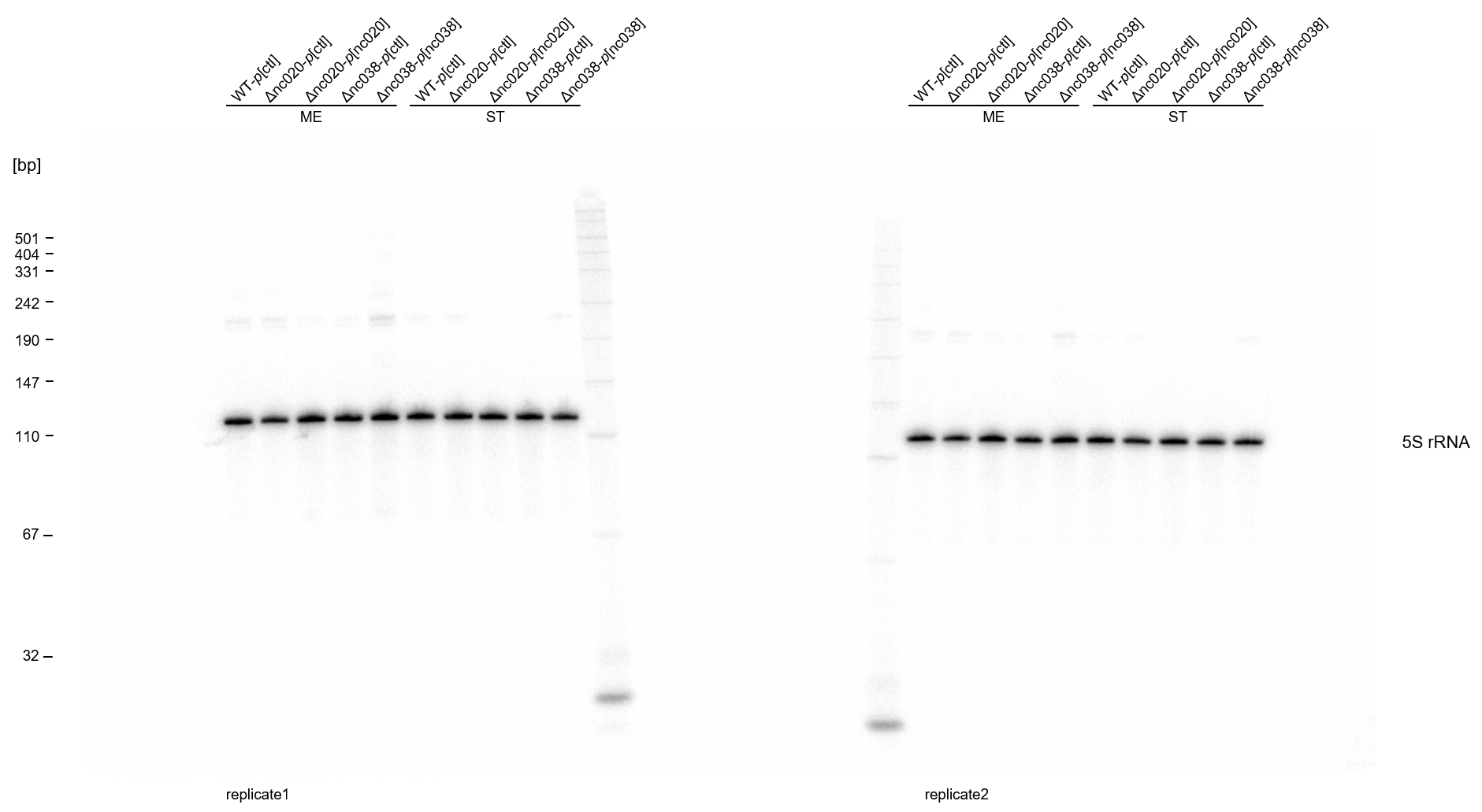

Supplement: Supplementary file 12 — Source Data for Figure 5 [file EMBJ-42-e112858-s007.zip › Figure 5/5/5-NB-5SrRNA-replicate1-2.tif]

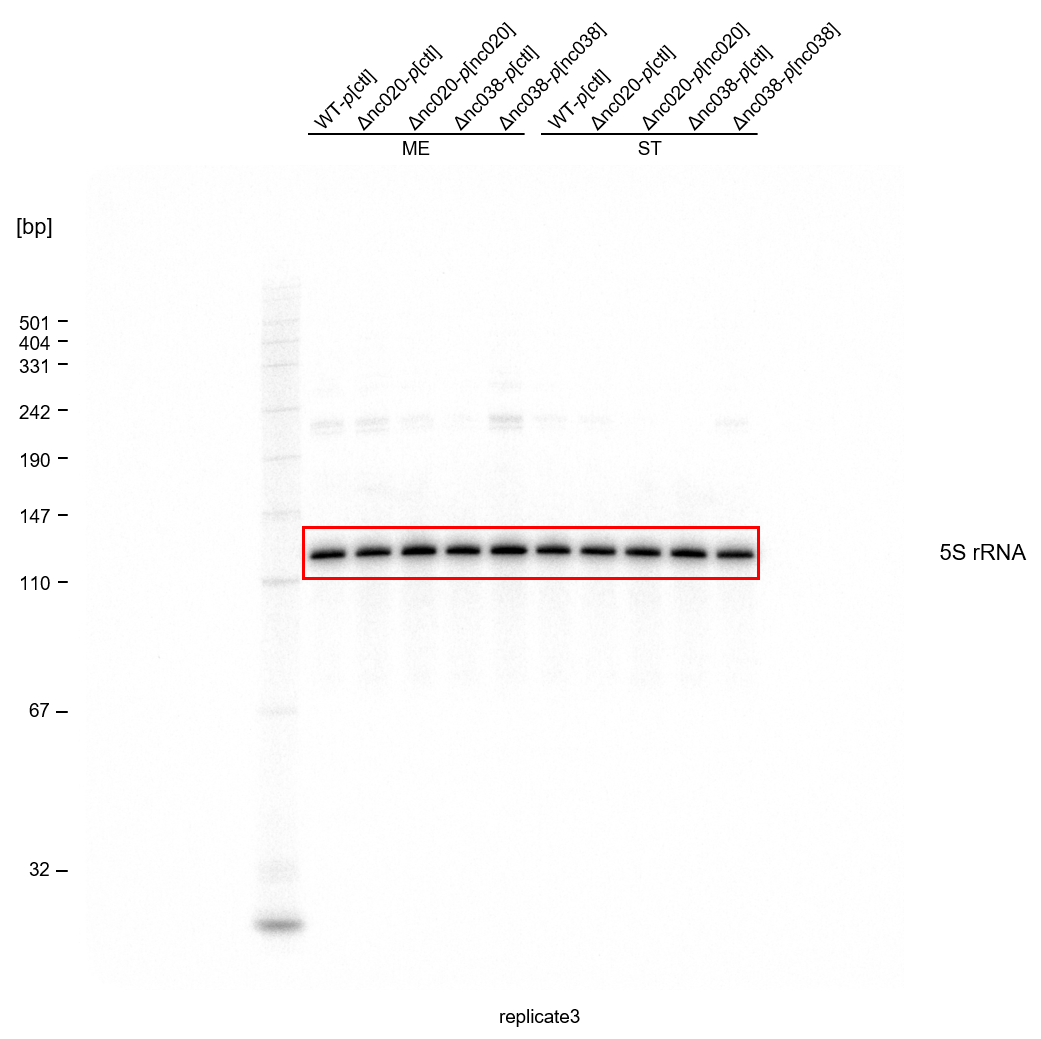

Supplement: Supplementary file 12 — Source Data for Figure 5 [file EMBJ-42-e112858-s007.zip › Figure 5/5/5-NB-5SrRNA-replicate3.tif]

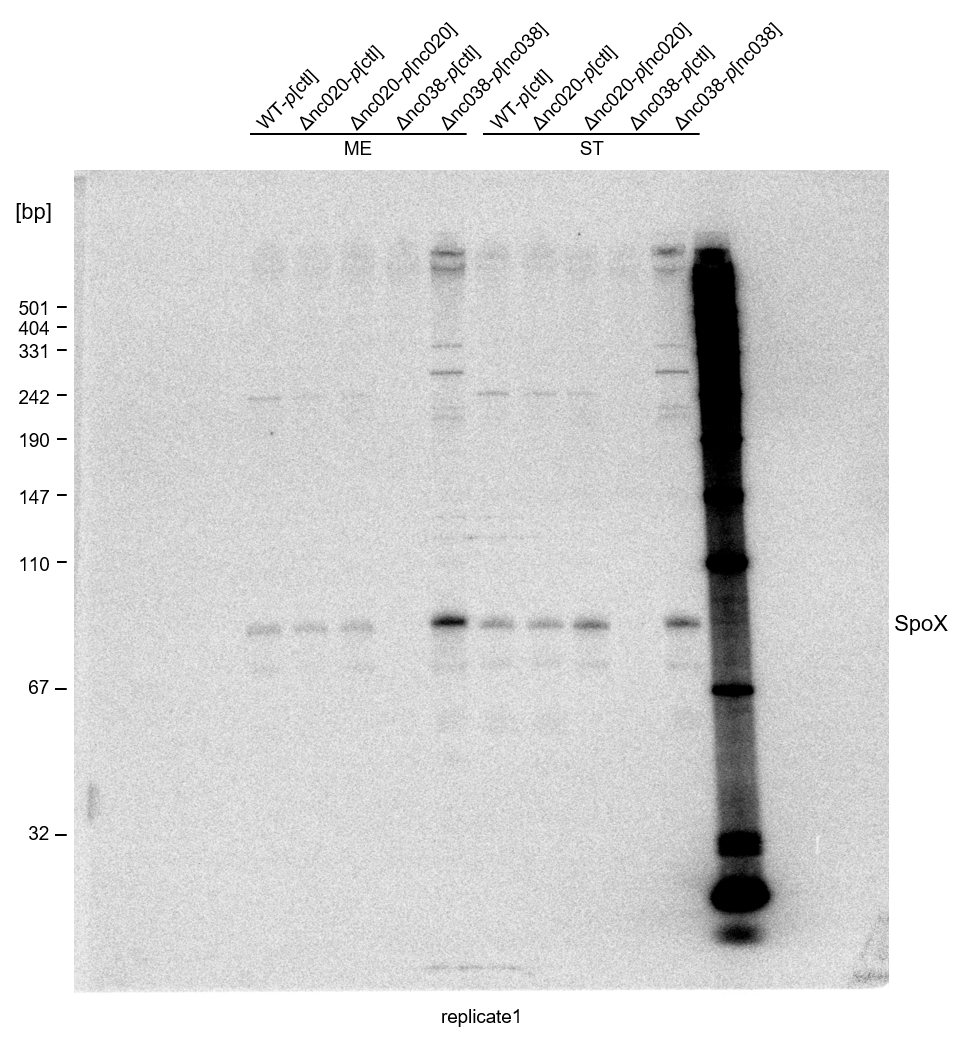

Supplement: Supplementary file 12 — Source Data for Figure 5 [file EMBJ-42-e112858-s007.zip › Figure 5/5/5-NB-SpoX_replicate1.tif]

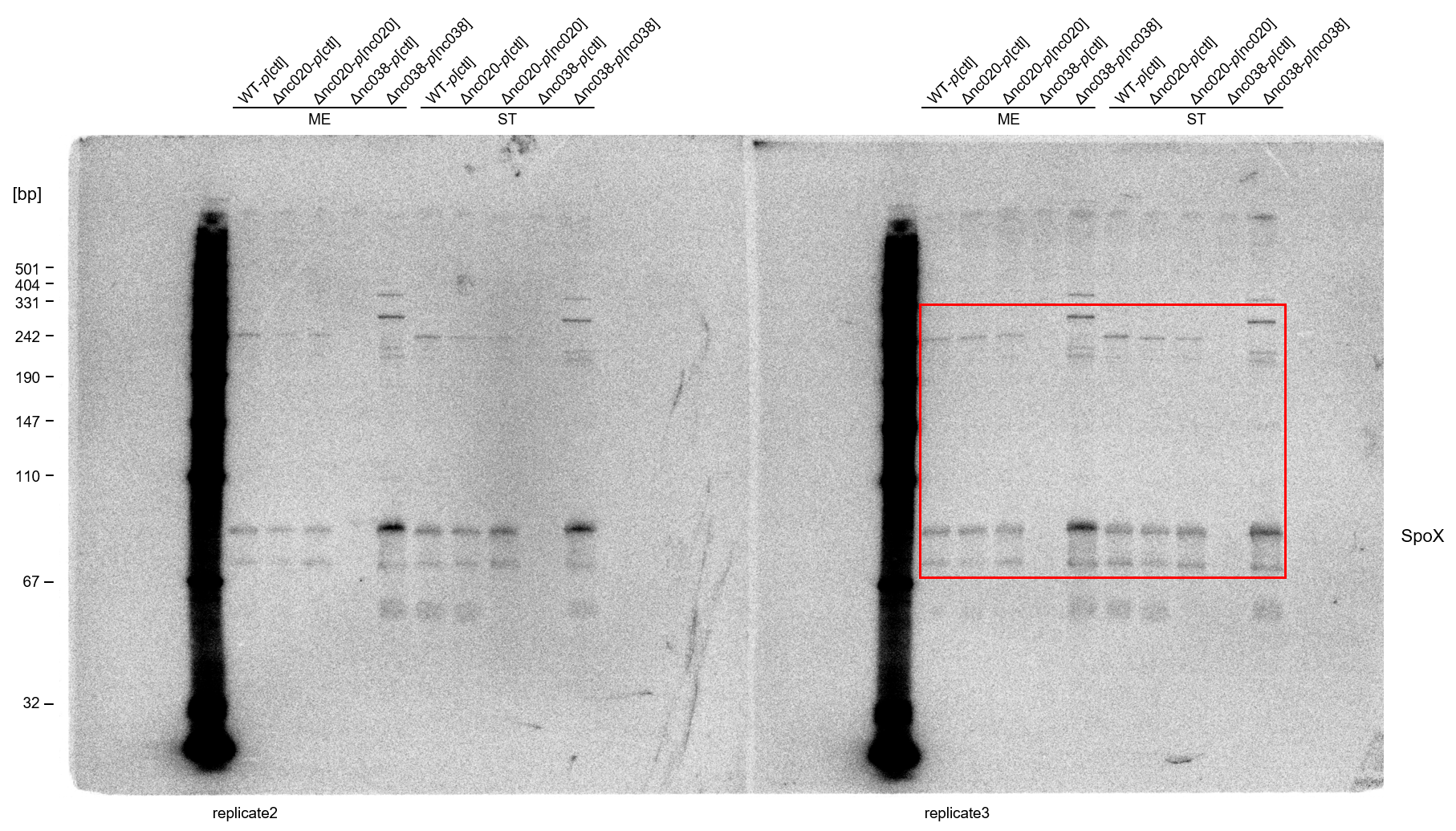

Supplement: Supplementary file 12 — Source Data for Figure 5 [file EMBJ-42-e112858-s007.zip › Figure 5/5/5-NB-SpoX_replicate2-3.tif]

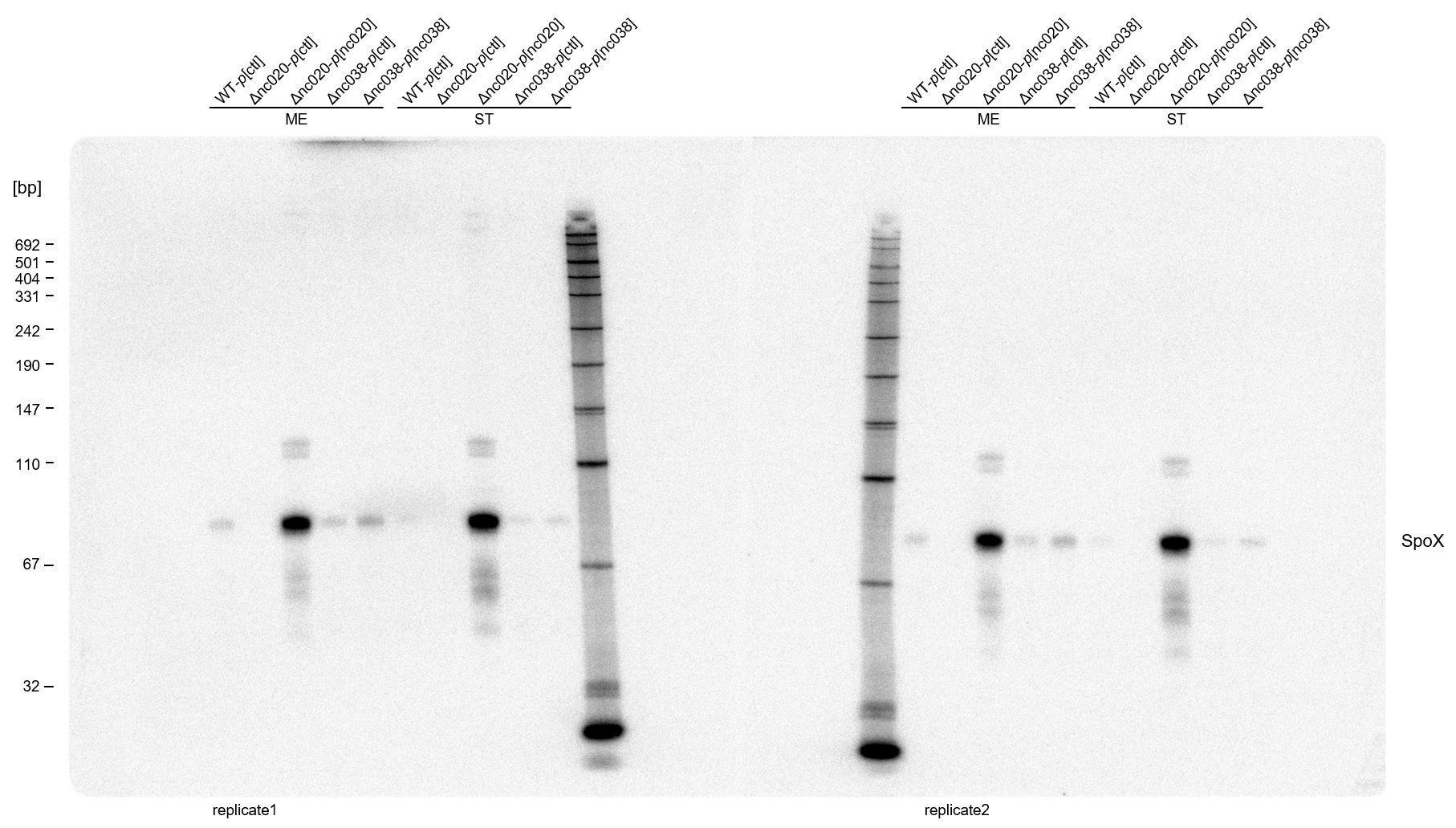

Supplement: Supplementary file 12 — Source Data for Figure 5 [file EMBJ-42-e112858-s007.zip › Figure 5/5/5-NB-SpoY_replicate1-2.tif]

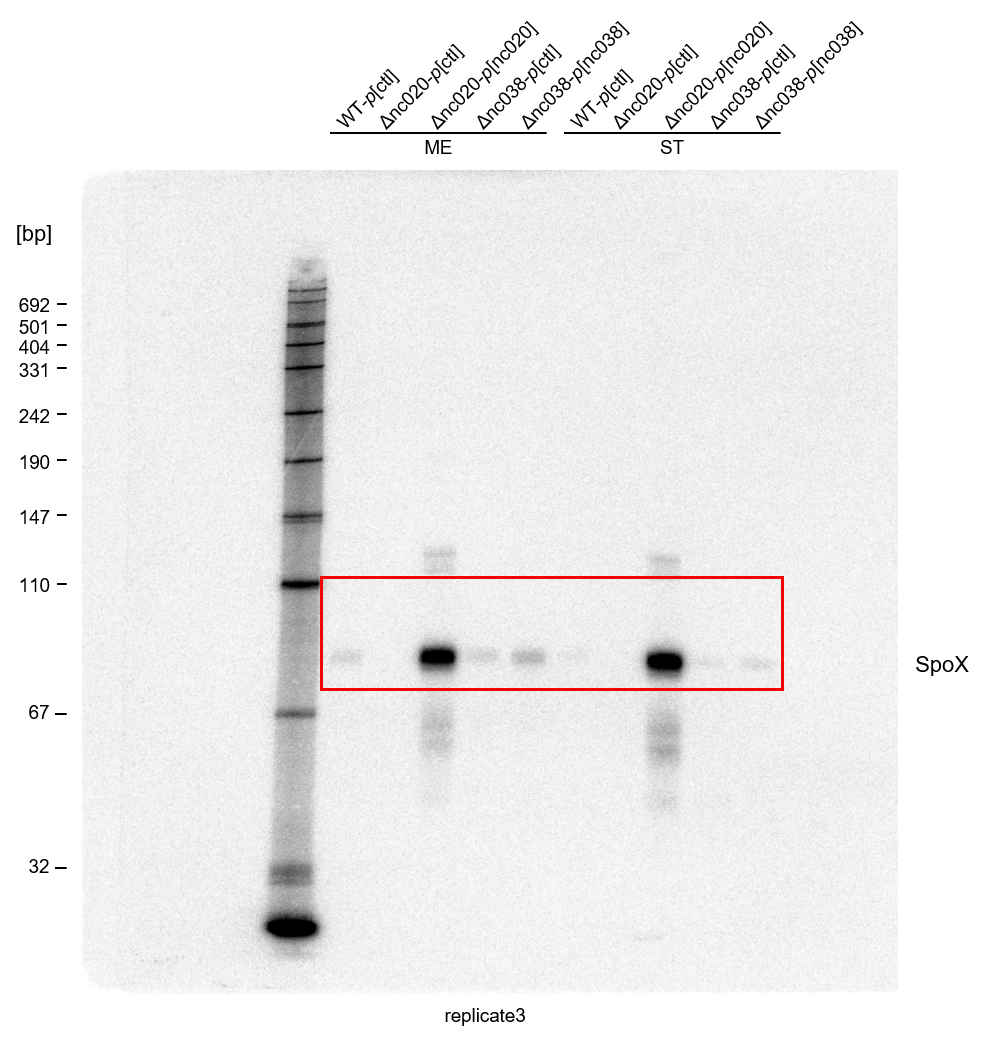

Supplement: Supplementary file 12 — Source Data for Figure 5 [file EMBJ-42-e112858-s007.zip › Figure 5/5/5-NB-SpoY_replicate3.tif]

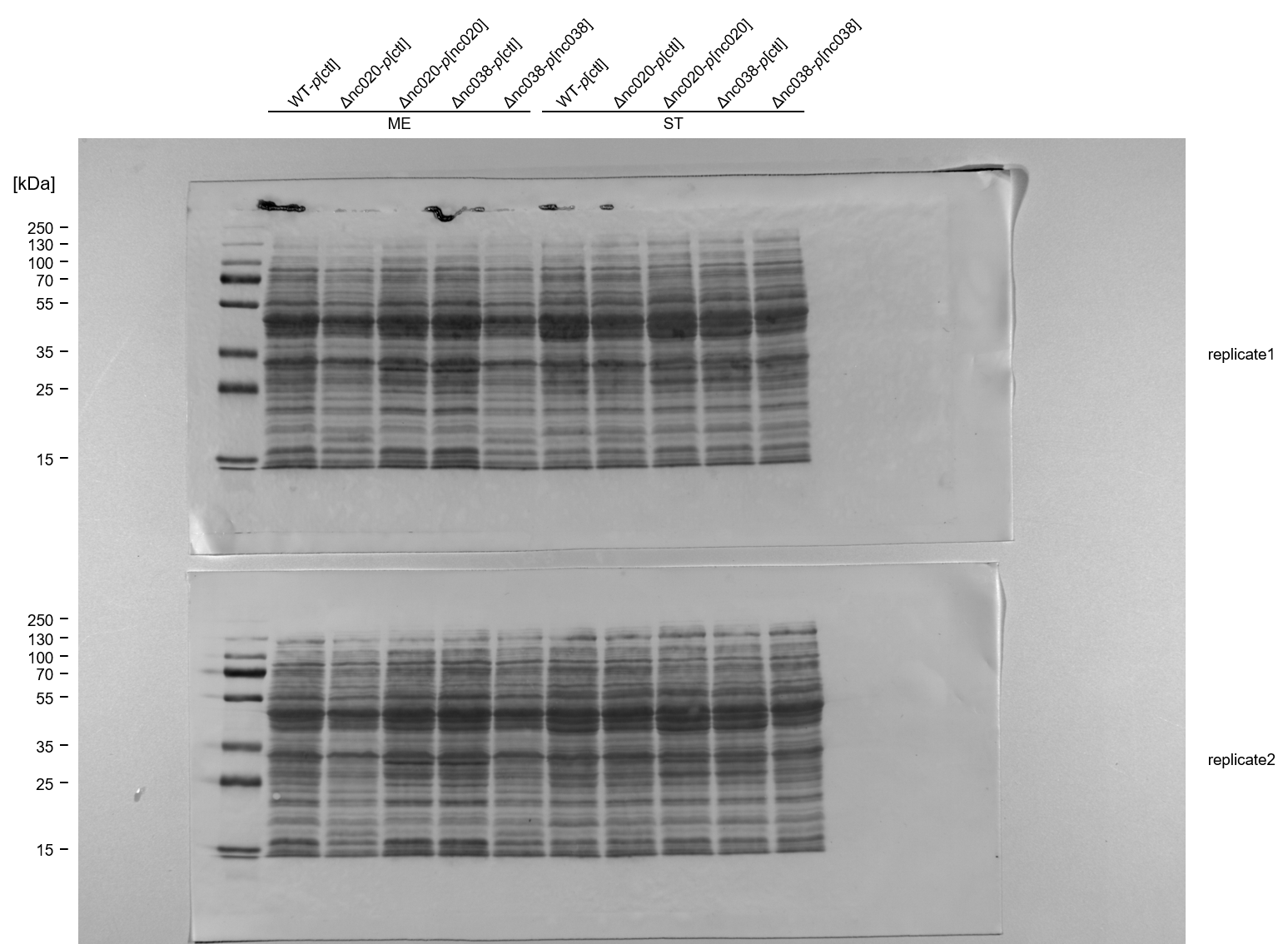

Supplement: Supplementary file 12 — Source Data for Figure 5 [file EMBJ-42-e112858-s007.zip › Figure 5/5/5-WB-PonceauS_replicate1-2.tif]

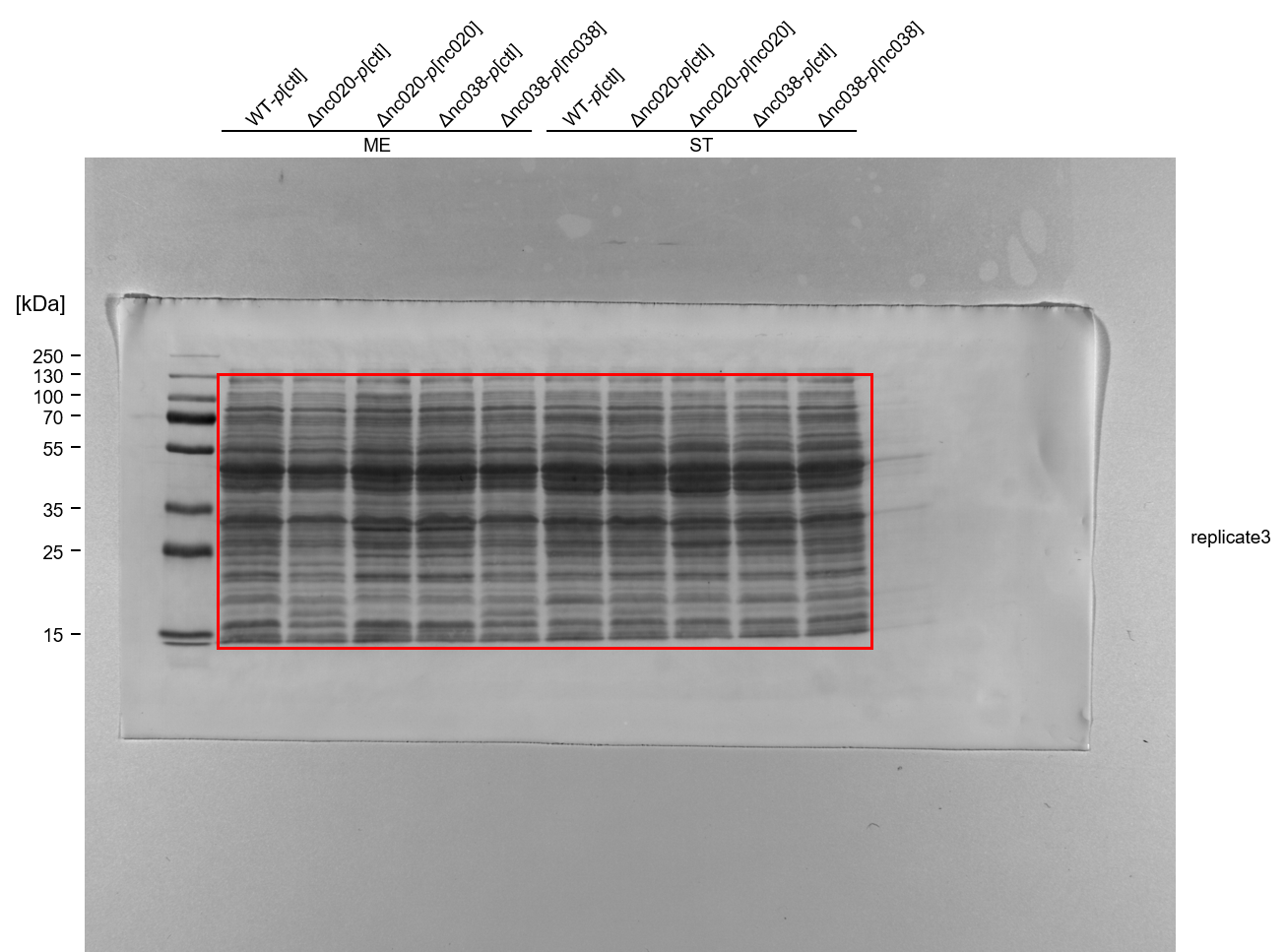

Supplement: Supplementary file 12 — Source Data for Figure 5 [file EMBJ-42-e112858-s007.zip › Figure 5/5/5-WB-PonceauS_replicate3.tif]

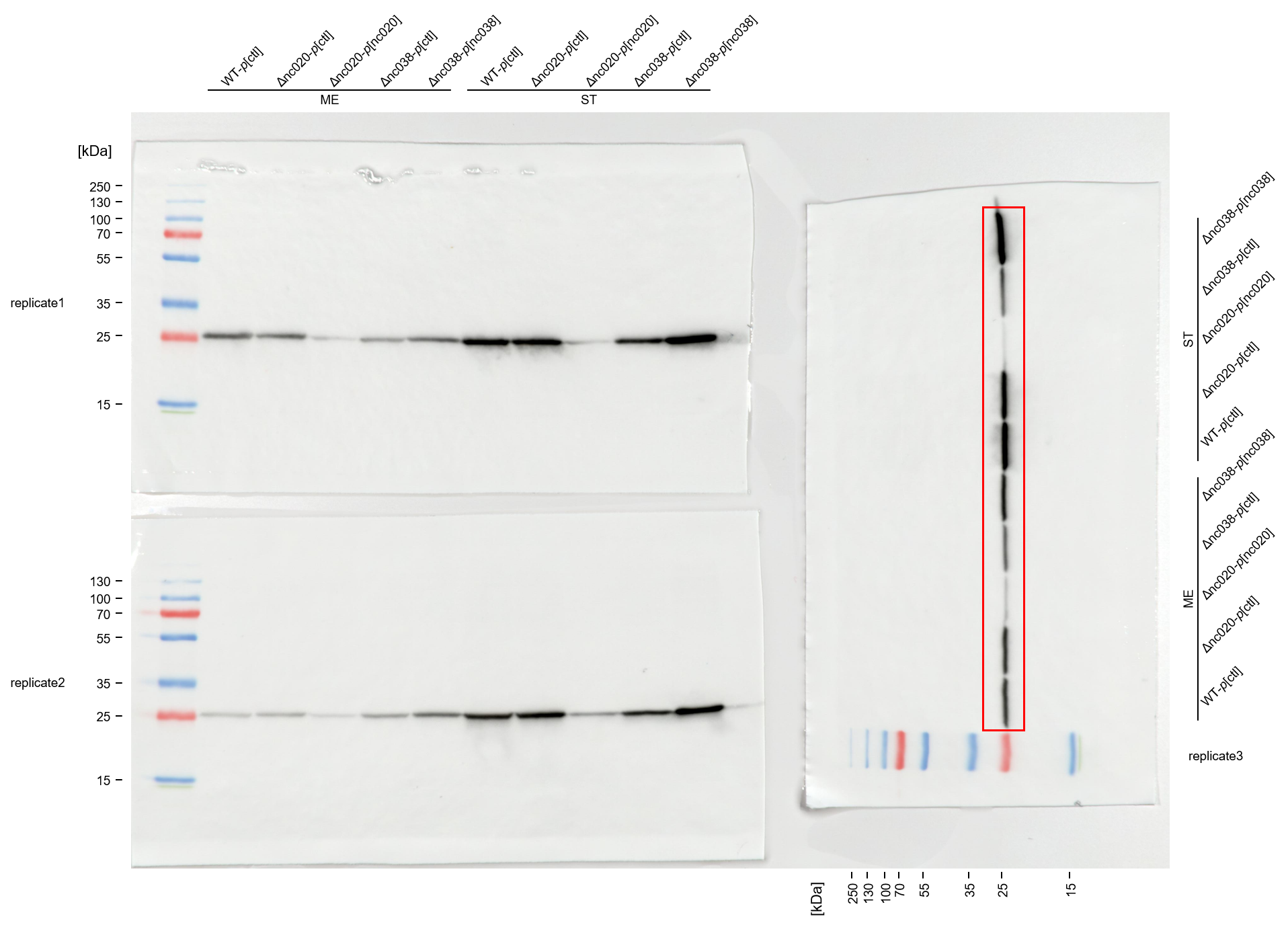

Supplement: Supplementary file 12 — Source Data for Figure 5 [file EMBJ-42-e112858-s007.zip › Figure 5/5/5-WB-Spo0A_replicate1-3.tif]
