# Supplementary material for: A network of small RNAs regulates sporulation initiation in Clostridioides difficile
Source: EMBO J. 2023 May 4;42(12):e112858. doi: 10.15252/embj.2022112858 (PMC10267692; doi:10.15252/embj.2022112858)
Supplement: Supplementary file 16 [file EMBJ-42-e112858-s008.pdf]

A network of small RNAs regulates sporulation initiation in *C. difficile*

Manuela Fuchs, Vanessa Lamm-Schmidt, Tina Lenče, Johannes Sulzer, Arne Bublitz, Janet Wackenreuter, Milan Gerovac, Till Strowig, and Franziska Faber
DOI: 10.15252/embj.2022112858

Corresponding author(s): Franziska Faber (franziska.faber@uni-wuerzburg.de)

Review Timeline:

Submission Date:	19th Oct 22
Editorial Decision:	21st Nov 22
Revision Received:	23rd Feb 23
Editorial Decision:	21st Mar 23
Revision Received:	23rd Mar 23
Accepted:	29th Mar 23

Editor: Ieva Gailite

Transaction Report:

(Note: With the exception of the correction of typographical or spelling errors that could be a source of ambiguity, letters and reports are not edited. Depending on transfer agreements, referee reports obtained elsewhere may or may not be included in this compilation. Referee reports are anonymous unless the Referee chooses to sign their reports.)

Dear Dr. Faber,

Thank you for submitting your manuscript for consideration by the EMBO Journal. We have now received comments from three reviewers, which are included below for your information.

As you will see from the reports, all reviewers find the study of interest, but also find that further mechanistic insights into the mode of SpoX and SpoY action would be needed before they can support publication here. Referee #3 also points out issues with the number of biological replicates and appropriate controls.

If you find that you are able to address the main issues raised by the reviewers, I would be happy to consider a revised version of the manuscript. I think it would be helpful to discuss the revision in more detail via email or phone/videoconferencing - please let me know which option you prefer. I should also add that it is The EMBO Journal policy to allow only a single major round of revision and that it is therefore important to resolve the main concerns at this stage.

We generally allow three months as standard revision time. As a matter of policy, competing manuscripts published during this period will not negatively impact on our assessment of the conceptual advance presented by your study. However, please contact me as soon as possible upon publication of any related work to discuss the appropriate course of action. Should you foresee a problem in meeting this three-month deadline, please let us know in advance to arrange an extension.

When preparing your letter of response to the referees' comments, please bear in mind that this will form part of the Review Process File and will therefore be available online to the community. For more details on our Transparent Editorial Process, please visit our website: https://www.embopress.org/page/journal/14602075/authorguide#transparentprocess. Please also see the attached instructions for further guidelines on preparation of the revised manuscript.

Please feel free to contact me if you have any further questions regarding the revision. Thank you for the opportunity to consider your work for publication. I look forward to discussing your revision.

With best regards,

Ieva

Ieva Gailite, PhD
Senior Scientific Editor
The EMBO Journal
Meyerhofstrasse 1
D-69117 Heidelberg
Tel: +4962218891309
i.gailite@embojournal.org

Instructions for preparing your revised manuscript:

Please make sure you upload a letter of response to the referees' comments together with the revised manuscript.

Please also check that the title and abstract of the manuscript are brief, yet explicit, even to non-specialists.

When assembling figures, please refer to our figure preparation guideline in order to ensure proper formatting and readability in print as well as on screen:
https://bit.ly/EMBOPressFigurePreparationGuideline
See also guidelines for figure legends: https://www.embopress.org/page/journal/14602075/authorguide#figureformat

At EMBO Press we ask authors to provide source data for the main manuscript figures. Our source data coordinator will contact you to discuss which figure panels we would need source data for and will also provide you with helpful tips on how to upload and organize the files.

IMPORTANT: When you send the revision we will require
- a point-by-point response to the referees' comments, with a detailed description of the changes made (as a word file).
- a word file of the manuscript text.
- individual production quality figure files (one file per figure)
- a complete author checklist, which you can download from our author guidelines

(https://www.embopress.org/page/journal/14602075/authorguide).
- Expanded View files (replacing Supplementary Information)
Please see out instructions to authors
https://www.embopress.org/page/journal/14602075/authorguide#expandedview

Please remember: Digital image enhancement is acceptable practice, as long as it accurately represents the original data and conforms to community standards. If a figure has been subjected to significant electronic manipulation, this must be noted in the figure legend or in the 'Materials and Methods' section. The editors reserve the right to request original versions of figures and the original images that were used to assemble the figure.

Further information is available in our Guide For Authors: https://www.embopress.org/page/journal/14602075/authorguide

We realize that it is difficult to revise to a specific deadline. In the interest of protecting the conceptual advance provided by the work, we recommend a revision within 3 months (19th Feb 2023). Please discuss the revision progress ahead of this time with the editor if you require more time to complete the revisions.

Referee #1:

Fuchs et al. report the results from carrying out RIL-seq to examine the Hfq-dependent RNA-RNA interactome for the enteric pathogen Clostridioides difficile. The authors identify several new sRNAs but focus on two SpoX and SpoY which have opposing effects on the expression of Spo0A the master regulator of sporulation. By analyzing ΔspoX and ΔspoY deletion mutants, Fuchs et al. find that these two sRNAs have global effects on gut colonization and intestinal sporulation in antibiotic-treated mice.

This work provides a valuable database that is easily accessible through an interactive browser and identifies two intriguing regulators of C. difficile sporulation. However, I have some suggestions for improving the study.

1. Figure 2B (this figure should have more panels labeled): The authors need to show more of the northern blots presented here. Many sRNAs are subject to processing and the readers need to be able to evaluate this. The expression of nc153 is not very convincing.

2. Figure 3C: The northern detection of SpoX and SpoY could also be improved; the 237 nt form of SpoX is barely detectable and more of the SpoY blot should be shown. The study would also be more complete with further analysis of the factors/conditions that control SpoX and SpoY expression.

3. Figure 3D: It should be relatively straightforward to carry out mutational analysis to test the predicted model of SpoX activation through changes in the secondary structure of the spo0A 5' UTR.

4. The discussion is very speculative. Given the available tools such as antibodies to Spo0A, the authors could text whether spo0A really is a central target of sRNA-mediated regulation or if SpoX and SpoY act as sponges of each other.

5. The authors should present a model showing how they propose SpoX and SpoY intergrate into the regulatory network controlling sporulation.

More minor comments:

6. Figure 6: The authors need to present these data in a more user-friendly manner. Currently, the eye is drawn to the statistical analysis rather than the data.

7. Editorial suggestions:
--Page 4, Lines 100-101: The sentence "Interestingly, sRNAs generally show a clear preferences for either position 1 or 2." is unclear and seems to be in contradiction to the preceding and following sentences.
--Page 12, Line 349: The authors probably mean flhDC rather than flhDS.

Referee #2:

Fuchs et al in the manuscript entitled "A network of small RNAs regulates sporulation initiation in C. difficile"describes the use of the global approach RIL-seq to study sRNA regulation in Clostridium difficile at an early stage of sporulation. Using Hfq as a bait, the method allows to identify the RNA-RNA interactome among those, sRNA-mRNA couples, whose pairing is dependent on the chaperone. The authors discovered two novel sRNA, SpoX and SpoY acting in an inverse manner on the same transcript, encoding the master regulator of sporulation Spo0A. Diverse in vitro and in vivo experiments concluded that SpoX activates spo0A translation and sporulation, probably by liberating the RBS, while in contrast SpoY binds close to the AUG and inhibits spo0A translation, and sporulation. Nevertheless, the mechanisms of regulation are not clear and should be investigated regarding the two possible TSS of the spo0A transcript. Finally, intraperitoneal mice model of infection revealed a global effect of both sRNA on gut colonization and intestinal infection. These results may also need further investigations. The method has been published in 2016 and was proven to be highly effective in E. coli and Salmonella to decipher sRNA regulatory network in bacteria. Because the proof of concept is well established for few years, my feeling is that the authors did not exploit and validate extensively their results. While the experiments are generally well conducted and described, I have comments and suggestions to hopefully improve the impact of the article.

Major comments

L95: in the RIL-seq data analysis, how were TSS considered, were they experimentally defined and particularly for sRNA studied in Figure 2B and SpoX, SpoY or both pTSS ans sTSS of spo0A mRNA (Figure 3BD)?

L132: what was the rational in the choice of sRNAs? Are nc152 and 153 3'UTR-derived sRNA generated by maturation or are they bona fide ncRNAs? Why is nc155 not considered as an IGR ?

L181: what about the mRNA expression of spo0A? did the two forms follow the same pattern of expression? Are transcripts affected by SpoX and SpoY regulation? Northern experiments in the wild-type and the two deletion mutants ΔspoX and ΔspoY strains may bring clues about the mechanisms of regulation.

L203: surprisingly base pairing predictions were presented with the short transcript of spo0A, while EMSA were performed with the long form of spo0A. Which form was used in the In-line probing assay? The model of SpoY and SpoX binding to sTSS or pTSS is difficult to interpret and should be discussed.

L217: The complex formation of SpoY with spo0A (pTSS) even in presence of Hfq is not convincing, free radiolabeled SpoY did not decrease. What about the affinity of SpoY with sTSS mRNA of spo0A?

L232 (supp Figure 5D): which 5'UTR length of spo0A was used? The results with the spo0A fusion in combination with one SpoX or SpoY were not shown.

L242 (Figure 4C, right panel): the level of mCherry induced in presence of SpoX was not significantly different from the level in the ΔspoX background.

L257 (Figure 5A): This panel should be combined to Figure 4, as panel D. Then Figure 6 may be exchanged with Figure 5 to respect the order in the text.

L266: SpoX and SpoY relative levels are missing and must be added to confirm that they are expressed in the sporulation conditions used in the experiments.

L295-298 (Figure 7D): the numbers of vegetative bacteria or spores were mostly not significant for the mice infected with the ΔspoX mutant compared to the mice infected with the wild-type, except at day 7. It is surprising, because the ΔspoX mutant had the most pronounced effect in spo0A regulation or sporulation. In contrast, the deletion of spoY had no effect on Spo0A synthesis and sporulation but was less infectious. Other targets of SpoX and SpoY identified in RIL-seq may be involved in the infection process as proposed for cwp2 or cwpV. The manuscript might have a better impact if at least these two mRNAs are shown to be direct targets of SpoX and SpoY.

L304: the discussion section is rather short and speculative, and some questions could be adressed: what could be the transcriptional regulators of SpoX and Y? what is the purpose to inhibit and activate the same transcript at the same time (stage 0 in Figure 6)? which sRNA would come first ? In the respective deletion mutants of SpoY or SpoX, we should see the effect of SpoX respectively SpoY, but they behave almost as WT, what are the suggestions?

L660: the medium used in the northern blots must be provided. Expression varies depending on the nutrients.

L738-39 L748-750: were the conditions used in the reporter system assays (TY at 6 hours) the same condition as the RIL-seq (TY medium at OD 1,2)? please, indicate OD instead of timing.

L748-750: the culture conditions to verify sRNA expression (BHI, OD=1) must follow the conditions of the reporter assays. BHI contains glucose, in contrast to TY, expression of RNAs may vary. Moreover, the strain containing the mutated derivatives of

SpoY and SpoX are missing (Fig S5E).

L758-59: were the conditions used in the western blot comparable to the condition of the RIL-seq? please, indicate OD instead of timing. Northern blot showing the SpoX and SpoY should be added as controls.

Minor comments

L122: 3'UTRs are also reservoirs of sRNA, any example known in C. difficile that could have been hit ?

L215: 5A instead of 4A.

L708/718/723: how can the authors measure the concentration of labelled sRNA, while the labelling is not 100% efficient, better to indicate the radioactive counts.

L902 and references' list: all the bacterial names should be italized.

Referee #3:

Fuches et al. presents a well-written, intriguing story that will appeal to a broad audience. Briefly, they performed C. difficile RIL-seq to identify RNA interacting partners of Hfq-associated sRNAs in rich medium. They discovered numerous novel RNA-RNA interactions and identified several novel sRNAs. They also created a searchable database for their data sets which will be of interest within the Clostridial community. Further, they detailed the mechanistic role of two sRNAs, renamed SpoX and SpoY, that directly interact with the spo0A mRNA to post-transcriptionally regulate its expression and influence sporulation. Finally, they provided some evidence that SpoX and SpoY may impact colonization and spore burden in a mouse model. This study lacks some controls and needs to ensure the correct number of independent biological replicates are performed (see comments). Finally, mRNA stability studies to support or exclude whether the sRNAs affect spo0A transcript stability or translation would strengthen the mechanistic studies, and in vitro toxin analyses would provide more context for the animal studies.

Fig. 4C /Lines 248-249 - The translational mCherry fusions confirm that SpoX and SpoY's affects do occur post-transcriptionally and may suggest that SpoX and SpoY affect spo0A translation, but these experiments do not exclude the possibility that SpoX and (less likely) SpoY impact spo0A mRNA stability rather than translation. spo0A mRNA stability experiments in the parent strain and the respective spoX and spoY mutants would rule out this possibility.

Fig. 5A - An additional replicate needs to be performed for an n = 3 as is standard practice within the field and is required for reproducibility and rigor.

Fig. 5BC - Sporulation assays and phase-contrast microscopy: Generally, biological triplicates performed simultaneously are not standard in the field for rigor. Three independent experiments (one biological replicate per experiment is adequate) need to be performed to ensure reproducibility and rigor.

Lines 367-368 - Spo0A impacts on toxin production seem to be variable in the 012 ribotype, depending on the conditions and the specific strains tested (e.g. Underwood et al used 630Δerm, not 630). Because animal studies suggest that toxin production is not affected by the spoX or spoY mutation, one optional consideration is to perform quick TcdA/TcdB ELISAs with the parent strain and the spoX/spoY mutants in vitro to see if toxin production in these mutants correlates with the indirect results seen in the animal studies.

Lines 387-388 - The cwp2 promoter is used to control spo0A 5' UTR transcription in the mCherry constructs even though cwp2 is a potential SpoY target. Are any of the cwp2 regions predicted to interact with SpoY included in these promoter constructs?

Fig. 7D - Enumeration of spores from mice feces - were heat-treated fecal samples also plated on bioMérieux C. difficile agar without taurocholate supplementation to ensure all vegetative cells were eliminated? It can be difficult to kill all vegetative cells in animal feces with heat treatment, and this is an important control. Especially given the sporulation frequency results in vitro with the spoX mutant, fewer spores would have been anticipated in the mouse model.

Minor comments:

Figure 2A - What are the red shaded areas in the operons of the sRNAs nc152, nc153, and nc 155?

Figure 2C - It would be helpful to include the identity of the sRNA that interacts with each intergenic region (e.g. CDIF630nc_161 interacting with sigG) in the figure panel.

Line 204 - Define "seed region" for a broader audience that may be unfamiliar with sRNA terminology.

Lines 262-281 - Discuss the data in the order they appear in the results, so please move the sporulation frequency data up in the results sections and move the qRT-PCR results below that section.

Line 345-347 - Regarding the minor phenotype in the spoY mutant - it is possible that the negative effects of SpoY are masked in the presence of SpoX (especially given the regions spanned in the chimera reads); however, the impact of SpoY and SpoX may also be due to growth phase dependent expression based on northern blotting. These possibilities should also be addressed in the discussion.

Line 765 - Instead of 340 mM, do you mean 340 mA?

Re: EMBOJ-2022-112858
First revision: A network of small RNAs regulates sporulation initiation in *C. difficile*

Editorial suggestions and summary of changes to the manuscript

As you will see from the reports, all reviewers find the study of interest, but also find that further mechanistic insights into the mode of SpoX and SpoY action would be needed before they can support publication here. Referee #3 also points out issues with the number of biological replicates and appropriate controls.

Response: First of all, we would like to thank the editor for providing clear guidance throughout the review process and the reviewers for all their helpful comments. The following bullet list summarizes the changes we made to the manuscript. This is followed below by a point-by-point response to the referee's comments.

- We have performed additional in line probing experiments to gain more insight into how SpoX binding to the *spo0A* 5'UTR can activate Spo0A translation through changes in RNA secondary structure
- We either performed or added (where already available) all requested control experiments, including EMSAs for interaction of both sRNAs with the short *spo0A* 5'UTR (Supplementary Figure 6D)
- We have validated additional targets for SpoX/SpoY that were predicted through the RIL-seq analysis
- We have clarified throughout the manuscript which *spo0A* 5'UTR version was used in each experiment
- We have added a model showing how SpoX and SpoY integrate into the larger regulatory network that controls sporulation initiation in *C. difficile* (Figure 9)

Point-by-point response to the referee's comments

Referee #1:

Fuchs et al. report the results from carrying out RIL-seq to examine the Hfq-dependent RNA-RNA interactome for the enteric pathogen Clostridioides difficile. The authors identify several new sRNAs but focus on two SpoX and SpoY which have opposing effects on the expression of Spo0A the master regulator of sporulation. By analysing ΔspoX and ΔspoY deletion mutants, Fuchs et al. find that these two sRNAs have global effects on gut colonization and intestinal sporulation in antibiotic-treated mice.
This work provides a valuable database that is easily accessible through an interactive browser and identifies two intriguing regulators of C. difficile sporulation. However, I have some suggestions for improving the study.

Major comments

Q1: Figure 2B (this figure should have more panels labelled): The authors need to show more of the northern blots presented here. Many sRNAs are subject to processing and the readers need to be able to evaluate this. The expression of nc153 is not very convincing.

Response: We have now arranged Figure 2 into four instead of three panels. Regarding nc153, we agree that the signal is weak but since we can detect it in all 3 replicates (see source data), we are confident, that this is sufficient evidence for its expression. Due to space limitations, we have only provided full blots wherever we observed several isoforms for a sRNA e.g., in the case of SpoX. However, full-length northern blots for all shown data have been provided as source data.

Q2A: Figure 3C: The northern detection of SpoX and SpoY could also be improved; the 237 nt form of SpoX is barely detectable and more of the SpoY blot should be shown.

Response: We agree that the signal for the long SpoX isoform is much weaker than the signal for the small SpoX isoform. SpoX is difficult to detect probably due to its very high AT content (76%), which made the design of a northern probe with optimal annealing features challenging, and likely explains the overall weak NB signals. Nevertheless, the observed expression profile supports our hypothesis that the short isoform is the prominent isoform in this condition as discussed in the results (L190-195). Full-length northern blots for all sRNAs will be provided as source data.

Q2B: The study would also be more complete with further analysis of the factors/conditions that control SpoX and SpoY expression.

Response: We agree that this is a very interesting point, as discussed in the text (L379-390). To address the question, we tested expression of SpoY and SpoX across the growth curve in different media known to affect sporulation, including Brain Heart Infusion (BHI), TY + glucose, TY + fructose and 70:30 sporulation medium (Supplementary Figure 3). We see no clear repression or induction of expression in any of the media conditions for SpoY, while SpoX expression (short isoform) tends to be upregulated in TY-based medium and stationary phase of growth. However, a comprehensive analysis will be required to better understand which environmental signals and/or transcription factors (TF) control the expression of these sRNAs, for example by utilizing defined TF mutants or over-expression strains. These analyses will be part of future studies involving also the additional putative sRNA regulators of *spo0A* that were identified through our RIL-seq analysis.

Q3: Figure 3D: It should be relatively straightforward to carry out mutational analysis to test the predicted model of SpoX activation through changes in the secondary structure of the spo0A 5' UTR.

Response: We agree that it is interesting to see, if SpoX binding indeed leads to conformational changes of the spo0A 5'UTR. We addressed this question *via* in line probing with labelled *spo0A* using the long 5'UTR (Supplementary Figure 5C). As predicted, binding of SpoX to the *spo0A* 5'UTR resulted in increased degradation of the *spo0A* 5'UTR in proximity to its RBS upon binding of SpoX, which indicates an opening of the *spo0A* secondary structure in proximity to the RBS upon base-pairing with SpoX.

Q4: The discussion is very speculative. Given the available tools such as antibodies to Spo0A, the authors could test whether spo0A really is a central target of sRNA-mediated regulation or if SpoX and SpoY act as sponges of each other.

Response: Here, we would like to disagree with the reviewer. We think, we have provided sufficient evidence regarding the direct regulation of *spo0A* mRNA by SpoX and SpoY using several independent methodologies including EMSAs, in line probing and *in vivo* reporter assays (including seed mutants), in addition to showing an effect of both sRNAs on native Spo0A protein levels (Figure 5A). The potential sponging activity between both sRNAs is still possible,

of course. However, in our opinion, this would not exclude the direct base-pairing of either SpoY or SpoX with *spo0A* mRNA.

Q5: The authors should present a model showing how they propose SpoX and SpoY integrate into the regulatory network controlling sporulation.

Response: We summarized our findings in a comprehensive model (Figure 9).

Minor comments

Q6: Figure 6: The authors need to present these data in a more user-friendly manner. Currently, the eye is drawn to the statistical analysis rather than the data.

Response: We have adjusted the graph accordingly and visually minimized the appearance of the statistical analysis.

Editorial suggestions

Q7A: Page 4, Lines 100-101: The sentence "Interestingly, sRNAs generally show a clear preference for either position 1 or 2." is unclear and seems to be in contradiction to the preceding and following sentences.

Response: Thank you for pointing this out. We have clarified our statement by changing the sentence to "Although most sRNAs were predominantly found at position 2, such as nc159, a few sRNAs showed a clear preference for position 1, including nc083". In combination with the previous and the following sentence, this hopefully clarifies the matter.

Q7B: Page 12, Line 349: The authors probably mean flhDC rather than flhDS.

Response: Yes. We have changed *flhDS* to *flhDC* in the text.

Referee #2:

Fuchs et al in the manuscript entitled "A network of small RNAs regulates sporulation initiation in C. difficile" describes the use of the global approach RIL-seq to study sRNA regulation in Clostridium difficile at an early stage of sporulation. Using Hfq as a bait, the method allows to identify the RNA-RNA interactome among those, sRNA-mRNA couples, whose pairing is dependent on the chaperone. The authors discovered two novel sRNA, SpoX and SpoY acting in an inverse manner on the same transcript, encoding the master regulator of sporulation Spo0A. Diverse in vitro and in vivo experiments concluded that SpoX activates spo0A translation and sporulation, probably by liberating the RBS, while in contrast SpoY binds close to the AUG and inhibits spo0A translation, and sporulation. Nevertheless, the mechanisms of regulation are not clear and should be investigated regarding the two possible TSS of the spo0A transcript. Finally, intraperitoneal mice model of infection revealed a global effect of both sRNA on gut colonization and intestinal infection. These results may also need further investigations. The method has been published in 2016 and was proven to be highly effective in E. coli and Salmonella to decipher sRNA regulatory network in bacteria. Because the proof of concept is well established for few years, my feeling is that the authors did not exploit and validate extensively their results. While the experiments are generally well conducted and described, I have comments and suggestions to hopefully improve the impact of the article.

Major comments

Q1: L95: in the RIL-seq data analysis, how were TSS considered, were they experimentally defined and particularly for sRNA studied in Figure 2B and SpoX, SpoY or both pTSS and sTSS of spo0A mRNA (Figure 3BD)?

Response: The TSS annotations for all sRNAs (shown in the manuscript) and *spo0A* are based on a previously published primary transcriptome annotation of *C. difficile* 630 using differential RNA-seq analysis in which we annotated transcription start sites and transcript ends on a genome-wide scale (https://www.pnas.org/doi/10.1073/pnas.2103579118). In addition, both the pTSS and sTSS of *spo0A* have been experimentally identified in an independent study (https://journals.asm.org/doi/10.1128/JB.00272-11). This has now been clarified in the revised manuscript (L89-90, L210-221) and is indicated in the figure legend for Figures 3.

Q2: L132: what was the rational in the choice of sRNAs? Are nc152 and 153 3'UTR-derived sRNA generated by maturation or are they bona fide ncRNAs? Why is nc155 not considered as an IGR?

Response: The sRNAs were prioritized based on the number of interactions we found in our RIL-seq data (sRNAs with the most interactions were selected). Information regarding TSS, processing and genomic location of all sRNAs is listed in Supplementary Table 4 and also highlighted in Figure 2C. In our previously published dRNA-seq dataset (see response above) we annotated a TSS both for nc152 and nc153, but nc152 seems to be further processed to a shorter isoform. nc155 is considered antisense, because it is encoded antisense to the copZ 3'UTR. A reference to this study has now been added to the figure legend of Figure 2.

Q3: L181: what about the mRNA expression of spo0A? did the two forms follow the same pattern of expression?

Response: Expression of *spo0A* has been addressed in several previous publications and is well established knowledge in the *C. difficile* community. Therefore, we did not investigate *spo0A* mRNA levels over growth in our study. In brief, two experimentally determined growth phase–dependent TSSs for *spo0A* have been identified that drive transcription of *spo0A* during exponential and stationary phases of growth. The primary TSS (= long 5UTR) is associated with

a SigA promoter sequence (housekeeping sigma factor), and the secondary TSS (= short 5UTR) is associated with a SigH promoter sequence (sigma factor of the transition phase):
https://www.pnas.org/doi/10.1073/pnas.2103579118
https://journals.asm.org/doi/10.1128/JB.00272-11
https://www.frontiersin.org/articles/10.3389/fmicb.2020.01939/full

In line with these transcriptional analyses, production of Spo0A protein throughout growth has been demonstrated as well:
https://www.microbiologyresearch.org/content/journal/micro/10.1099/mic.0.046243-0
https://www.nature.com/articles/s41598-021-86878-9
https://journals.plos.org/plosone/article?id=10.1371/journal.pone.0048608

In summary, Spo0A is rather 'constitutively' expressed and has a regulon that goes beyond sporulation-specific genes (https://bmcgenomics.biomedcentral.com/articles/10.1186/1471-2164-15-160).

Q4: Are transcripts affected by SpoX and SpoY regulation? Northern experiments in the wild-type and the two deletion mutants ΔspoX and ΔspoY strains may bring clues about the mechanisms of regulation.

Response: SpoX and SpoY do affect *spo0A* transcript levels post-transcriptionally as quantified by qPCR in Figure 6. These results are further outlined in the text (L282-285): "Interestingly, SpoY overexpression and SpoX deletion also resulted in a decrease in spo0A transcript levels, potentially by affecting ribosome occupancy of the spo0A mRNA, which can affect RNase mediated cleavage and thus mRNA stability[49,50]." This effect of sRNA regulators on transcript levels of regulated target genes can be observed frequently (https://pubmed.ncbi.nlm.nih.gov/16264189/). However, in *C. difficile* we can only speculate about the mechanism underlying the observed changes in transcript levels as functional studies of potential RNases are still entirely lacking.

Q5: L203: surprisingly base pairing predictions were presented with the short transcript of spo0A, while EMSA were performed with the long form of spo0A. Which form was used in the In-line probing assay?

Response: The interaction sites of SpoY and SpoX within the *spo0A* 5'UTR (indicated in Figure 3D) were derived from base-pairing predictions for the long *spo0A* 5'UTR (pTSS). Nevertheless, the predicted interaction sites and secondary structure of the *spo0A* RBS region are identical for the SpoX-*spo0A* duplex with both the long (pTSS) and the short (sTSS) *spo0A* 5'UTR. We have now changed the figure legend for Supplementary Figure 5B to indicate that the long *spo0A* (pTSS) was used. In line probing was also performed with the long *spo0A* 5'UTR, this is indicated in the materials and methods section (L756-757) and in the respective Figure legend for Figure 4.

Q6: The model of SpoY and SpoX binding to sTSS or pTSS is difficult to interpret and should be discussed.

Response: Neither SpoY nor SpoX bind the sTSS or pTSS of *spo0A.* Rather, we propose that SpoY and SpoX bind the 5'UTR of the *spo0A* mRNA and thus act as post-transcriptional regulators affecting translation, similar to the mechanism employed by most characterized bacterial sRNAs. Our RIL-seq data and *in silico* base-pairing predictions (Figure 3D) suggest interactions for both sRNAs downstream of the secondary TSS. This means that the SpoX and SpoY target sites are present in both the short and the long 5'UTR of *spo0A*. Although not shown in the original submission, we performed EMSAs with WT SpoY and SpoX and both *spo0A* 5'UTR lengths, respectively, and received similar results, indicating that SpoY or SpoX interact with

both *spo0A* 5'UTRs equally well. We have added these EMSA results in the revised manuscript (Supplementary Figure 6D, L220-224).

Q7: L217: The complex formation of SpoY with spo0A (pTSS) even in presence of Hfq is not convincing, free radiolabeled SpoY did not decrease. What about the affinity of SpoY with sTSS mRNA of spo0A?

Response: We agree that interaction between SpoY and *spo0A* is very weak. However, the signal clearly disappears when combining *spo0A* with a mutated SpoY and is somewhat rescued when introducing compensatory mutations to *spo0A* (Figure 4A & Supplementary Figure 6B). As stated above, we have now added EMSAs for interaction of both WT sRNAs with the short *spo0A* 5'UTR (Supplementary Figure 6D) and receive similar results as for the long 5'UTR. Taken together, we think the evidence is sufficient in particular in combination with the *in vivo* reporter assays (Figure 4C).

Q8: L232 (supp Figure 5D): which 5'UTR length of spo0A was used? The results with the spo0A fusion in combination with one SpoX or SpoY were not shown.

Response: The long *spo0A* 5'UTR was used. We have now properly indicated this in the method section (L502) and in the figure description for Suppl. Figure 7A. Regarding the second sentence, we are not entirely sure what the reviewer means. If this question is related to Supplementary Figure 5D (now Supplementary Figure 7A), this figure is a schematic representation and position of the sRNA, be it SpoX or SpoY, is indicated by "sRNA". We have now clarified this in the figure description, as well. If the question refers to Figure 4C, we hope the following explanation will answer the question: the red bars in both graphs of Figure 4C represent the results of co-expression of the Spo0A reporter with <u>either</u> SpoX <u>or</u> SpoY.

Q9: L242 (Figure 4C, right panel): the level of mCherry induced in presence of SpoX was not significantly different from the level in the ΔspoX background.

Response: We have added additional replicates to address this comment. mCherry fluorescence is now significantly different in the strain co-expressing SpoX and the *spo0A*-mCherry fusion, compared to the control only expressing the *spo0A*-mCherry fusion.

Q10: L257 (Figure 5A): This panel should be combined to Figure 4, as panel D. Then Figure 6 may be exchanged with Figure 5 to respect the order in the text.

Response: We have rearranged the Figures as follows: Figures 5A is now Figure 5, Figure 5B& C are Figure 7A&B and Figure 6 remains Figure 6. The order in the figures now follows the order in the text.

Q11: L266: SpoX and SpoY relative levels are missing and must be added to confirm that they are expressed in the sporulation conditions used in the experiments.

Response: We have verified SpoX and SpoY expression via NB analysis using the same RNA samples that were used for qPCR experiments in Figure 6. The new data can be found in Supplementary Figure 8A.

Q12: L295-298 (Figure 7D): the numbers of vegetative bacteria or spores were mostly not significant for the mice infected with the ΔspoX mutant compared to the mice infected with the wild-type, except at day 7. It is surprising, because the ΔspoX mutant had the most pronounced effect in spo0A regulation or sporulation. In contrast, the deletion of spoY had no effect on Spo0A synthesis and sporulation but was less infectious. Other targets of SpoX and SpoY identified in RIL-seq may be involved in the infection process as

proposed for cwp2 or cwpV. The manuscript might have a better impact if at least these two mRNAs are shown to be direct targets of SpoX and SpoY.

Response: We have addressed this comment using our translational reporter system to validate an additional predicted RIL-seq interaction for each sRNA: SpoX-*cwpV* and SpoY-*cwp2*. Quantification of the fluorescence signal of the mCherry fusions to *cwp2* and *cwpV*, respectively, revealed that SpoY inhibits *cwp2* translation, while SpoX acts as a positive regulator of *cwpV* translation (Supplementary Figure 10). The *in silico* predicted sRNA-target interaction site is shown as well. These data confirm SpoX as a positive regulator and SpoY as a negative regulator also for their additional targets. The data have been included into the results (L311-317) and discussion (L414-417) sections.

Q13: L304: the discussion section is rather short and speculative, and some questions could be addressed: what could be the transcriptional regulators of SpoX and Y? what is the purpose to inhibit and activate the same transcript at the same time (stage 0 in Figure 6)? which sRNA would come first? In the respective deletion mutants of SpoY or SpoX, we should see the effect of SpoX respectively SpoY, but they behave almost as WT, what are the suggestions?

Response: Regarding the transcriptional regulation of both sRNAs, we have profiled expression of both sRNAs in various media known to influence sporulation (see also Q2B, reviewer 1). These analyses have not provided further insight into potential regulators of SpoX and SpoY. Furthermore, we initially analysed the promoter sequence of both sRNAs, but there was no clear promoter motif detectable and we decided to avoid speculating. More comprehensive studies will be required to fully address this question. However, we have now included a section in the discussion to address this question in more detail.
We do not assume that SpoX and SpoY are expressed at the same time or regulate *spo0A* at the same time within a bacterial cell. We rather propose that they regulate the same stage of sporulation in distinct conditions. As discussed in the text (result as well as discussion section), expression profiles of both sRNAs actually indicate expression at different time points (L177-183, L193-197). Based on our results, we hypothesize that SpoY most likely suppresses spo0A translation during early growth stages, in conditions that favour active growth rather than sporulation. In contrast, SpoX expression indicates that it exerts its positive effect on *spo0A* translation mostly in late exponential and early stationary phase when the sporulation process initiates (see L379-390). Given that sporulation initiation is highly asynchronous within a population (i.e. only a small proportion of the population initiates sporulation) this means that both regulators can be active at the same time in different sub-populations of a culture. To really address this exciting but challenging question, high-resolution single-cell based assays will be required.

Q14: L660: the medium used in the northern blots must be provided. Expression varies depending on the nutrients.

Response: We have added missing information to the method section (L694-697) and to the respective figure legends.

Q15: L738-39 L748-750: were the conditions used in the reporter system assays (TY at 6 hours) the same condition as the RIL-seq (TY medium at OD 1,2)? please, indicate OD instead of timing.

Response: Conditions for reporter assays and RIL-seq were not the same. We have added this information to the method section (L778, L792-794). Since sRNA and mRNA expression is constitutive in the fluorescent reporter assays, we did not consider it necessary to use the same growth phase. To avoid confusion, we have now indicated the growth phase throughout the

manuscript (instead of OD/timepoint). Generally, OD/time point vary slightly between the RIL-seq experiment and, for example, the WB experiments where we used plasmid-bearing strains which needed to be kept under antibiotic selection. Nevertheless, figure 2B is intended to serve as reference for the various growth phases that were used throughout experiments. We have now indicated this in the text wherever appropriate.

Q16: L748-750: the culture conditions to verify sRNA expression (BHI, OD=1) must follow the conditions of the reporter assays. BHI contains glucose, in contrast to TY, expression of RNAs may vary. Moreover, the strain containing the mutated derivatives of SpoY and SpoX are missing (Fig S5E).

Response: We have repeated the experiment using the reporter assay conditions (TY medium) and included the missing strains (Supplementary Figure 7B&C).

Q17: L758-59: were the conditions used in the western blot comparable to the condition of the RIL-seq? please, indicate OD instead of timing. Northern blot showing the SpoX and SpoY should be added as controls.

Response: For WB analysis, cells were grown to either mid-exponential phase of growth (ME, $OD_{600} \sim 0.5$) in TY medium or stationary phase (ST, $OD_{600} \sim 1.3$) and thus slightly longer then for the RIL-seq experiment. We have clarified this in the method section (L576, L792-794). We did not attempt to exactly replicate RIL-seq conditions in the WB analysis of Spo0A expression, as the strains used for the respective experiments are different. In contrast to the RIL-seq experiment, we used sRNA deletion strains or strains constitutively overexpressing either SpoY or SpoX, thus mostly uncoupling sRNA expression from growth conditions. Northern blot validation of sRNA expression has now been added to Figure 5.

Minor comments

Q18: L122: 3'UTRs are also reservoirs of sRNA, any example known in C. difficile that could have been hit ?

Response: There are several 3'UTR encoded sRNAs known in *C. difficile* (previously published in https://www.pnas.org/doi/10.1073/pnas.2103579118). Several of those were found in our RIL-seq dataset, including nc28 and nc83. Please note that those 3'UTRs, found within chimeras, that harbour a predicted or validated sRNA are annotated as 'sRNA' in the RIL-seq dataset. Hence, for all the remaining 3'UTRs that were found in chimeras, we currently assume that the majority do not harbour an sRNA.

Q219: L215: 5A instead of 4A.

Response: No, 4A is correct.

Q20: L708/718/723: how can the authors measure the concentration of labelled sRNA, while the labelling is not 100% efficient, better to indicate the radioactive counts.

Response: This is a well established method, we followed previously published protocols for this experiment:
https://www.cell.com/molecular-cell/pdf/S1097-2765(21)01135-7.pdf
https://rnajournal.cshlp.org/content/14/4/685.full#sec-10

Q21: L902 and references' list: all the bacterial names should be italized.

Response: The references have been edited accordingly.

Referee #3:

Fuchs et al. presents a well-written, intriguing story that will appeal to a broad audience. Briefly, they performed C. difficile RIL-seq to identify RNA interacting partners of Hfq-associated sRNAs in rich medium. They discovered numerous novel RNA-RNA interactions and identified several novel sRNAs. They also created a searchable database for their data sets which will be of interest within the Clostridial community. Further, they detailed the mechanistic role of two sRNAs, renamed SpoX and SpoY, that directly interact with the spo0A mRNA to post-transcriptionally regulate its expression and influence sporulation. Finally, they provided some evidence that SpoX and SpoY may impact colonization and spore burden in a mouse model. This study lacks some controls and needs to ensure the correct number of independent biological replicates are performed (see comments). Finally, mRNA stability studies to support or exclude whether the sRNAs affect spo0A transcript stability or translation would strengthen the mechanistic studies, and in vitro toxin analyses would provide more context for the animal studies.

Major comments

Q1: Fig. 4C /Lines 248-249 - The translational mCherry fusions confirm that SpoX and SpoY's affects do occur post-transcriptionally and may suggest that SpoX and SpoY affect spo0A translation, but these experiments do not exclude the possibility that SpoX and (less likely) SpoY impact spo0A mRNA stability rather than translation. spo0A mRNA stability experiments in the parent strain and the respective spoX and spoY mutants would rule out this possibility.

Response: We agree and have actually addressed this question in Figure 6. A clear impact on *spo0A* transcript levels can be seen in the SpoY overexpression and SpoX deletion strain. We have clarified this in the manuscript as follows "Interestingly, SpoY overexpression and SpoX deletion also resulted in a decrease in spo0A transcript levels, potentially by affecting ribosome occupancy of the spo0A mRNA, which can affect RNase mediated cleavage and thus mRNA stability[49,50]" (L282-285).

Q2: Fig. 5A - An additional replicate needs to be performed for an n = 3 as is standard practice within the field and is required for reproducibility and rigor.

Response: We agree and have added a third biological replicate.

Q3: Fig. 5BC - Sporulation assays and phase-contrast microscopy: Generally, biological triplicates performed simultaneously are not standard in the field for rigor. Three independent experiments (one biological replicate per experiment is adequate) need to be performed to ensure reproducibility and rigor.

Response: We have repeated the experiment as suggested with 3 three independent biological replicates (= one replicate/day) (Figure 7 and Supplementary Figure 8). The new experiments confirm our initial results that SpoX increases sporulation rates whereas SpoY acts as a negative regulator of sporulation.

Q4: Lines 367-368 - Spo0A impacts on toxin production seem to be variable in the 012 ribotype, depending on the conditions and the specific strains tested (e.g. Underwood et al used 630Δerm, not 630). Because animal studies suggest that toxin production is not affected by the spoX or spoY mutation, one optional consideration is to perform quick TcdA/TcdB ELISAs with the parent strain and the spoX/spoY mutants in vitro to see if toxin production in these mutants correlates with the indirect results seen in the animal studies.

Response: As pointed out by the reviewer, Spo0A mediated regulation of toxin production seems to be highly strain dependent and is a controversial topic in the *C. difficile* community. While Underwood *et al.* observed a decrease in toxin production in a *C. difficile* 630 Δerm Δ*spo0A* deletion strain (https://journals.asm.org/doi/10.1128/JB.00882-09), Mackin *et al.* and Rosenbusch *et al.* observed no differences in toxin expression in a *C. difficile* 630 Δerm Δ*spo0A* mutant (https://www.ncbi.nlm.nih.gov/pmc/articles/PMC3827441/, https://www.ncbi.nlm.nih.gov/pmc/articles/PMC3485338/) and Pettit *et al.* reported an upregulation of *tcdA* (toxin A) transcript levels (https://pubmed.ncbi.nlm.nih.gov/24568651/).

We have performed *in vitro* toxin ELISAs (simultaneous quantification of TcdA and TcdB) as suggested by the reviewer. Firstly, *in vitro* quantification of toxin levels indicated increased toxin levels in culture supernatants of *C. difficile 630* Δ*spo0A* compared to the WT at the 48 h time point (Figure R1A). This is in contrast to most of the previous studies (mentioned above) and confirms the high strain-dependence of this phenotype. Deletion of SpoY resulted in a decrease in toxin levels in the supernatant, while SpoX deletion leads to increased toxin levels (Figure R1A&B). These results suggest that the observed changes in toxin levels upon sRNA deletion could be a downstream effect of Spo0A regulation by SpoX/SpoY. However, these differences in toxin production could equally be the effect of other sRNA-target interactions in addition to or independent of the SpoX/SpoY-*spo0A* interaction.

Regarding the mouse data: the reduction in toxin levels in the Δ*spoY* mutant might explain the impaired colonization in the mouse model. But for SpoX the explanation is less straight forward. A comprehensive set of experiments would be necessary to fully understand the impact of SpoY and SpoX on toxin production in *C. difficile* 630, especially during gut colonization. Accordingly, we have decided to not include the results in the manuscript, but rather present them here to explain our reasoning and to respond to the reviewer's question.

Q5: Lines 387-388 - The cwp2 promoter is used to control spo0A 5' UTR transcription in the mCherry constructs even though cwp2 is a potential SpoY target. Are any of the cwp2 regions predicted to interact with SpoY included in these promoter constructs?

Response: sRNA mediated regulation occurs on the post-transcriptional level (between a sRNA and the mRNA of the target gene) and is thus independent of the promoter region, which is not transcribed into mRNA. Accordingly, the RIL-seq predicted interaction between SpoY and *cwp2* lies in the 5'UTR region of the cwp2 mRNA. In addition, we have performed *in silico* predictions using IntaRNA and could not find any predicted interaction site(s) in the promoter region of *cwp2*.

Q6: Fig. 7D - Enumeration of spores from mice feces - were heat-treated fecal samples also plated on bioMérieux C. difficile agar without taurocholate supplementation to ensure all vegetative cells were eliminated? It can be difficult to kill all vegetative cells in animal feces with heat treatment, and this is an important control. Especially given the sporulation frequency results *in vitro* with the spoX mutant, fewer spores would have been anticipated in the mouse model.

Response: To establish the protocol in our lab, we had initially performed all necessary controls to ensure that heat treatment is generally efficient in our hands – this included plating of heat-treated faecal samples on bioMérieux agar with and without taurocholate (TCA). Therefore, for this particular experiment, we had not included this control. We acknowledge the importance of this control and have therefore repeated the infection with the WT strain (n = 5 mice) to rule out the possibility that significant amounts of vegetative cells survive the heat treatment (Figure R2). According to our results, heat treatment results in an approximately 1000-fold reduction of CFU counts when samples were plated on bioMérieux plates without TCA compared to heat-treated samples plated on bioMérieux plates supplemented with TCA. Given the complex composition of the bioMérieux medium, these very few remaining colonies likely represent spontaneous germination events of individual spores.

Minor comments:

Q7: Figure 2A - What are the red shaded areas in the operons of the sRNAs nc152, nc153, and nc 155?

Response: Untranslated regions (3'UTRs), as indicated in the figure description.

Q8: Figure 2C - It would be helpful to include the identity of the sRNA that interacts with each intergenic region (e.g. CDIF630nc_161 interacting with sigG) in the figure panel.

Response: We have added this information to the figure.

Q9: Line 204 - Define "seed region" for a broader audience that may be unfamiliar with sRNA terminology.

Response: We have added this information to the text (L209).

Q10: Lines 262-281 - Discuss the data in the order they appear in the results, so please move the sporulation frequency data up in the results sections and move the qRT-PCR results below that section.

Response: We have rearranged the Figures to match the text flow: Figure 5A is now Figure 5, Figures 5B&C are now Figure 7, while Figure 6 remains as before.

Q11: Line 345-347 - Regarding the minor phenotype in the spoY mutant - is it possible that the negative effects of SpoY are masked in the presence of SpoX (especially given the regions spanned in the chimera reads); however, the impact of SpoY and SpoX may also be due to growth phase dependent expression based on northern blotting. These possibilities should also be addressed in the discussion.

Response: In our manuscript, we hypothesize that the minor phenotype of the SpoY mutant might be the result of redundant sRNA regulators. In fact, we identified eight additional sRNAs that displayed a chimeric read profile comparable to SpoY. This strongly suggests a similar negative regulatory mode of action for these sRNAs. We have outlined this possibility in the discussion (L358-364). A growth phase dependent impact of SpoY and SpoX on *spo0A* (in the WT) as well as a potential sponging effect of either SpoX or SpoY has also been discussed in the text (L379-390, L391-404).

Line 765 - Instead of 340 mM, do you mean 340 mA?

Response: Yes. We have changed the text accordingly.

1st Revision - Editorial Decision 21st Mar 2023

Dear Franziska,

Thank you for submitting a revised version of your manuscript. Your study has now been seen by two of the original referees, who find that most of their previous concerns have been addressed and now recommend publication of the manuscript after minor edits in the figure 9 (referee #3). There remain also a few editorial points that have to be addressed before I can extend formal acceptance of the manuscript:

1. Our publisher has done their pre-publication check on your manuscript. I have attached the file here. Please take a look at the word file and the comments regarding the figure legends and respond to the issues. Please also use this version when you resubmit the revised version.
2. Please limit the number of keywords to five.
3. There are some issues with the order of figure panel callouts in the manuscript text: callout for Figure 2B should be after 2A; 5B should be after 5A; 9C should be after 9B; EV2C should be after EV2B. Figure panels 7A, 7B, EV4A, EV4B are not mentioned in the manuscript text.
4. Please rename Appendix Tables S1-S5 to Dataset EV1-EV5, and S6-S7 to Appendix Table S1-S2. Please update the corresponding callouts in the text.
5. Please upload Datasets EV1-5 as individual files. Their legends should be removed from Appendix file and included in each Excel file as a separate tab.
6. CRediT has replaced the traditional author contributions section because it offers a systematic, machine-readable author contributions format that allows for more effective research assessment. Please remove the Authors Contributions from the manuscript and use the free text boxes beneath each contributing author's name in our online submission system to add specific details on the author's contribution. More information is available in our guide to authors.

Please let me know if you have any questions regarding these points. You can use the link below to upload the revised files.

Thank you again for giving us the chance to consider your manuscript for The EMBO Journal. I look forward to receiving the final version.

With best regards,

Ieva

Ieva Gailite, PhD
Senior Scientific Editor
The EMBO Journal
Meyerhofstrasse 1
D-69117 Heidelberg
Tel: +4962218891309
i.gailite@embojournal.org

Further information is available in our Guide For Authors: https://www.embopress.org/page/journal/14602075/authorguide

We realize that it is difficult to revise to a specific deadline. In the interest of protecting the conceptual advance provided by the work, we recommend a revision within 3 months (19th Jun 2023). Please discuss the revision progress ahead of this time with the editor if you require more time to complete the revisions.

Referee #2:

First, I would like to acknowledge that the authors have added many experiments to reply to the reviewers' remarks. The manuscript is now more complete and on my side they have answered all my suggestions and comments.

Referee #3:

Fuchs et al has sufficiently and thoroughly responded to all of the reviewer's comments, and this well-written, informative

manuscript is ready for publication after a minor adjustment to Figure 9C. The author's depict what is known thus far about sporulation initiation in panel C. I assume Sig0E refers to the phosphatase Spo0E? If so, please change Sig0E to Spo0E. Finally, RstA has been shown to positively control sporulation, but there is no evidence that RstA negatively controls the activity of any of the histidine kinases. This interaction might be more appropriately depicted at this time as a dotted arrow directly to Spo0A.

The authors have addressed all minor editorial requests.

2nd Revision - Editorial Decision 29th Mar 2023

Dear Franziska,

Thank you for addressing the final editorial points. I am now pleased to inform you that your manuscript has been accepted for publication.

Before we forward your manuscript to our publishers, I would like to propose a couple of minor changes in the article title, synopsis and abstract to enhance their accessibility to our more general readership. I have also written a short blurb that will accompany the title of your manuscript in our online table of contents. Please take a look at the text below and in the attached manuscript text file and let me know if any corrections are necessary.

Title:
A network of small RNAs regulates sporulation initiation in Clostridioides difficile

Blurb:
Small RNA/target interaction mapping in the enteric pathogen C. difficile reveals the first RNA chaperone Hfq-dependent RNA interactome in a gram-positive bacterium.

Synopsis authors:
RIL-seq has revealed the RNA chaperone Hfq-dependent interactome of small RNAs and their targets in gram-negative bacteria. Here, application of RIL-seq in the human pathogen C. difficile uncovers the first Hfq-dependent RNA interactome in a gram-positive bacterium and identifies a pair of small regulatory RNAs that modulate sporulation initiation.
• RIL-seq in C. difficile strain 630 identifies Hfq-associated sRNA-mRNA pairs
• RIL-seq analysis identifies the master regulator of sporulation, Spo0A, as a central target of sRNA-based regulation.
• The small RNAs SpoY and SpoX act as negative and positive regulators, respectively, of sporulation by base-pairing with the spo0A mRNA.
• Deletion of either SpoY or SpoX affects gut colonization in a mouse model of C. difficile-induced colitis.

Please note that it is EMBO Journal policy for the transcript of the editorial process (containing referee reports and your response letter) to be published as an online supplement to each paper. If you do NOT want this, you will need to inform the Editorial Office via email immediately. More information is available here:
https://www.embopress.org/page/journal/14602075/authorguide#transparentprocess

Your manuscript will be processed for publication in the journal by EMBO Press. Manuscripts in the PDF and electronic editions of The EMBO Journal will be copy edited, and you will be provided with page proofs prior to publication. Please note that supplementary information is not included in the proofs.

You will be contacted by Wiley Author Services to complete licensing and payment information. The required 'Page Charges Authorization Form' is available here: https://www.embopress.org/pb-assets/embo-site/tej_apc.pdf - please download and complete the form and return to embopressproduction@wiley.com

EMBO Press participates in many Publish and Read agreements that allow authors to publish Open Access with reduced/no publication charges. Check your eligibility: https://authorservices.wiley.com/author-resources/Journal-Authors/open-access/affiliation-policies-payments/index.html

Should you be planning a Press Release on your article, please get in contact with embojournal@wiley.com as early as possible, in order to coordinate publication and release dates.

If you have any questions, please do not hesitate to call or email the Editorial Office. Thank you for this contribution to The EMBO Journal and congratulations on a successful publication!

Best regards,

Ieva

Ieva Gailite, PhD
Senior Scientific Editor
The EMBO Journal
Meyerhofstrasse 1
D-69117 Heidelberg
Tel: +4962218891309
i.gailite@embojournal.org

EMBO Press Author Checklist

Corresponding Author Name: Franziska Faber	
Journal Submitted to: The EMBO Journal	
Manuscript Number: EMBOJ-2022-112858	

USEFUL LINKS FOR COMPLETING THIS FORM
The EMBO Journal - Author Guidelines
EMBO Reports - Author Guidelines
Molecular Systems Biology - Author Guidelines
EMBO Molecular Medicine - Author Guidelines

Reporting Checklist for Life Science Articles (updated January
This checklist is adapted from Materials Design Analysis Reporting (MDAR) Checklist for Authors. MDAR establishes a minimum set of requirements in transparent reporting in the life sciences (see Statement of Task: 10.31222/osf.io/9sm4x). Please follow the journal's guidelines in preparing your
Please note that a copy of this checklist will be published alongside your article.

Abridged guidelines for figures
1. Data
The data shown in figures should satisfy the following conditions:
- the data were obtained and processed according to the field's best practice and are presented to reflect the results of the experiments in an accurate and unbiased manner.
- ideally, figure panels should include only measurements that are directly comparable to each other and obtained with the same assay.
- plots include clearly labeled error bars for independent experiments and sample sizes. Unless justified, error bars should not be shown for technical
- if n<5, the individual data points from each experiment should be plotted. Any statistical test employed should be justified.
- Source Data should be included to report the data underlying figures according to the guidelines set out in the authorship guidelines on Data

2. Captions
Each figure caption should contain the following information, for each panel where they are relevant:
- a specification of the experimental system investigated (eg cell line, species name).
- the assay(s) and method(s) used to carry out the reported observations and measurements.
- an explicit mention of the biological and chemical entity(ies) that are being measured.
- an explicit mention of the biological and chemical entity(ies) that are altered/varied/perturbed in a controlled manner.
- the exact sample size (n) for each experimental group/condition, given as a number, not a range;
- a description of the sample collection allowing the reader to understand whether the samples represent technical or biological replicates (including how many animals, litters, cultures, etc.).
- a statement of how many times the experiment shown was independently replicated in the laboratory.
- definitions of statistical methods and measures:
  - common tests, such as t-test (please specify whether paired vs. unpaired), simple χ2 tests, Wilcoxon and Mann-Whitney tests, can be unambiguously identified by name only, but more complex techniques should be described in the methods section;
  - are tests one-sided or two-sided?
  - are there adjustments for multiple comparisons?
  - exact statistical test results, e.g., P values = x but not P values < x;
  - definition of 'center values' as median or average;
  - definition of error bars as s.d. or s.e.m.

Please complete ALL of the questions below.
Select "Not Applicable" only when the requested information is not relevant for your study.

Materials

Newly Created Materials	Information included in the manuscript?	In which section is the information available? (Reagents and Tools Table, Materials and Methods, Figures, Data Availability Section)
New materials and reagents need to be available; do any restrictions apply?	Not Applicable	

Antibodies	Information included in the manuscript?	In which section is the information available? (Reagents and Tools Table, Materials and Methods, Figures, Data Availability Section)
For **antibodies** provide the following information:<br>- Commercial antibodies: RRID (if possible) or supplier name, catalogue number and or/clone number<br>- Non-commercial: RRID or citation	Yes	Materials and Methods

DNA and RNA sequences	Information included in the manuscript?	In which section is the information available? (Reagents and Tools Table, Materials and Methods, Figures, Data Availability Section)
**Short novel DNA or RNA including primers, probes**: provide the sequences.	Yes	Supplementary Information

Cell materials	Information included in the manuscript?	In which section is the information available? (Reagents and Tools Table, Materials and Methods, Figures, Data Availability Section)
**Cell lines:** Provide species information, strain. Provide accession number in repository **OR** supplier name, catalog number, clone number, and/**OR** RRID.	Not Applicable	
**Primary cultures:** Provide species, strain, sex of origin, genetic modification status.	Not Applicable	
Report if the cell lines were recently **authenticated** (e.g., by STR profiling) and tested for mycoplasma contamination.	Not Applicable	

Experimental animals	Information included in the manuscript?	In which section is the information available? (Reagents and Tools Table, Materials and Methods, Figures, Data Availability Section)
**Laboratory animals or Model organisms:** Provide species, strain, sex, age, genetic modification status. Provide accession number in repository **OR** supplier name, catalog number, clone number, **OR** RRID.	Yes	Materials and Methods
**Animal observed in or captured from the field:** Provide species, sex, and age where possible.	Not Applicable	
Please detail **housing and husbandry conditions**.	Yes	Materials and Methods

Plants and microbes	Information included in the manuscript?	In which section is the information available? (Reagents and Tools Table, Materials and Methods, Figures, Data Availability Section)
**Plants:** provide species and strain, ecotype and cultivar where relevant, unique accession number if available, and source (including location for collected wild specimens).	Not Applicable	
**Microbes:** provide species and strain, unique accession number if available, and source.	Yes	Materials and Methods, Supplementary Information

Human research participants	Information included in the manuscript?	In which section is the information available? (Reagents and Tools Table, Materials and Methods, Figures, Data Availability Section)
If collected and within the bounds of privacy constraints report on age, sex and gender or ethnicity for all study participants.	Not Applicable	

Core facilities	Information included in the manuscript?	In which section is the information available? (Reagents and Tools Table, Materials and Methods, Figures, Data Availability Section)
If your work benefited from core facilities, was their service mentioned in the acknowledgments section?	Not Applicable	

Design

Study protocol	Information included in the manuscript?	In which section is the information available? (Reagents and Tools Table, Materials and Methods, Figures, Data Availability Section)

If study protocol has been **pre-registered, provide DOI in the manuscript**. For clinical trials, provide the trial registration number **OR** cite DOI.	Not Applicable	
Report the **clinical trial registration number** (at ClinicalTrials.gov or equivalent), where applicable.	Not Applicable	

Laboratory protocol	Information included in the manuscript?	In which section is the information available? (Reagents and Tools Table, Materials and Methods, Figures, Data Availability Section)
Provide DOI OR other citation details if **external detailed step-by-step protocols** are available.	Yes	Materials and Methods

Experimental study design and statistics	Information included in the manuscript?	In which section is the information available? (Reagents and Tools Table, Materials and Methods, Figures, Data Availability Section)
Include a statement about **sample size** estimate even if no statistical methods were used.	Yes	Main Manuscript, Figure legends, Materials and Methods
Were any steps taken to minimize the effects of subjective bias when allocating animals/samples to treatment (e.g. **randomization procedure**)? If yes, have they been described?	Not Applicable	
Include a statement about **blinding** even if no blinding was done.	Not Applicable	
Describe **inclusion/exclusion criteria** if samples or animals were excluded from the analysis. Were the criteria pre-established?<br><br>If sample or data points were omitted from analysis, report if this was due to attrition or intentional exclusion and provide justification.	Not Applicable	
For every figure, are **statistical tests** justified as appropriate? Do the data meet the assumptions of the tests (e.g., normal distribution)? Describe any methods used to assess it. Is there an estimate of variation within each group of data? Is the variance similar between the groups that are being statistically compared?	Yes	Figure legends

Sample definition and in-laboratory replication	Information included in the manuscript?	In which section is the information available? (Reagents and Tools Table, Materials and Methods, Figures, Data Availability Section)
In the figure legends: state number of times the experiment was **replicated** in laboratory.	Yes	Main Manuscript, Figure legends, Materials and Methods
In the figure legends: define whether data describe **technical or biological replicates**.	Yes	Main Manuscript, Figure legends, Materials and Methods

Ethics

Ethics	Information included in the manuscript?	In which section is the information available? (Reagents and Tools Table, Materials and Methods, Figures, Data Availability Section)
Studies involving **human participants**: State details of **authority granting ethics approval** (IRB or equivalent committee(s), provide reference number for approval.	Not Applicable	
Studies involving **human participants**: Include a statement confirming that **informed consent** was obtained from all subjects and that the experiments conformed to the principles set out in the WMA Declaration of Helsinki and the Department of Health and Human Services Belmont Report.	Not Applicable	
Studies involving **human participants:** For publication of **patient photos**, include a statement confirming that consent to publish was obtained.	Not Applicable	
Studies involving experimental **animals**: State details of **authority granting ethics approval** (IRB or equivalent committee(s), provide reference number for approval. Include a statement of compliance with ethical regulations.	Yes	Materials and Methods
Studies involving **specimen and field samples:** State if relevant **permits** obtained, provide details of authority approving study; if none were required, explain why.	Not Applicable	

Dual Use Research of Concern (DURC)	Information included in the manuscript?	In which section is the information available? (Reagents and Tools Table, Materials and Methods, Figures, Data Availability Section)
Could your study fall under dual use research restrictions? Please check biosecurity documents and list of **select agents and toxins** (CDC): https://www.selectagents.gov/sat/list.htm	Not Applicable	
If you used a select agent, is the security level of the lab appropriate and reported in the manuscript?	Not Applicable	
If a study is subject to dual use research of concern regulations, is the name of the **authority granting approval and reference number** for the regulatory approval provided in the manuscript?	Not Applicable	

Reporting

The MDAR framework recommends adoption of discipline-specific guidelines, established and endorsed through community initiatives. Journals have their own policy about requiring specific guidelines and recommendations to complement MDAR.

Adherence to community standards	Information included in the manuscript?	In which section is the information available? (Reagents and Tools Table, Materials and Methods, Figures, Data Availability Section)
State if relevant guidelines or checklists (e.g., **ICMJE, MIBBI, ARRIVE, PRISMA**) have been followed or provided.	Not Applicable	
For **tumor marker prognostic studies,** we recommend that you follow the **REMARK** reporting guidelines (see link list at top right). See author guidelines, under 'Reporting Guidelines'. Please confirm you have followed these guidelines.	Not Applicable	
For **phase II and III randomized controlled trials**, please refer to the **CONSORT** flow diagram (see link list at top right) and submit the CONSORT checklist (see link list at top right) with your submission. See author guidelines, under 'Reporting Guidelines'. Please confirm you have submitted this list.	Not Applicable	

Data Availability

Data availability	Information included in the manuscript?	In which section is the information available? (Reagents and Tools Table, Materials and Methods, Figures, Data Availability Section)
Have **primary datasets** been deposited according to the journal's guidelines (see 'Data Deposition' section) and the respective accession numbers provided in the Data Availability Section?	Yes	Data Availability Section
Were **human clinical and genomic datasets** deposited in a public access-controlled repository in accordance to ethical obligations to the patients and to the applicable consent agreement?	Not Applicable	
Are **computational models** that are central and integral to a study available without restrictions in a machine-readable form? Were the relevant accession numbers or links provided?	Not Applicable	
If publicly available data were reused, provide the respective **data citations in the reference list**.	Not Applicable	
